# Arc Requires PSD95 for Assembly into Postsynaptic Complexes Involved with Neural Dysfunction and Intelligence

**DOI:** 10.1016/j.celrep.2017.09.045

**Published:** 2017-10-17

**Authors:** Esperanza Fernández, Mark O. Collins, René A.W. Frank, Fei Zhu, Maksym V. Kopanitsa, Jess Nithianantharajah, Sarah A. Lemprière, David Fricker, Kathryn A. Elsegood, Catherine L. McLaughlin, Mike D.R. Croning, Colin Mclean, J. Douglas Armstrong, W. David Hill, Ian J. Deary, Giulia Cencelli, Claudia Bagni, Menachem Fromer, Shaun M. Purcell, Andrew J. Pocklington, Jyoti S. Choudhary, Noboru H. Komiyama, Seth G.N. Grant

**Affiliations:** 1 Genes to Cognition Programme, The Wellcome Trust Sanger Institute, Hinxton, Cambridgeshire, UK; 2 Proteomic Mass Spectrometry, The Wellcome Trust Sanger Institute, Hinxton, Cambridgeshire, UK; 3 Medical Research Council Laboratory of Molecular Biology, Cambridge, UK; 4 Genes to Cognition Programme, Centre for Clinical Brain Science, University of Edinburgh, Edinburgh, UK; 5 Synome Ltd., Moneta Building, Babraham Research Campus, Cambridge, UK; 6 School of Informatics, Institute for Adaptive and Neural Computation, University of Edinburgh, UK; 7 Centre for Cognitive Ageing and Cognitive Epidemiology, Department of Psychology, University of Edinburgh, UK; 8 Analytic and Translational Genetics Unit, Massachusetts General Hospital, Boston, MA 02114, USA; 9 Stanley Center for Psychiatric Research, Broad Institute of MIT and Harvard, Cambridge, MA 02142, USA; 10 Division of Psychiatric Genomics, Department of Psychiatry, Icahn School of Medicine at Mount Sinai, New York, NY 10029, USA; 11 Institute of Psychological Medicine & Clinical Neurosciences, University of Cardiff, Cardiff, Wales, UK; 12 KU Leuven, Center for Human Genetics and Leuven Institute for Neurodegenerative Diseases (LIND), and VIB Center for the Biology of Disease, Leuven, Belgium; 13 Department of Biomedicine and Prevention, University of Rome Tor Vergata, Rome, Italy

**Keywords:** tandem affinity purification, PSD95, Arc, synaptic complexes, supercomplexes, genetic variants, cognition, intellectual disability, schizophrenia

## Abstract

Arc is an activity-regulated neuronal protein, but little is known about its interactions, assembly into multiprotein complexes, and role in human disease and cognition. We applied an integrated proteomic and genetic strategy by targeting a tandem affinity purification (TAP) tag and Venus fluorescent protein into the endogenous *Arc* gene in mice. This allowed biochemical and proteomic characterization of native complexes in wild-type and knockout mice. We identified many Arc-interacting proteins, of which PSD95 was the most abundant. PSD95 was essential for Arc assembly into 1.5-MDa complexes and activity-dependent recruitment to excitatory synapses. Integrating human genetic data with proteomic data showed that Arc-PSD95 complexes are enriched in schizophrenia, intellectual disability, autism, and epilepsy mutations and normal variants in intelligence. We propose that Arc-PSD95 postsynaptic complexes potentially affect human cognitive function.

## Highlights

• TAP tag and purification of endogenous Arc protein complexes from the mouse brain
• PSD95 is the major Arc binding protein, and both assemble into 1.5-MDa supercomplexes
• PSD95 is essential for recruitment of Arc to synapses
• Mutations and genetic variants in Arc-PSD95 are linked to cognition

## Graphical Abstract

**Figure:**
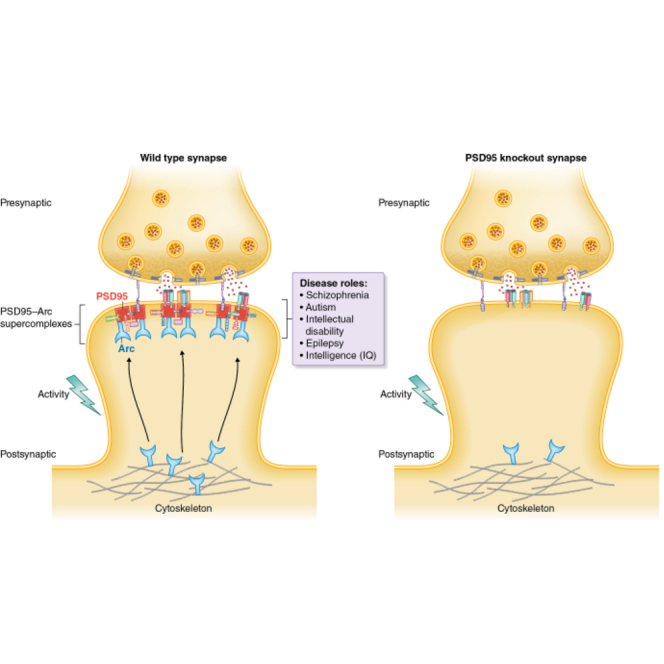

## Teaser

Fernández et al. use genetics and proteomics to study the Arc protein in the mouse brain. PSD95 recruits Arc to the synapse and assembles it into signaling complexes with neurotransmitter receptors and other proteins. Arc-PSD95 supercomplexes contain genetic variants previously linked to epilepsy, schizophrenia, intellectual disability, and IQ.

## Introduction

Arc/Arg3.1 was originally identified as a cytoskeletal-associated protein encoded by an mRNA that was rapidly transcribed following synaptic activity and transported to dendrites (Link et al., 1995, Lyford et al., 1995, Moga et al., 2004, Steward et al., 1998). Many forms of neuronal activation induce Arc: synaptic stimulation, including long-term potentiation (Guzowski et al., 2000); metabotropic glutamate receptor-dependent long-term depression (Jakkamsetti et al., 2013, Park et al., 2008, Waung et al., 2008); homeostatic scaling of AMPA receptors (Gao et al., 2010, Korb et al., 2013, Okuno et al., 2012, Shepherd et al., 2006); generalized neuronal activity induced by seizures (Link et al., 1995); as well as various behavioral stimuli (memory- and experience-related behavioral patterns; Daberkow et al., 2007, Gao et al., 2010, Guzowski et al., 1999, Jakkamsetti et al., 2013, Kelly and Deadwyler, 2003, Miyashita et al., 2009, Vazdarjanova and Guzowski, 2004, Vazdarjanova et al., 2006, Wibrand et al., 2012) and visual stimuli (Wang et al., 2006). Knockout or knockdown of Arc results in impaired synaptic plasticity and hippocampus-dependent learning and behavior phenotypes reminiscent of schizophrenia (Guzowski et al., 2000, Managò et al., 2016, McCurry et al., 2010, Plath et al., 2006, Wang et al., 2006).

Arc is mainly localized at postsynaptic sites of excitatory synapses (Moga et al., 2004). The proteome of the postsynaptic terminal of excitatory synapses of vertebrate species contains a highly conserved set of ∼1,000 protein types (Bayés et al., 2011, Bayés et al., 2012, Bayés et al., 2017, Distler et al., 2014) organized into more than 200 multiprotein complexes (Frank and Grant, 2017, Frank et al., 2016, Frank et al., 2017). The multiprotein complexes are organized into a hierarchy of complexes and supercomplexes (complexes of complexes), and the prototype supercomplex is formed by PSD95 (Fernández et al., 2009, Frank et al., 2016, Frank et al., 2017, Husi and Grant, 2001, Husi et al., 2000). Arc was found to be associated with PSD95 (Fernández et al., 2009, Frank et al., 2016, Frank et al., 2017, Husi et al., 2000), and genetic studies show that absence of either PSD95 or Arc leads to enhanced long-term potentiation (LTP) and impaired hippocampus-dependent learning (Migaud et al., 1998, Plath et al., 2006). Biochemical purification and mouse genetic experiments show that dimers of PSD95 assemble with multiple complexes, including NMDA receptors, potassium channels, and signaling and adhesion proteins. These are not all found within a single supercomplex but are within an extended family of PSD95 supercomplexes ranging in size from 1–3 MDa (Frank and Grant, 2017, Frank et al., 2016, Frank et al., 2017). A large-scale mouse genetic screen of more than 50 postsynaptic proteins found that PSD95 and its close interacting proteins had the strongest phenotypes in synaptic electrophysiology and behavior, indicating that PSD95 supercomplexes are crucial components of the postsynaptic terminal of excitatory synapses (N.H.K., L.N. van de Lagemaat, L.E. Stanford, C.M. Pettit, D.J. Strathdee, K.E. Strathdee, D.G.F., E.J. Tuck, K.A.E., T.J. Ryan, J.N., N.G. Skene, M.D.R.C., and S.G.N.G., unpublished data; M.V.K., L.N. van de Lagemaat, N. Afinowi, D.J. Strathdee, K.E. Strathdee, D.G.F., E.J. Tuck, K.A.E., N.G. Skene, M.D.R.C., N.H.K., and S.G.N.G., unpublished data). Arc has also been proposed to interact with the endocytic machinery (Dynamin and Endophilin-2 and -3) (Chowdhury et al., 2006, Rial Verde et al., 2006, Shepherd et al., 2006). However, Arc multiprotein complexes have not been purified and systematically studied using proteomic mass spectrometry, and thus the identity of its interacting partners and the composition of Arc complexes remain poorly understood.

Characterizing protein complexes in synapses is technically challenging. Gene-tagging of endogenous proteins in the mouse has greatly facilitated purification of intact native complexes and visualization of their subcellular localization and has many advantages over in vitro and recombinant methods (Broadhead et al., 2016, Fernández et al., 2009, Frank and Grant, 2017, Frank et al., 2016). The effect of mutations on complexes and neuronal activation can be combined in mice carrying knockin gene tags, and proteins that are predicted to be largely unstructured and form multivalent interactions, such as Arc (Xue et al., 2010), can be studied in their native context. These advantages have been illustrated by the purification of native NMDA receptor and PSD95 complexes, in which a tandem affinity purification (TAP) tag was inserted into the N terminus of the GluN1 subunit and C terminus of PSD95 by genome engineering (Fernández et al., 2009, Frank et al., 2016). Purification revealed that NMDA receptors and PSD95 were in ∼1.5-MDa supercomplexes with channel subunits, PSD95, and PSD93 as major components. Genetic dissection in vivo using mutant mice showed an essential tripartite requirement for PSD95, PSD93, and the GluN2B cytoplasmic domain (Frank et al., 2016). This tripartite interaction was not previously detected using in vitro methods, which typically rely on binary protein interactions. Moreover, like Arc, the GluN2B cytoplasmic domain is predicted to be a structurally unfolded/disordered domain (Ryan et al., 2008), and these domains lack stable tertiary structure and undergo disorder-to-order transitions upon binding or changes in phosphorylation (Bah et al., 2015, Gibbs et al., 2017). We therefore considered that Arc was well suited to the strategy of gene tagging and genetic dissection.

Genetics has been a powerful approach for studying the function of multiprotein complexes in many prokaryotic, eukaryotic, and metazoan organisms, including humans, where disease-causing mutations have been mapped to protein complexes (Babu et al., 2014, Lu et al., 2013, Vidal et al., 2011). Moreover, in recent years, a large number of mutations that disrupt postsynaptic proteins in humans have been identified and found to cause many psychiatric, neurological, and developmental disorders (Bayés et al., 2011, Bayés et al., 2014, Brose et al., 2010, Fromer et al., 2014, Grant, 2012, Grant, 2013, Grant et al., 2005, Kirov et al., 2012, Pocklington et al., 2006, Purcell et al., 2014). Although mutations in the human *ARC* gene have not been directly linked to any mental disorder, using preliminary proteomic data on Arc-interacting proteins, the proteins in Arc complexes were found to be enriched in disruptive mutations (Purcell et al., 2014), de novo copy-number variants (CNVs) (Kirov et al., 2012), non-synonymous de novo single-nucleotide variants (SNVs), and small insertions or deletions (indels) (Fromer et al., 2014) in schizophrenia cases. ARC protein has been described to accumulate at synapses in Angelman syndrome (Greer et al., 2010) and increased and/or decreased in several animal models of Alzheimer’s disease and patient-derived cells (for a review, see Kerrigan and Randall, 2013). These data suggest that Arc is a component of protein complexes that are involved with human cognitive disorders.

In this paper, we have conducted an extensive proteomic and genetic dissection of Arc protein complexes, which is a generic strategy suitable for the characterization of potentially any synaptic protein. We have focused on the following four challenges: isolation of native multiprotein complexes from brain tissue; visualization of the endogenous protein using genetic tagging; genetic dissection of protein complex organization and localization using mouse genetic models; and genetic dissection of complexes using human genetic data, including human disease and cognitive phenotypes. Here we demonstrate that this integrated proteomic and genetic strategy reveals insights into the physiological functions of Arc and the synaptic basis of mental disorders and intelligence.

## Results

### TAP Tagging and Proteomic Analysis of Endogenous Arc Complexes

**Figure 1:**
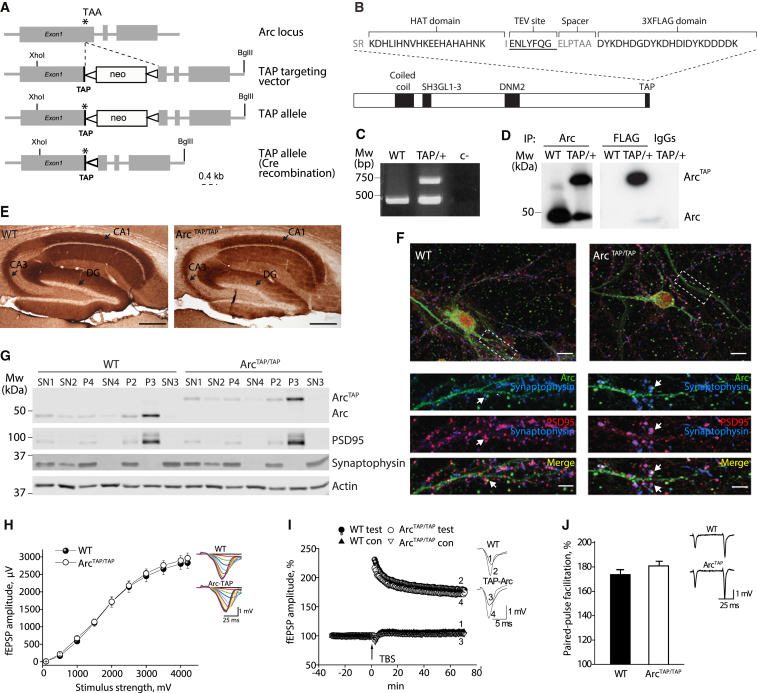
Generation of TAP-Tagged Arc Knockin Mice (A) Scheme of the genomic Arc locus targeted with the TAP tag. The TAP sequence was inserted before the stop codon of the protein. The cross of Arc^TAP^ knockin mice with a transgenic Cre-expressing mouse line deleted the neomycin (neo) resistance cassette by recombination between loxP sites. Asterisk, stop codon (TAA) of the coding sequence; thick black line, TAP tag sequence; triangle, loxP site. (B) Structure of the TAP-tagged Arc regions, including a potential coiled-coil domain, an SH3-endophilin-2 and -3 binding region, a dynamin-2 binding region, and the C-terminal TAP tag sequence domain before the stop codon of the protein. (C) PCR amplification of WT (bottom band) and TAP Arc-targeted alleles (top band). (D) TAP-tagged Arc was specifically purified from Arc^TAP/+^ forebrain extracts with anti-Arc and anti-FLAG antibodies and blotted with an anti-Arc antibody. (E) Hippocampal sections of WT and Arc^TAP/TAP^ mice stained with an anti-Arc antibody. DG, dentate gyrus. Scale bar, 1 mm. Shown is a representative image of n = 2 mice for each genotype. (F) Representative image of embryonic primary neurons derived from WT and Arc^TAP/TAP^ mouse independent cultures immunostained at day in vitro (DIV) 15 with antibodies against Arc (green), PSD-95 (red), and Synaptophysin (blue). Merged, colocalization of the three signals. Arrows show the punctum labeling for each protein. Scale bar, 10 μm. (G) Biochemical fractionation from Arc^TAP/TAP^ and WT mouse forebrains. Similar protein amounts from each fraction were loaded onto a gel and immunoblotted with the antibodies displayed at the right. TAP-tagged Arc showed the same subcellular distribution as the WT non-tagged isoform. The fractions are described in the Supplemental Experimental Procedures. Antibodies against synaptophysin were used as a specific marker of the SN3 fraction. Actin was used as a loading control. MW, molecular weight in kilodaltons; TAP/+, heterozygous for TAP-tagged Arc; c-, PCR water; IgG, mouse total immunoglobulin G. (H) Basal synaptic transmission was normal in Arc^TAP/TAP^ mice. Areas under input-output curves were not statistically different in Arc^TAP/TAP^ (n = 15, N = 5) and WT animals (n = 18, N = 5) (F_(1,7.06)_ = 0.258; p = 0.627). (I) Normalized magnitude of the LTP 60–65 min after LTP induction did not differ in mutant mice (166% ± 5%; n = 15, N = 5; F_(1,7.75)_ = 0.449; p = 0.522) relative to their WT counterparts (171% ± 4%; n = 18, N = 5). (J) Paired-pulse facilitation was not statistically different (F_(1,7.36)_ = 2.405; p = 0.163) in Arc^TAP/TAP^ animals (n = 15, N = 5) compared with their WT littermates (n = 18, N = 5). Data are presented as mean ± SEM, with n = slices and N = mice.

**Figure 2:**
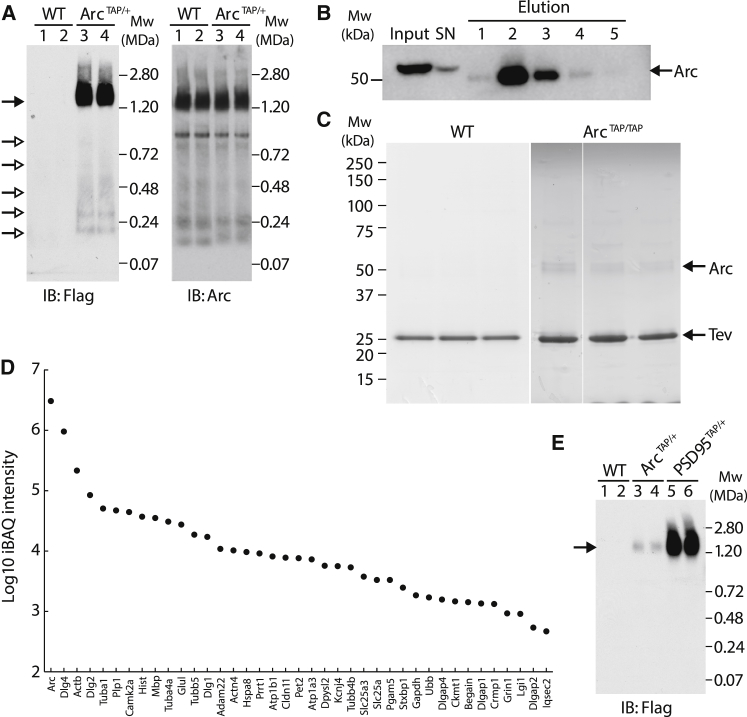
TAP of Arc Reveals Postsynaptic Complexes with a Native Size of 1.5 MDa (A) BNP of WT and Arc^TAP/+^ forebrain extracts blotted with FLAG and Arc antibodies, where Arc and TAP-tagged Arc can be mainly detected at 1.5 MDa. The closed arrow indicates the main Arc complex, whereas open arrows indicate lower-molecular-weight Arc complexes. (B) Arc was tandem affinity-purified from Arc^TAP/TAP^ forebrain extracts, eluted, and collected in 5 consecutive fractions following histidines affinity tag (HAT) purification. (C) Colloidal Coomassie staining of three independent TAPs from WT (left) and Arc^TAP/TAP^ (right) forebrains. The lanes were cut for LC-MS/MS, and the identified proteins are listed in Table S1. Arc and the Tev enzyme are indicated. (D) The absolute expression value of each protein in the tandem purification was estimated by the iBAQ intensity values obtained in each purification. (E) BNP of WT, Arc^TAP/+^, and PSD95^TAP/+^ forebrain extracts blotted with FLAG antibody. TAP-tagged Arc and TAP-tagged PSD95 levels are detected. TAP/+, heterozygous for TAP-tagged Arc or PSD95 as indicated; SN, supernatant.

To label and isolate endogenous Arc protein, we engineered knockin mice (Arc^TAP^) harboring a TAP tag fused to the C terminus of Arc (Figures 1A–1D). Mice carrying the TAP tag showed no detectable alterations in the levels or localization of Arc in the brain or in hippocampal synaptic physiology (Figures 1E–1J). Native Arc complexes were detected by immunoblotting of brain extracts separated on blue native PAGE (BNP), which showed a major band of a median mass of ∼1.5 MDa, with several additional minor species ranging from ∼200–700 kDa (Figure 2A).

The TAP tag was used to isolate Arc complexes directly from mouse forebrain tissue using a highly efficient purification protocol (recovering >70% Arc) (Figures 2B and 2C), and their composition was determined using liquid chromatography-tandem mass spectrometry (LC-MS/MS). The single-step purification yielded 107 high-confidence proteins, whereas the more stringent tandem purification protocol recovered a subset of 39 proteins (34 of 39 were uncovered by single-step purification) (Experimental Procedures; Table S1; http://www.genes2cognition.org/publications/tap-arc). Eight of 14 previously reported Arc interactors were found among the 107 high-confidence proteins, indicating that 99 were novel interactors (Supplemental Experimental Procedures). Among the 107 high-confidence proteins, 72 proteins contain the Arc-N lobe consensus motif P[STVILMKR][FYH] (Zhang et al., 2015), revealing a strong network of direct interactors (Table S1). Comparisons of mouse and human show that 87% (92 of 107) of Arc-interacting proteins were conserved between species (Table S2), 70% (1,012 in human and 1,447 in mice) of protein-protein interactions were conserved (Table S3), and the Arc interactome was enriched (72%) in proteins in the human postsynaptic complexes found by Bayés et al. (2012) (Table S3). Together, these results suggest that we have defined a robust Arc complex and interactome that is highly conserved between mouse and human.

PSD95 was the most abundant Arc-interacting protein. Using intensity-based absolute quantification (iBAQ) quantification (Schwanhäusser et al., 2011) of the single-step purification, it showed ∼1:1 stoichiometry with Arc (Figure 2D; Table S4), and in the tandem-purification, it represented 57% of the Arc interactome (Table S4). Reciprocal immunoprecipitations show that PSD95 assembles into Arc complexes from early developmental stages (post-natal day 11 [P11]) in the hippocampus and cortex (Figures S1A and S1B). The Dlg family of adaptor/scaffold proteins, comprising four paralogs (SAP97/*Dlg1*, PSD93/*Dlg2*, SAP102/*Dlg3*, and PSD95/*Dlg4*), was the most abundant of eleven protein classes recovered, suggesting that they play a principal role in regulating Arc function (Figure S1C; Tables S5 and S6). Specificity of interaction between Arc and Dlg paralogs was suggested by the finding that PSD93 and SAP97 were also highly abundant, whereas SAP102 was not detected in the Arc interactome (confirmed using reciprocal immunoprecipitation; Figure S1D). Forty-nine percent of Arc-interacting proteins were known PSD95 interactors and particularly enriched in membrane proteins, including NMDA and AMPA receptors (Table S1).

Consistent with their co-assembly with Arc, the NMDA receptor, PSD95, and PSD93 were also shown to reside in 1.5-MDa supercomplexes (Frank et al., 2016). To compare the relative abundance of Arc and PSD95 in 1.5-MDa supercomplexes, we immunoblotted Arc^TAP/+^ and PSD95^TAP/+^ brain extracts separated by BNP with FLAG antibodies (Figure 2E). PSD95 was ∼20-fold more abundant than Arc, indicating that ∼5% of PSD95 complexes contain Arc. Pull-down of PSD95 complexes using the TAP tag recovered 65% of PSD95 and depleted 37% of Arc, indicating that ∼58% of Arc is in PSD95 supercomplexes (data not shown). Together, these data indicate that PSD95 is the major interacting protein of Arc and that a subset of the postsynaptic 1.5-MDa PSD95 supercomplexes contain Arc.

### Arc Postsynaptic Localization Requires PSD95

**Figure 3:**
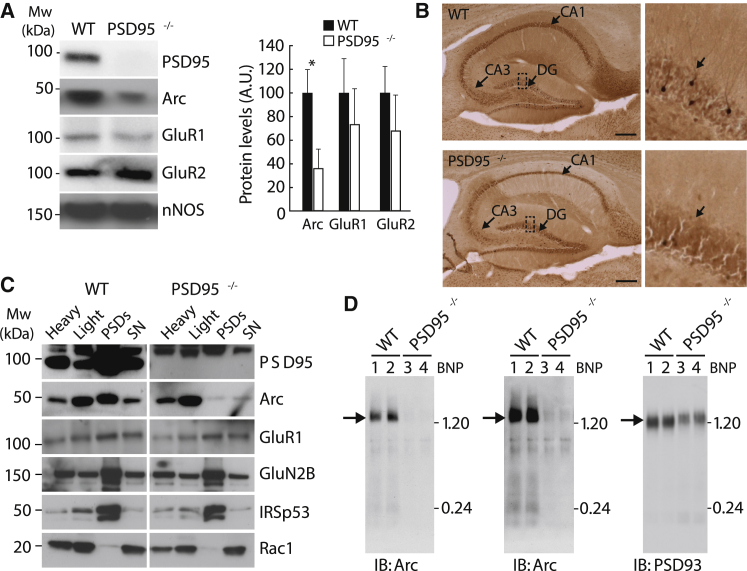
Arc Protein Levels Are Reduced in Fractions of PSD95 Knockout Mice (A) Representative immunoblot showing the relative abundance of PSD95, Arc, GluR1, and GluR2 proteins in total hippocampal lysates from PSD95^−/−^ and matched WT littermates. Arc is reduced to 35.0% ± 17.1% of the WT in the PSD95 mutant mice (N = 4 for each matched pair, ^∗^p < 0.05). Neuronal NOS (nNOS) was used as a loading control. (B) Representative Arc staining of sagittal sections of the hippocampus (left) and magnification of the granular layer (right) for WT and PSD95 knockout mice. Scale bar, 1 mm. (C) Hippocampal extracts of PSD95 mutant and WT mutant mice were biochemically fractionated into synaptosomes and into cytoskeletal and vesicular components, referred to as “light.” The synaptosomal fraction was subsequently dissociated into PSDs and Triton X-100 soluble fraction. Arc levels were dramatically reduced in the PSD95 mutant, whereas no changes in GluR1, GluN2B, IRSp53, and Rac1 proteins were observed. (D) Blue native PAGE (BNP) of WT and PSD95 knockout forebrain extracts blotted with Arc and PSD95 antibodies. Long exposure of the blots shows Arc complexes migrating at a lower molecular weight than 1.20 MDa (center). SN, Triton X-100 soluble fraction.

How Arc is localized to the postsynaptic terminal is unknown. To address this question, we asked whether members of the Dlg scaffold protein family were required in vivo, using mice carrying knockout mutations in PSD95, PSD93, and SAP102 (SAP97 knockout mice are nonviable). In hippocampal extracts, we found that Arc protein levels were reduced (35.0% ± 17.1% of the wild-type [WT], p < 0.01) in PSD95 knockout mice but not in PSD93 or SAP102 knockout mice (Figure 3A; Figures S1E and S1F). A dramatic loss of dendritic staining of Arc was observed in hippocampal sections from PSD95 knockout mice (Figure 3B). Furthermore, synaptosomes from PSD95 knockout mice also showed a major reduction in Arc (Figure 3C). We also examined BNP immunoblots from PSD95 knockouts and found that 1.5-MDa Arc complexes were severely diminished, with a weak residual signal after long exposure of the gel (Figure 3D). Thus, PSD95 is specifically required to localize Arc to the postsynaptic terminal.

**Figure 4:**
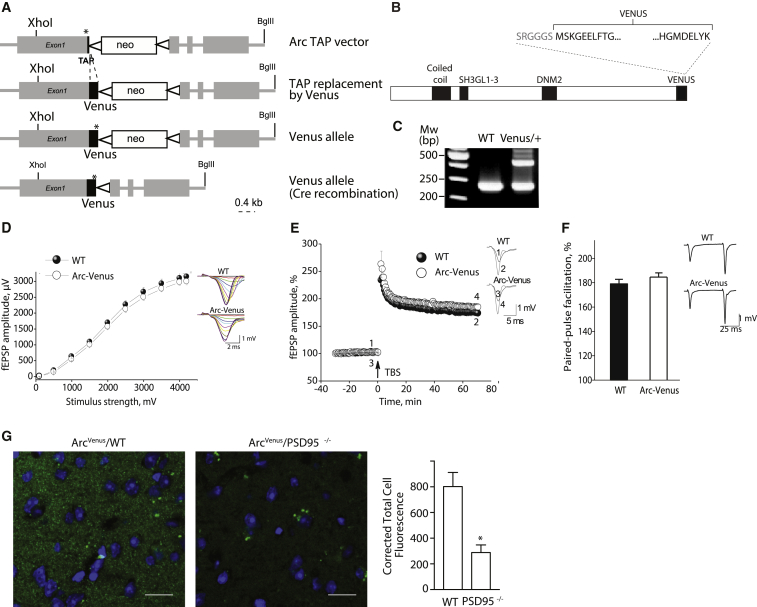
Generation of Venus-Tagged Arc Knockin Mice (A) The Venus sequence was inserted before the stop codon of the protein using the TAP vector as template. The cross of both knockin mice with a transgenic Cre-expressing mouse line deleted the neo resistance cassette by recombination between loxP-sites. Asterisk, stop codon (TAA) of the coding sequence; thick black line, TAP and Venus tag sequence, as indicated; triangle, loxP site. (B) Structure of the Venus-tagged Arc regions as in Figure 1B. (C) PCR amplification of WT (bottom band) and Arc Venus-targeted alleles (top band). Venus/+, heterozygous for Venus-tagged Arc. (D) Input-output relationships illustrate averaged field excitatory postsynaptic potential (fEPSP) amplitudes in slices from Arc^Venus/Venus^ (n = 26, N = 8) and WT mice (n = 22, N = 8) in response to stimulation of Schaffer collaterals. Areas under input-output curves were not significantly different between genotypes (F_(1,13.06)_ = 0.499; p = 0.493). (E) Normalized magnitude of LTP 60–65 min after LTP induction did not differ significantly in mutant mice (185% ± 4%; n = 25, N = 8; F_(1,11.64)_ = 2.92; p = 0.114) relatively to their WT counterparts (174% ± 4%; n = 22, N = 8). (F) Paired-pulse facilitation was not statistically different (F_(1,11.08)_ = 1.372, p = 0.266) in Arc^Venus/Venus^ animals (n = 26, N = 8) compared with their WT littermates (n = 22, N = 8). Data are presented as mean ± SEM, with n = slices and N = mice. (G) Representative section of Arc^Venus^ mouse brain crossed with WT (left) and PSD95^−/−^ (right) mice. Shown is a bar chart of the total cell fluorescence corrected by the area and the background signal. Scale bars, 15 μm.

To visualize endogenous Arc protein, we created Arc^Venus^ knockin mice using a similar design strategy as for the Arc^TAP^ mice, where the Venus fluorescent protein was fused to the C terminus of Arc (Figures 4A–4C). Mice carrying the Venus tag showed no detectable alterations in hippocampal synaptic physiology (Figures 4D–4F). We bred Arc-Venus mice with PSD95 knockouts to generate compound transgenic mice (Arc^Venus^xPSD95^−/−^) and asked whether kainic acid-induced neuronal activity (Li et al., 2005) would drive Arc to the synapse and whether this required PSD95. In the absence of PSD95, Arc-Venus failed to localize to the synapse (Figure 4G). These results demonstrate that PSD95 is required for the postsynaptic localization of Arc into 1.5-MDa complexes in the steady state and following induction by neuronal activity.

### Proteomic Analysis of Arc Complexes in Mice Lacking PSD95

**Figure 5:**
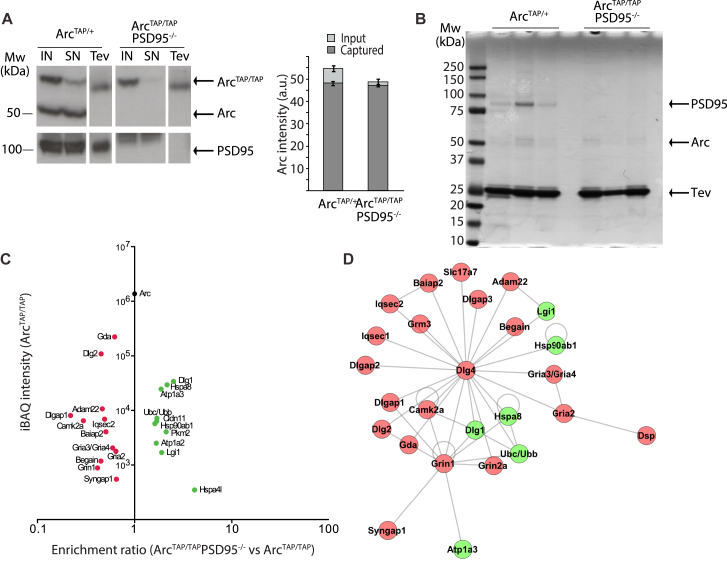
Quantitative Proteomics Analysis of Arc^TAP^ Reveals a Depletion of Postsynaptic Proteins in PSD95 Knockout Mice (A) Arc complexes were isolated from Arc^TAP/+^ and Arc^TAP/TAP^ mice crossed with PSD95 knockout mice (Arc^TAP/TAP^xPSD95^−/−^) by FLAG capture and Tev protease release (single-step purification). Total lysate (IN, input) and the same volume of lysate upon purification (SN) and Tev elution from both genotypes were blotted against Arc and quantified. Eluted Arc levels following the FLAG capture from Arc^TAP/+^ and Arc^TAP/TAP^xPSD95^−/−^ lysates were not statistically different (Mann-Whitney *U* test, p = 0.1). Data are presented as mean ± SEM. (B) Isolated complexes from (A) were resolved by SDS-PAGE and stained with colloidal Coomassie. Three independent purifications are shown. The lanes were cut for LC-MS/MS analysis, and the identified proteins are listed in Table S7. TAP-tagged Arc, PSD95, and the Tev enzyme are indicated. (C) Dimethyl labeling-based quantitative MS of TAP-purified proteins from Arc^TAP/+^ and Arc^TAP/TAP^ crossed with PSD95 knockout mouse forebrain (Arc^TAP/TAP^×PSD95^−/−^). The plot displays enrichment ratios of Arc^TAP/TAP^×PSD95^−/−^ versus Arc^TAP/+^ (x axis) and iBAQ enrichment values of the step purification (y axis). Proteins meeting criteria for enrichment (>1.5 fold) are highlighted in green and for depletion (< 0.667 fold) are highlighted in red. The names of depleted and enriched PSD95 interactors are indicated. See the Supplemental Experimental Procedures for enrichment criteria. (D) Mouse interactome network constructed from the publicly available databases BioGrid, Database of Interacting Proteins (DIP), IntAct, Molecular INTeraction Database (MINT), STRING database, UniProt, Biomolecular Interaction Network Database (BIND) and mentha using the Psicquic software package. The network is visualized using Visone. Proteins highlighted in green/red meet the enrichment/depletion criteria discussed in the Supplemental Experimental Procedures.

We reasoned that, by genetic removal of PSD95, we could identify those Arc-interacting proteins that were most dependent on PSD95. We bred Arc^TAP/TAP^ with PSD95 knockout mice (Arc^TAP/TAP^/PSD95^−/−^) and analyzed their Arc interactome using quantitative proteomic methods (Figures 5A–5C; Table S7). As shown in Figure 5C, Arc-interacting proteins separated into two broad subgroups: depleted and enriched proteins (see red and green proteins, respectively). Seventy percent of depleted proteins were PSD95-interacting proteins, including PSD93. As shown by immunoblots of BNPs, PSD93 remained in 1.5-MDa complexes in PSD95^−/−^ mice (Figure 3D; Frank et al., 2016). Absence of PSD93 did not affect the interaction of Arc with PSD95 (Figure S1G). The most significant gene ontology (GO) biological process (BP) terms in the depleted proteome were synaptic transmission (p = 1.23 × 10^−11^), cell-cell signaling (p = 3.94 × 10^−9^), and modulation of synaptic transmission (p = 1.35 × 10^−7^), highlighting the functional importance of the depleted proteins (Table S6).

Among the 12 most enriched proteins in the Arc complexes isolated from PSD95 mutant mice were SAP97 and structural proteins, including those with a potential role in cell growth and adhesion (Claudin11 and Lgi1). A network graph of the interactions of the enriched and depleted protein sets is shown in Figure 5D. The internal network consists of 26 proteins and 44 interactions (visualized using Visone; Brandes and Wagner, 2012). Taken together, these proteomic and in vivo genetic studies show that Arc is tethered to postsynaptic 1.5-MDa signaling complexes containing PSD95, and when these complexes are abolished in PSD95 mutants, Arc is found associated with cytoskeletal and structurally related proteins. Thus, Arc is partitioned into either the PSD95 supercomplexes in the postsynaptic terminal or into cytoskeletal complexes.

### Arc Complexes in Disease

Proteins within the postsynaptic proteome are assembled into complexes and supercomplexes (Frank et al., 2016), and this supramolecular organization is of crucial importance in human genetic disorders because it is a mechanism by which the many different gene products functionally converge. We therefore combined our proteomic datasets with human genetic datasets to understand the importance of Arc-interacting proteins in human disease.

Preliminary proteomic data from Arc^TAP^ mice was previously used to implicate the disruption of ARC complexes in human psychiatric disorders (Fromer et al., 2014, Kirov et al., 2012, Purcell et al., 2014). The first such study revealed that components of Arc complexes were enriched in de novo CNVs from individuals with schizophrenia (Kirov et al., 2012), with subsequent studies finding enrichment for rare point mutations in individuals with schizophrenia, autism, and intellectual disability (ID). Here we extend preliminary proteomic data from Arc^TAP^ mice using complete sets of Arc-interacting proteins and additional genetic datasets, including epilepsy and healthy control de novo datasets.

We first sought to replicate the initial finding of Kirov et al. (2012) with the comprehensive Arc interactome. Utilizing the same genetic dataset as Kirov et al. (2012), we found that de novo CNVs from schizophrenia probands were enriched for Arc complex genes compared with de novo CNVs from unaffected individuals (p = 0.0047). Arc interactors whose association with Arc is depleted in PSD95 knockout mice largely drove this enrichment (p = 0.0165), indicating the importance of the postsynaptic 1.5-MDa complexes. We next investigated enrichment of the Arc interactome for rare point mutations and indels contributing to brain disorders, using exome sequencing data from a case/control schizophrenia study (Purcell et al., 2014) and de novo studies performed in cohorts of schizophrenia, autism, ID, and epilepsy (Supplemental Experimental Procedures). Combining evidence from each of these independent datasets, we found strong support for the enrichment of both nonsynonymous (NS) and loss-of-function (LoF) disease-related mutations among Arc interactors (p = 9.01 × 10^−12^ and 2.051 × 10^−7^, respectively; Table 1). All five datasets contributed to this enrichment (Table 1; Tables S8 and S9), indicating that disruption of Arc complexes may contribute to a wide range of brain disorders. Consistent with the analysis of de novo CNVs, much of the enrichment in LoF and NS mutations was attributable to Arc interactors whose expression is altered in the PSD95^−/−^ mouse. This suggests that it is postsynaptic Arc-PSD95 complexes and not cytoplasmic Arc complexes that are relevant to these disorders.

**Table 1:** Arc Gene Set Analysis of Autism, Schizophrenia, Epilepsy, ID, and Schizophrenia Candidate Gene Sets

			All Arc Interactors		Arc Interactors with Increased Expression in PSD95/*Dlg4*^−/−^ Mice		Arc Interactors with Decreased Expression in PSD95/*Dlg4*^−/−^ Mice		Arc Interactors that Are Known Direct PSD95-Interacting Proteins	
			N = 107		N = 11		N = 24		N = 18	

**Mutation Class**	**Disease**	**Study Design**	**P**	**O/E**	**P**	**O/E**	**P**	**O/E**	**P**	**O/E**

CNVs	schizophrenia	de novo	0.00469	–	0.50211	–	0.01648	–	0.01648	–
LoF	combined	–	2.05E-07	–	1.00000	–	1.57E−06	–	3.03E−06	–
	autism	de novo	0.14352	11/4.0	1.00000	0/0.4	0.10920	6/1.2	0.00104	8/0.8
	epilepsy	de novo	0.39935	3/0.4	1.00000	0/0	1.00000	1/0.1	1.00000	0/0.1
	ID	de novo	0.00104	8/0.5	1.00000	0/0	0.00104	5/0.1	0.00936	3/0.1
	schizophrenia	de novo	1.00000	3/0.8	1.00000	1/0.1	1.00000	2/0.2	0.60527	2/0.2
	schizophrenia	case/control	1.00000	56/38	1.00000	12/9	1.00000	15/9	1.00000	6/6
NS	combined	–	9.01E−12	–	1.00000	–	1.59E−06	–	1.26E−08	–
	autism	de novo	0.01352	46/25.9	1.00000	2/2.5	1.00000	15/8.5	0.00416	18/5.8
	epilepsy	de novo	0.00104	16/2.5	1.00000	0/0.3	1.00000	3/0.8	1.00000	2/0.6
	ID	de novo	0.00104	15/2.0	1.00000	0/0.2	0.00104	8/0.6	0.00104	8/0.4
	schizophrenia	de novo	0.11856	14/5.7	1.00000	3/0.6	0.58759	6/1.8	0.47839	5/1.3
	schizophrenia	case/control	1.00000	187/184	1.00000	22/21	1.00000	61/48	0.72800	40/31

Shown are enrichment test empirical p values for autism (De Rubeis et al., 2014, Iossifov et al., 2012, Jiang et al., 2013), epilepsy (EuroEPINOMICS-RES Consortium et al., 2014), ID (de Ligt et al., 2012, Hamdan et al., 2014, Rauch et al., 2012), and schizophrenia (Fromer et al., 2014, Girard et al., 2011, Gulsuner et al., 2013, Kirov et al., 2012, McCarthy et al., 2014, Xu et al., 2012). For de novo studies, O/E indicates the observed versus expected (under a null model) number of mutations in that class. For the schizophrenia (SCZ) case/control study, O/E indicates the number of case versus control mutations. P indicates Bonferroni multiple-test correction: four tests for CNVs, 12 tests for Lof/NS combined analyses, and 52 tests for LoF/NS in individual studies. Fisher’s method was used to combine p values from the five independent genetic datasets (“combined” p value for LoF and NS mutations). PSD95^−/−^, PSD95/*Dlg4* knockout mice.

### Arc Complexes in Normal Variation in Human Intelligence

Although the role in cognition for Arc, PSD95, and their interacting proteins is well established from studies of mutations in mice (Fernández et al., 2009, Fitzgerald et al., 2014, Husi et al., 2000, Komiyama et al., 2002, McCurry et al., 2010, Migaud et al., 1998, Nithianantharajah et al., 2013, Plath et al., 2006, Ryan et al., 2013), mutations in humans cause cognitive disorders, and enrichment analysis of Arc-interacting proteins for mammalian phenotype (MP) terms shows 48 enriched terms (p < 0.01) associated with abnormal synaptic and cognitive functions (Table S10), much less is known about the relevance to normal variation in human cognition. We therefore asked whether common genetic variation in Arc complexes was associated with common variation in general cognitive ability (known as intelligence or *g*) using the genome-wide association study (GWAS) on intelligence from the five cohorts (n = 3,511) that make up the Cognitive Aging in England and Scotland (CAGES) consortium (Davies et al., 2011, Hill et al., 2014). The five cohorts are the Lothian Birth Cohort of 1921 and 1936 (Deary et al., 2012), the Aberdeen Birth Cohort of 1936 (Whalley et al., 2011), and the Manchester and Newcastle Longitudinal Studies of Cognitive Aging (Rabbitt et al., 2004), which together consist of a total of 3,511 healthy middle- to old-aged individuals who all live independently in the community. The measure of general cognitive ability was taken from the GWAS previously conducted by Hill et al. (2014) (Supplemental Experimental Procedures). To determine whether there was a greater weight of evidence for association between the Arc gene set and general cognitive ability, a two-stage enrichment test was used. First, SNPs were assigned to autosomal genes, and a gene based statistic was derived (Liu et al., 2010). Second, the p values of the gene-based statistics were –log(10)-transformed before gene set enrichment analysis (GSEA) (Subramanian et al., 2005) and a competitive test of enrichment, was used. The results of the gene-based analysis are shown in Table S11, where eight genes were nominally significant in CAGES and nine in the Brisbane Adolescent Twin Study (BATS). The most significant gene in the BATS cohort (*PRRT1*, p = 0.00732) was also nominally significant in CAGES (p = 0.03797). The results of the enrichment analysis show that common genetic variation in Arc complex proteins shows nominally significant association (p = 0.0473) with intelligence compared with control gene sets. A replication study using the summary data of a GWAS conducted on intelligence (Hill et al., 2014), the BATS (n = 2,062; de Zubicaray et al., 2008, Wright et al., 2001, Wright and Martin, 2004), also showed a significant enrichment (p = 0.0247), confirming the results found in the CAGES consortium. This significant enrichment shows that common genetic variation in the genes encoding Arc complex proteins is associated with the normal variation in human intelligence differences.

## Discussion

We have developed and demonstrated an integrated proteomic and genetic strategy that reveals insights into Arc’s role in biology, the synaptic basis of mental disorders, and intelligence. Multiple genetic and genome engineering methods were combined to isolate native Arc complexes, identify their constituents, determine the mechanism of assembly and localization to the postsynaptic terminal, and identify multiple diseases and mutations that converge on the complexes.

The Arc protein is principally housed within 1.5-MDa complexes, and proteomic MS identified many novel Arc-interacting proteins, of which PSD95 was the most abundant. PSD95 and Arc coassemble into 1.5-MDa supercomplexes, and knockout of PSD95 abolishes these complexes, severely depletes Arc from the postsynaptic terminal, and prevents its activity-dependent recruitment. The combined use of gene-tagged and mutant mice allowed us to dissect the interactions of Arc with specific subsets of postsynaptic complexes. PSD95 supercomplexes are a family of which ∼3% contain NMDA receptors (Frank et al., 2017). The NMDA receptor requires PSD93 for coassembly with PSD95 (Frank et al., 2016), and in the present study, we found that PSD93 knockouts did not interfere with Arc-PSD95 interactions. Therefore, Arc can assemble with PSD95 supercomplexes that do not contain NMDA receptors. We also found that Arc did not interact with SAP102, which forms distinct complexes at ∼350 kDa (Frank et al., 2016), nor did Arc require SAP102 for postsynaptic targeting. Together, these results demonstrate that Arc is targeted to the postsynaptic terminal, where it selectively interacts with signaling complexes organized by PSD95. Super-resolution microscopy has revealed that PSD95 and SAP102 are in separate nanodomains (Zheng et al., 2011) within the dendritic spine and that PSD95 nanodomains (Broadhead et al., 2016, Fukata et al., 2013, Nair et al., 2013) are positioned beneath the presynaptic release machinery (Tang et al., 2016). This suggests that Arc is selectively targeted by PSD95 to this critical region of the postsynaptic terminal, where its supercomplexes participate in controlling synaptic transmission and plasticity.

Disruption of many proteins in Arc-PSD95 complexes, and many other proteins in the supercomplexes leads to changes in synaptic plasticity and behavior, including knockout of Arc and PSD95, which both lead to enhanced LTP and impaired learning (Carlisle et al., 2008, Migaud et al., 1998, Nithianantharajah et al., 2013, Plath et al., 2006; N.H.K., L.N. van de Lagemaat, L.E. Stanford, C.M. Pettit, D.J. Strathdee, K.E. Strathdee, D.G.F., E.J. Tuck, K.A.E., T.J. Ryan, J.N., N.G. Skene, M.D.R.C., and S.G.N.G., unpublished data; M.V.K., L.N. van de Lagemaat, N. Afinowi, D.J. Strathdee, K.E. Strathdee, D.G.F., E.J. Tuck, K.A.E., N.G. Skene, M.D.R.C., N.H.K., and S.G.N.G., unpublished data). A recent large-scale genetic screen of postsynaptic proteins in mice showed that PSD95 supercomplexes were essential for the postsynaptic responses to simple and complex patterns of activity and the modulation of synaptic strength over a range of milliseconds to an hour (M.V.K., L.N. van de Lagemaat, N. Afinowi, D.J. Strathdee, K.E. Strathdee, D.G.F., E.J. Tuck, K.A.E., N.G. Skene, M.D.R.C., N.H.K., and S.G.N.G., unpublished data). The supercomplexes were also essential for tuning the magnitude of innate and learned behavioral responses, including simple and complex forms of behavior (N.H.K., L.N. van de Lagemaat, L.E. Stanford, C.M. Pettit, D.J. Strathdee, K.E. Strathdee, D.G.F., E.J. Tuck, K.A.E., T.J. Ryan, J.N., N.G. Skene, M.D.R.C., and S.G.N.G., unpublished data). Furthermore, these studies show that each innate and learned behavioral response required a specific subset or combination of postsynaptic proteins, which suggests that transient upregulation and targeting of Arc to PSD95 supercomplexes will transiently modify behavior and synaptic physiology. This mechanism is consistent with the known role of Arc in learning.

The proteomes of the post-synaptic density (PSD) and PSD95 supercomplexes are highly conserved between mice and humans (Bayés et al., 2011), and specific genes (e.g., PSD93) have conserved roles in cognition (visuo-spatial learning, cognitive flexibility, and attention) (Nithianantharajah et al., 2013). Our finding that human genetic disorders of cognition converge on Arc-PSD95 supercomplexes is in agreement with the mouse genetic findings. Here we have reaffirmed the role of the supercomplexes in schizophrenia and extended the study to autism and ID. Moreover, the finding that variation in normal human intelligence and disorders of cognition involves the same sets of proteins indicates that genetic variation in Arc-PSD95 supercomplexes underpins the phenotypic continuum between normal cognitive variation and pathology.

There are over 130 brain diseases linked to mutations in the postsynaptic proteome (Bayés et al., 2011) and a large number of uncharacterized multiprotein complexes (Frank et al., 2016), many of which contain at least one protein encoded by a disease gene. The integrated workflow shown here, which is centered on genetically tagged mice and proteomic approaches, offers a general and scalable approach toward understanding how the polygenic basis of brain disease is linked to the supramolecular organization of proteins in the postsynaptic terminal of central synapses. All datasets are freely available through the Genes to Cognition website (http://www.genes2cognition.org).

## Experimental Procedures

### Animals

All animal experiments were conducted in a licensed animal facility in accordance to guidelines determined by the UK Animals (Scientific Procedures) Act, 1986 and approved through the U.K. Home Office Inspectorate. Animal care at KU Leuven was conducted according to national and international guidelines and as described in the Supplemental Experimental Procedures. All mice were 2- to 5-month-old males unless indicated otherwise.

### TAP

TAP was performed as by Fernández et al. (2009). Briefly, mouse forebrain was homogenized on ice in 1% deoxycholate (DOC) buffer (50 mM Tris [pH 9.0], 1% sodium deoxycholate, 50 mM NaF, 20 μM ZnCl_2_, and 1 mM Na_3_VO_4_), 2 mM Pefabloc SC (Roche), and 1 tablet/10 mL protease inhibitor cocktail tablets (Roche) at 0.38 g wet weight per 7 mL cold buffer with a glass Teflon Douncer homogenizer. The homogenate was incubated for 1 hr at 4°C and clarified at 50,000 × *g* for 30 min at 4°C. TAP-tagged complexes were isolated as described previously (Fernández et al., 2009). The SDS-PAGE gel was fixed and stained with colloidal Coomassie, and lanes were cut into slices, destained, and digested overnight with trypsin (Roche, trypsin modified, sequencing grade) as described previously (Fernández et al., 2009). Peptide digestion, LC-MS/MS, and proteomics data analysis are described in the Supplemental Experimental Procedures.

### Enrichment Analysis of CNVs and Rare Coding Mutations in Arc Interactors in Human Neuropsychiatric Disease

The protein IDs from Table S1 were converted into both mouse genome informatics (MGI) and mouse NCBI/Entrez gene IDs using the online ID mapping tool provided by Uniprot and then converted to human Entrez IDs using the mapping file “HOM_MouseHumanSequence.rpt,” available from MGI (http://www.informatics.jax.org/). Any genes with a non-unique (e.g., 1-many) mapping between species, or where MGI and mouse Entrez IDs mapped to different human genes, were excluded. De novo CNV enrichment analysis and de novo mutation exome sequencing datasets are detailed in the Supplemental Experimental Procedures.

### Human Cognitive Ability Phenotype and Analysis

The phenotypes used in both the CAGES and the BATS samples were taken from the summary data of Hill et al. (2014) and are described in the Supplemental Experimental Procedures. Genome-wide association had been carried out in each cohort of CAGES using Mach2QTL (Li et al., 2010) before being meta-analyzed in METAL (Willer et al., 2010) and is detailed in the Supplemental Experimental Procedures.

All other methods are described in the Supplemental Experimental Procedures.

## Author Contributions

Conceptualization, E.F. and S.G.N.G.; Methodology, E.F., F.Z., and N.H.K.; Investigation, E.F., M.O.C., R.A.W.F., F.Z., J.N., S.A.L., and M.V.K.; Validation, E.F.; Disease Genetics, A.J.P., M.F., and S.M.P.; Cognition Genetics, W.D.H. and I.J.D.; Resources, J.S.C. and C.B.; Data Curation, M.D.R.C., C.M., and J.D.A.; Technical Assistance, D.F., K.A.E., C.L.M., and G.C.; Writing – Original Draft, E.F. and S.G.N.G.; Writing – Review & Editing, E.F., M.O.C., R.A.W.F., M.V.K., J.N., A.J.P., N.H.K., J.S.C., C.B., and S.G.N.G.; Project Management, Coordination, and Funding Acquisition, S.G.N.G.

## Data and Software Availability

The accession number for the mass spectrometry proteomics data reported in this paper is ProteomeXchange Consortium: PXD007283.

## Supplemental Information

Document S1. Supplemental Experimental Procedures and Figure S1

Table S1. MS Data Obtained from a Single-Step and Tandem Affinity Purifications, Related to Figure 2

Table S2. The Human Arc Proteome, Related to Figure 2

Table S3. The Mouse and Human Arc Interactome, Related to Figure 2

Table S4. iBAQ Intensity Numbers of the ARC Core Interactors, Related to Figure 2

Table S5. Classification of 107 Arc Interactors According to Their Protein Class, Related to Figure S1

Table S6. Biological Process Ontology of 107 Arc Interactors, Related to Figure 3 and Table S7

Table S7. MS Data Obtained from Single-Step Purifications from Arc^TAP/+^ and Arc^TAP/TAP^ Crossed with PSD95/Dlg4 Knockout Mice, Related to Figures 3 and 5

Table S8. Arc Gene Set Enrichment in Schizophrenia, Epilepsy, Autism, and Intellectual Disability, Related to Table 1

Table S9. Analysis of Silent Mutations, Related to Table 1

Table S10. Mammalian Phenotype Ontology of Arc Interactors, Related to Table 1 and Table S1

Table S11. Arc Gene Set Association with General Cognitive Ability, Related to Figure 2 and Table S1

Document S2. Article plus Supplemental Information
