# Supplementary material for: Arc Requires PSD95 for Assembly into Postsynaptic Complexes Involved with Neural Dysfunction and Intelligence
Source: Cell Rep. 2017 Oct 17;21(3):679–91. doi: 10.1016/j.celrep.2017.09.045 (PMC5656750; doi:10.1016/j.celrep.2017.09.045)
Supplement: Document S1. Supplemental Experimental Procedures and Figure S1 [file mmc1.pdf]

Cell Reports, Volume *21*

Supplemental Information

Arc Requires PSD95 for Assembly into Postsynaptic Complexes Involved with Neural Dysfunction and Intelligence

Esperanza Fernández, Mark O. Collins, René A.W. Frank, Fei Zhu, Maksym V. Kopanitsa, Jess Nithianantharajah, Sarah A. Lemprière, David Fricker, Kathryn A. Elsegood, Catherine L. McLaughlin, Mike D.R. Croning, Colin Mclean, J. Douglas Armstrong, W. David Hill, Ian J. Deary, Giulia Cencelli, Claudia Bagni, Menachem Fromer, Shaun M. Purcell, Andrew J. Pocklington, Jyoti S. Choudhary, Noboru H. Komiyama, and Seth G.N. Grant

Supplemental Figure 1

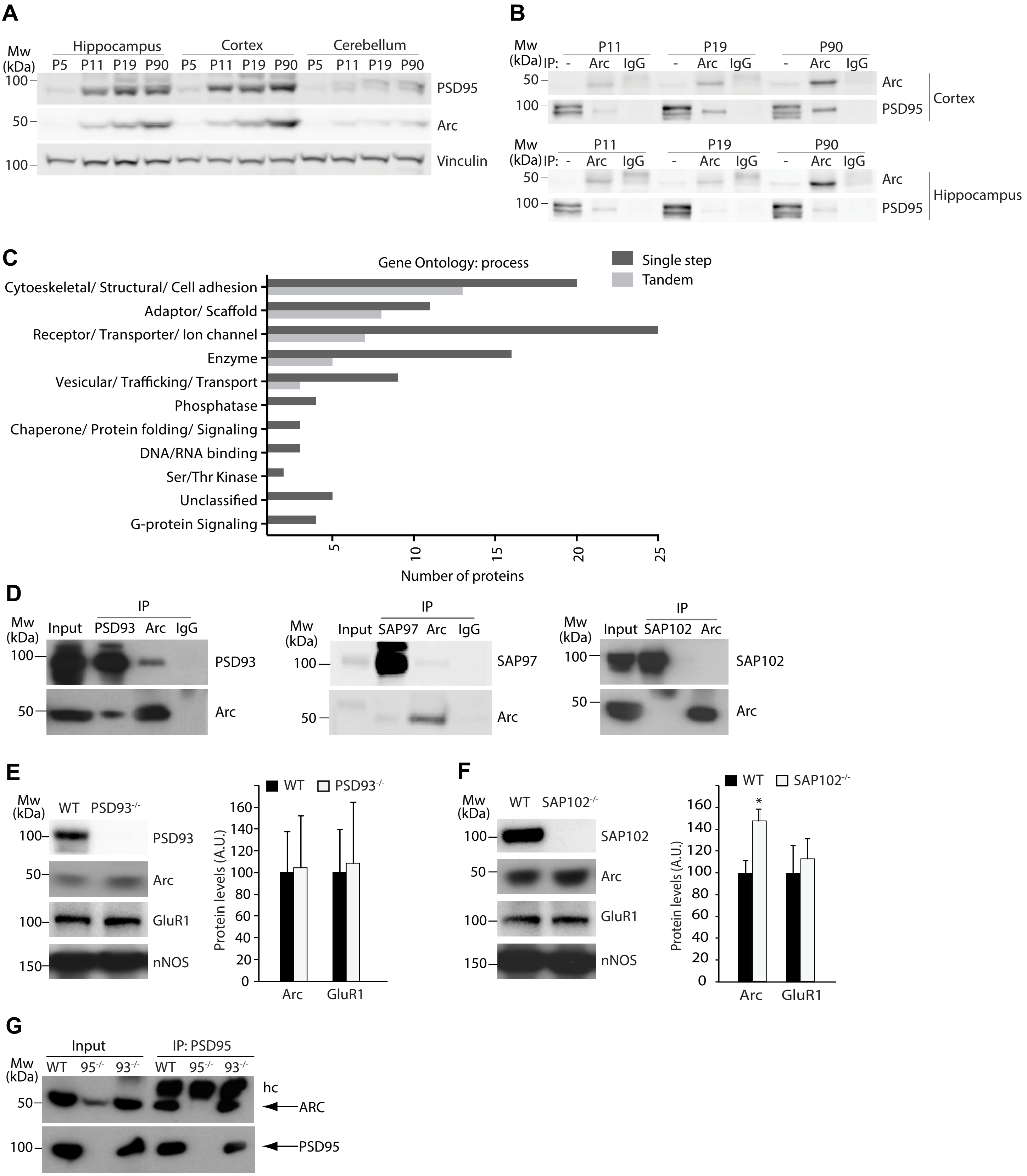

Figure S1. Selective interaction of MAGUK proteins with Arc complexes, Related to Figure 2. (A) PSD95 and Arc expression in hippocampus, cortex and cerebellum from wild type mice at postnatal days 5, 11, 19 and 90 (P5, P11, P19 and P90, respectively). Vinculin was used as loading control. **(B)** Arc was specifically purified with an antibody anti-Arc from hippocampal and cortical extracts at the developmental stages indicated. Immunoprecipitated complexes were blotted against Arc and PSD95. (**C**) Bar diagram with the number of proteins purified in the single step and the tandem purifications according to their gene ontology identifier. (**D**) Arc and the MAGUKs PSD93, PSD97 and SAP102 were purified with specific antibodies from forebrain WT extracts and blotted against Arc and PSD93 (lef panel), PSD97 (middle panel) and SAP102 (right panel) antibodies. IgG, mouse total IgGs; Mw: molecular weight in kDa. **(E)** and **(F)** Representative immunoblots of N = 4 independent animals, showing relative abundance of different proteins in lysates of hippocampus from PSD93 (**E**) and SAP102 (**F**) knockout mice and matched wild type littermates. Western blots were probed with antibodies against SAP102, PSD93, Arc, GluR1, and nNOS as indicated. Quantification of protein expression was calculated to the amount of nNOS in each sample and expressed as a percentage of the WT expression. N= 4 for each genotype. * $P< 0.05$, Mann-Whitney *U* test. (**G**) Arc interaction to PSD95 is independent of PSD93. PSD95 was immunoprecipitated from PSD95 and PSD93 mutant and WT mice forebrain extracts and immunoblotted against Arc and PSD95. Representative blot of n = 3 independent experiments. IP: antibodies used for immunoprecipitation, hc: Antibody heavy chain. -/-: knockout mice.

Supplemental Experimental Procedures

1. Materials

Sodium deoxycholate, 3-[(3-cholamidopropyl)dimethylammonio]-1- propanesulfonate, n-Dodecyl-N,N-Dimethyl-3-Ammonio-1-Propanesulfonat, β-D-maltopyranoside, N,N'-bis-(3-D-gluconamidopropyl) deoxycholamide (Sigma); anti-Arc (Santa Cruz Biotechnologies; C7); anti-Arc (Santa Cruz Biotechnologies; H300), anti-PSD95 (Thermo Scientific; 6G6-1C9), anti-Synaptophysin (EMD Millipore; 573822), anti-GluR1 (EMD Millipore; AB1504), anti-Dynamin-3, (BD-Biosciences; 610246), anti-actin (Sigma; A5228), anti-nNOS (EMD Millipore; AB1632), anti-SAP102 (NeuroMab; 75058), anti-PSD93 (NeuroMab; 75-057), anti-SAP97 (BD Biosciences; 610874/5), secondary antibodies conjugated with Alexa Fluor dyes (Invitrogen), biotinylated anti-mouse secondary antibody (1:200; DAKO; E0413) and peroxidase-linked secondary IgGs (EMD Millipore; 12-348 and -349).

2. Animals

Animal care at KU Leuven was conducted according to national and international guidelines (Belgian law of August 14th, 1986, and the following K.B. of November 14th, 1993 and K.B of September 13th, 2004; European Community Council Directive 86/609, OJa L 358, 1, December 12, 1987; National Institutes of Health Guide for the Care and Use of Laboratory Animals, National Research Council, 1996). Animals were exposed to conventional 12:12h light/dark cycles and ad libitum food supply. Mice were sacrificed by cervical dislocation and the forebrain was dissected on ice and snap-frozen in liquid nitrogen. Brain samples were stored at -80 ºC for few weeks prior to use.

3. Generation of TAP and Venus tagged Arc vectors and gene targeting.

The TAP tag fragment containing the Histidine Affinity Tag (HAT), TEV protease and Flag sequences flanked by XbaI and BclI restriction sites was cloned into pneoflox vector (TAPtag pneoflox) as described previously (Fernandez et al., 2009). Two homology arms of the genomic Arc/Arg3.1 sequence were amplified with forward ArcHAXhoIF and reverse ArcHAXbaIR primers and forward ArcHAAcc65IF and reverse ArcHABglIIR primers using the BAC bMQ101B9 as a template. Both homology arms were cloned into the TAPtag pneoflox vector leaving in between the TAP tag sequence, 2 loxP sites, PGK and EM7 promoters, the G418^r gene and an SV40 polyadenylation site. To generate the Venus cassette, the coding sequence of the yellow fluorescent protein (YFP) variant Venus was amplified by PCR with an artificial linker encoding for Gly-Gly-Gly-Ser at its 5' end with the primers VenusF and VenusR. Both *pneoflox-ArcTap* vector and Venus PCR products were digested with XbaI and BclI and Venus directly ligated to the vector, resulting on *pneoflox-ArcHA12-Venus* vector.

The cassettes of both targeting vectors flanked by two homology arms were removed and transformed into *EL350 E. coli* cells containing a pTargeter vector with the genomic Arc/Arg3.1 sequence cr1574496136 to cr15 74506490 (Ensembl release 49). The cassettes were inserted into the pTargeter vector by recombination (Liu et al., 2003). The final vectors contained a 5' -end homolog Arc sequence of 6664bp and a 3' -end homology arm of 3688bp (ENSMUSG00000022602). The vectors were linearized with PvuI and electroporated into E14 embryonic stem cells. The neomycin resistant colonies were picked up, expanded and frozen. Genomic DNA was extracted from all of them and PCR with and pneoF3 and Arc4R to identify TAP Arc homologous recombinants and with pneoF3 and Arc4R to identify Venus Arc recombinants.

4. Arc^{TAP} and Arc^{Venus} transgenic mice generation

One of the ES cells positive clones for each recombinant was microinjected into C57BL/6 blastocysts and 4 germline chimaeras were generated containing 30-90% of targeted cells. These chimaeras were crossed onto the C57BL/6 genetic background. Tail DNA from the litters was extracted and analyzed by PCR with a 5' Arc1F forward primer and two 3' pneo3R and Arc10R reverse primers to distinguish the heterozygous (+/-) and wild type (+/+) TAP Arc alleles, respectively, and with the primers NeoF1 and ArcExonF2 forward primers and ArcUTRR2 reverse primer to distinguish the heterozygous (+/-) and wild type (+/+) Venus alleles, respectively. Both mouse strains were bred with Cre-recombinase expressing transgenic mice to remove the Neo^r cassette. The knockin strains are referred as Arc^{TAP} and Arc-Venus. Both strains did not show any distortion of transmission frequency in the offspring of the intercrosses. TAP tagged Arc protein levels were similar to wild type Arc levels in forebrain extracts of Arc$^{TAP/+}$ mice and TAP tagged Arc was specifically immunoprecipitated by specific anti-Arc and anti-FLAG antibodies (Figure 1D).

5. Proteomics

Peptides were extracted from gel bands twice with 50% acetonitrile/0.5% formic acid and dried in a SpeedVac (Thermo). Peptides were resuspended using 0.5% formic acid analyzed using an Ultimate 3000 Nano/Capillary LC System (Dionex) coupled to either an LTQ FT Ultra (for characterization of TAP tagged Arc complexes) or an LTQ Orbitrap Velos (differential analysis of TAP tagged Arc complexes in WT or PSD95 mutants) hybrid mass spectrometers (Thermo Electron) equipped with a nanospray ion source. Peptides were desalted on-line using a micro-Precolumn cartridge (C18 Pepmap 100, LC Packings) and then separated using either a 35 min RP gradient (4-32% acetonitrile/0.1% formic acid) on a BEH C18 analytical column (1.7 μm, 75 μm id x 10 cm,) (Waters) (for characterization of TAP tagged Arc complexes) or a 125 min RP gradient (4-32% acetonitrile/0.1% formic acid) on an Acclaim PepMap100 C18 analytical column (3 μm, 75 μm id x 50 cm,) (Dionex) (differential analysis of TAP tagged Arc complexes in WT or PSD95 mutants). The mass spectrometer was operated in standard data dependent acquisition mode controlled by Xcalibur 2.0/2.1. The LTQ-FT Ultra was operated with a cycle of one MS (in

the FTICR cell) acquired at a resolution of 100,000 at m/z 400, with the top five most abundant multiply-charged (2+ and higher) ions in a given chromatographic window subjected to MS/MS fragmentation in the linear ion trap. The LTQ-Orbitrap Velos was operated with a cycle of one MS (in the Orbitrap) acquired at a resolution of 60,000 at m/z 400, with the top 20 most abundant multiply-charged (2+ and higher) ions in a given chromatographic window subjected to MS/MS fragmentation in the linear ion trap. FTMS target values of 5e5 (LTQ-FT Ultra) and 1e6 (LTQ-Orbitrap Velos) and an ion trap MSn target values of 1e4 were used. Maximum FTMS scan accumulation times were set at 1000ms (LTQ-FT Ultra) and 500ms (LTQ-Orbitrap Velos) and maximum ion trap MSn scan accumulation tiles were set to 200ms (LTQ-FT Ultra) and 100ms (LTQ-Orbitrap Velos). Dynamic exclusion was enabled with a repeat duration of 45s with an exclusion list of 500 and exclusion duration of 30s.

6. Proteomic data analysis
Data was analyzed data using MaxQuant version 1.3.0.5 (Cox and Mann, 2008). MaxQuant processed data was searched against a UniProt mouse proteome sequence database (Mar, 2012) using the following search parameters: trypsin with a maximum of 2 missed cleavages, 7 ppm for MS mass tolerance, 0.5 Da for MS/MS mass tolerance, with Acetyl (Protein N-term) and Oxidation (M) set as variable modifications and carbamidomethyl (C) as a fixed modification. A protein FDR of 0.01 and a peptide FDR of 0.01 were used for identification level cut offs. Protein quantification was performed using razor and unique peptides and using only unmodified and carbamidomethylated peptides.

Summary of one-step purifications
Two sets of one-step purifications:
3 replicates of ARC$^{TAP/TAP}$ and WT (as control) purifications dimethyl labelled for quantification by MaxQuant).
2 replicates of ARC$^{TAP/TAP}$ and 1 control (MaxQuant intensity-based label free quantification)
Proteins were considered enriched if they had a ratio of enrichment of 2.5 (ARC$^{TAP/TAP}$/Control purification) in two out of 5 one-step purifications.

Summary of tandem purifications
Three replicates of ARC$^{TAP/TAP}$ and control purifications (MaxQuant intensity-based label free quantification). Proteins were considered enriched if they had a ratio of enrichment of 2.5 (ARC$^{TAP/TAP}$/Control purification) in two out of three two-step purifications. The total set of 107 ARC complex components was assembled from the combined set of enriched proteins from one step and tandem purifications.

Summary of purification from ARC$^{TAP/TAP}$/PSD95$^{-/-}$ mice
Arc binding partners were captured from lysed forebrain from Arc$^{TAP/TAP}$xPSD95$^{-/-}$. In order to isolate similar amounts of Arc-associated protein complexes from WT and PSD95$^{-/-}$, TAP tagged Arc was captured from heterozygous Arc$^{TAP/+}$ mice. The TAP procedure, concentration of the eluted samples and gel staining were performed as described in Experimental procedures. The amount of protein lysed, captured and eluted was monitored by semiquantitative immunoblotting before LC-MS/MS (Figure 5A).
Three replicate one-step purifications of ARC complexes from ARC$^{TAP/+}$, ARC$^{TAP/TAP}$/PSD95$^{-/-}$ and control mice were dimethyl labelled for quantification by MaxQuant. Given that ARC expression is down-regulated in PSD-95 mutant mice, all protein ratios were normalized to that determined for ARC by MaxQuant. 77 out of the total set of 107 ARC complex proteins were quantified in dimethyl labelled PSD95 mutant versus wild type experiments and 59 of these proteins could be quantified in at least 2 out of 3 replicates. Of these, 24 were decreased by 1.5-fold in the PSD-95 mutant (Ratio < 0.67) and 11 showed a 1.5 fold increase in abundance in the PSD95 mutant (Ratio >1.5).

7. Blue Native PAGE
Blue-native PAGE has been performed as described in (Frank et al., 2016). Briefly, forebrains (hippocampus and cortex) were dissected from adult (P56-70) mice and were homogenized (12 strokes with a Teflon-glass pestle and mortar) in 5 ml ice-cold buffer H (1 mM Na HEPES pH7.4, 320 mM sucrose with protease inhibitors). The homogenate pellet was collected by centrifugation with 1,000 x*g*. at 2°C for 10 min was re-homogenized (6 strokes) in 2 ml buffer H and centrifuged as before. The first and second 1000 x*g*. supernatants were pooled and centrifuged at 18500 x*g*. to pellet the crude membranes. The 18500 x*g*. supernatant was discarded. Extraction conditions were screened using the crude membrane from 50-60 mg mouse forebrain re-suspended in 0.5 ml buffer H, to which different detergents sodium deoxycholate, 3-[(3-cholamidopropyl)dimethylammonio]-1-propanesulfonate, n-Dodecyl-N,N-Dimethyl-3-Ammonio-1-Propanesulfonat, β-D-maltopyranoside, N,N'-bis-(3-D-gluconamidopropyl) deoxycholamide in 0.5 ml buffer X (100-300 mM NaCl, 50 mM tris.Cl pH8) were mixed for 1 h at 6-10°C. Insoluble proteins were removed from the total extract by centrifugation at 120,000 x*g*. for 40 min at 8°C. BNPs were immediately run and immunoblotted to detect the native complexes of Arc, PSD95 and PSD93.

8. Cellular fractionation
Brain cellular fractionation was done according to the protocol described earlier (Chowdhury et al., 2006). Mouse forebrains were homogenized in 10 volumes of sucrose buffer (0.32 M sucrose, 4 mM HEPES, pH 7.4, 1 mM EDTA, 1 mM EGTA and protease inhibitors cocktail tablets (Roche)) with a glass Teflon Dounce homogenizer, and centrifuged at 800 g for 15 min. The

supernatant (SN1) was centrifuged 15 min at 9000 g and the pellet collected as crude membrane fraction (P2). The supernatant (SN2) containing the cytosolic fraction was again centrifuged for 60 min at 100,000g. The pellet was collected as microsomal fraction (P4) and the supernatant (SN4) was considered as the cytoskeletal fraction. P2 crude membrane fraction containing synaptosomes was solubilized in 0.2% DOC buffer in the presence of protease inhibitors and centrifuged for 20 min at 100,000g. Pellet (P3) was considered as PSD-enriched fraction and supernatant (SN3) was considered as presynaptic fraction. Fractions were stained with antibodies against PSD95 (Thermo Scientific; 6G6-1C9), Arc (Santa Cruz Biotechnologies; H300), Synaptophysin (EMD Millipore; 573822), and actin (Sigma; A5228).

9. Immunostaining of brain sections and primary neurons

TAP tagged Arc showed a similar distribution pattern to wild type Arc (Steward et al., 1998) in dendrites of hippocampal CA1, CA3 areas and dentate gyrus (Figure 1E) Mice were anesthetized with a mix of ketamine/xylazine and intracardially perfused with 0.1 M sodium phosphate buffer (pH 7.4) followed by 4 % paraformaldehyde in 0.1M sodium phosphate buffer (pH 7.4). The brain was removed and placed in a fixative for a further 1 h and then equilibrated overnight in 30 % sucrose in PBS. Free-floating sections were incubated with anti-Arc (1:250; Santa Cruz Biotechnologies; C7) and biotinylated anti-mouse secondary antibody (1:200; DAKO; E0413) for peroxidase staining. The signal was amplified using an avidin-biotin system (Vector Laboratories) followed by a staining in 3,3-diaminobenzidine (Sigma).

TAP tagged Arc showed a similar subcellular distribution as wild type Arc being partly co-localized with PSD95 and synaptophysin in synaptic boutons. Hippocampi were dissected from E15.5 mouse embryos of wild type (WT) and $Arc^{TAP/TAP}$ C57Bl/6 littermates and digested in papain to yield suspension of primary neurons (Figure 1F). The culture conditions and the immnocytochemistry protocol were performed as described earlier (Valor et al., 2007). Primary neurons were fixed and stained at 14 days *in vitro* (DIV) and labeled with the following antibodies: rabbit Arc 1/250 (Santa Cruz Biotechnologies; H300), IgG2A mouse PSD95 1/350 (Thermo Scientific; 6G6-1C9) and IgG1 mouse Synaptophysin 1/1000 (EMD Millipore; 573822). Secondary antibodies conjugated with Alexa Fluor dyes (Invitrogen) were applied against the specific IgGs. All images were taken on a Zeiss 510 META confocal microscope using a 63x Plan-Apochromat objective. Z-stacks were taken at 0.8 □m intervals and maximal projections were made to give the images showed.

10. Kainic stimulation of Arc-Venus and WT mice.

Briefly, animals were injected intraperitoneal with a saline solution of kainic acid 20mg/kg at a volume of 10ml/kg and allowed to rest in a quiet dimly-lit room during 2 hours. Mice were then anesthetised by intraperitoneal injection of 0.1 mL of 20% pentobarbital sodium (Euthatal, Merial animal health Ltd.) and placed back in their cage until complete anaesthesia. When no responses were observed after pinching toes and tail, two tail samples were collected for PCR genotyping. Then the mouse was pinned down on a cork surface and the thorax was open. Once the heart was fully exposed, a small incision was performed in the right atrium and a needle was immediately inserted in the left ventricle for transcardiac injection of 10 mL of phosphate buffered saline (PBS, Fisher Scientific) followed by 10 mL of 4% paraformaldehyde (PFA, Alfa Aesar 16%, diluted 1:4 in PBS). Next, the brain was dissected out and post-fixed in 4% PFA at 4°C for 3-4h. Brains were always kept in the dark from the moment of dissection to preserve the fluorescence of the fusion proteins. The brain was then cryo-protected by incubation in 30% sucrose (VWR Chemicals, w/v in PBS) at 4°C for 72h. Brains were then embedded in optimal cutting temperature (OCT) medium (VWR international) inside a mould (Sigma-Aldrich). They were immediately frozen by placing the mould in a plastic beaker containing isopentane (Sigma-Aldrich), placed itself in liquid nitrogen. Once frozen, brains were stored at -80°C. Coronal sections (18 µm thickness) were cut with a cryostat (NX70 Thermo Fisher) and directly mounted on Superfrost Plus glass slides (Thermo scientific). Mounted sections were then left to dry at room temperature overnight and stored at -80°C the next day. Sections were stained with DAPI (Sigma, 1:10000. Mowiol was used to mount brain samples in high-refractive index mounting medium.

11. Electrophysiology in hippocampal slices

Previous studies show that disruption of Arc/Arg3.1 led to an impairment of synaptic long-term potentiation (LTP) and long-term depression (LTD) in the hippocampal CA1 area and dentate gyrus (Guzowski et al., 2000; Messaoudi et al., 2007; Shepherd et al., 2006). In view of these findings, we sought to explore if incorporation of the TAP and Venus tags into Arc led to disturbances of synaptic transmission and plasticity. Field excitatory postsynaptic potentials (fEPSPs) in the CA1 area of acute hippocampal slices were recorded using multi-electrode arrays as previously described (Coba et al., 2012; Kopanitsa et al., 2006). Baseline stimulation strength was adjusted to evoke a response that corresponded to ~40% of the maximal attainable fEPSP at the recording electrode located in proximal stratum radiatum. Amplitude of the negative part of fEPSPs was used as a measure of the synaptic strength. The effect of the mutation on the basal synaptic transmission was estimated by comparing areas under input-output curves (AUC_{I-O}) measured in individual slices. Paired stimulation with an interpulse interval of 50 ms was used to observe paired-pulse facilitation (PPF) in baseline conditions in the test pathway before LTP induction. PPF was calculated by dividing the amplitude of the fEPSP obtained in response to the second pulse by the amplitude of fEPSP evoked by the preceding pulse. To induce LTP, 10 bursts of baseline strength stimuli were administered at 5 Hz to test pathway with 4 pulses given at 100 Hz per burst (total 40 stimuli). LTP plots were scaled to the average of the first five baseline points. Normalization of LTP values was performed by dividing the fEPSP amplitude in the tetanized pathway by the amplitude of the control fEPSP at corresponding time points. Normalized LTP values averaged across the period of 61–65 min after theta-burst stimulation were used for statistical comparison.

12. Forebrain extracts and western blot

Forebrain extracts were solubilized in 1% DOC buffer at 0.38 g wet weight per 7 ml cold buffer with a glass Teflon Douncer homogenizer. The homogenate was incubated for 1 h at 4ºC and clarified at 50,000 *g* for 30 min at 4ºC. Protein concentration was quantified with the Bradford protein assay (BioRad). Samples containing equal amount of protein were subjected to reducing SDS electrophoresis (NUPAGE, Invitrogen) and transferred to polyvinyldifluoride membrane (Hybond™-P, GE Healthcare). The membranes were blocked in 5% non-fat milk, 0.01% Triton X-100 in PBS with the following antibodies: mouse Arc (Santa Cruz Biotechnology; C7), mouse SAP102 (NeuroMab; 75058), mouse PSD93 (NeuroMab; 75-057), mouse SAP97 (BD Biosciences; 610874/5), rabbit GluR1 (EMD Millipore; AB1504), and nNos (EMD Millipore; AB1632). Detection of signals was carried out using peroxidase-linked secondary IgGs (EMD Millipore; 12-348 and -349) and enhanced chemiluminescence (GE Healthcare). Quantification of the signals was performed using ImageJ open source program.

13. MAGUKs immunoprecipitation

Based on the LC-MS/MS data on the tandem purification of Arc complexes (Table S1), we verified the interaction of Arc with the MAGUKs PSD95 and checked whether the interaction of PSD95 and Arc was affected by the absence of PSD93. Mouse forebrains of the genotype indicated were homogenized in 1% DOC buffer following the same protocol as in the aforementioned TAP procedure. Equal amount of protein extracts was incubated for 2 h at 4 ºC with a mouse anti-Arc, -PSD95, -PSD93, -SAP97 or -SAP102. Captured complexes were then washed three times with DOC buffer and eluted with 1x NuPAGE LDS sample buffer (Invitrogen) supplemented with a reducing agent. Absence of PSD93 did not affect the interaction of Arc with PSD95 suggesting that PSD-95-Arc interaction is independent of PSD93.

14. Human and mouse Arc protein-protein interactions (PPIs).

We obtained mouse and human PPI interactions for the core 107 Arc genes from Table S1, using the publicly available databases: BioGrid (Chatr-Aryamontri et al., 2013),DIP (Salwinski et al., 2004), IntAct (Orchard et al., 2014),, MINT (Orchard et al., 2014), STRING (Jensen et al., 2009), UniProt (UniProt, 2014), BIND (Bader et al., 2003), mentha (Calderone et al., 2013) , APID (Alonso-Lopez et al., 2016) and HPRD (Prasad et al., 2009) using the Psicquic package (Aranda et al., 2011). In total we found 1447 reported mouse interactions, 1012 of these (~70%) were found in human.

15. Classification of Arc interactors according to their gene ontology process.

We performed enrichment analysis of the core 107 Arc genes from Table S1, for GO Biological Process (BP) terms using the topGO package (Alexa et al., 2006). The genome wide annotation package "org.Mm.eg.db" (Marc Carlson, org.Mm.eg.db: Genome wide annotation for Mouse. R package version 3.2.3) was used to obtain a background annotation set, leading to a background set of 23313 Entrez gene identifiers mapped onto 10623 BP terms.

P-values were calculated using the Exact Fisher test, and corrected for multiple hypothesis testing using the Benjamini Hochberg (BH) correction. In Table S6 we found the 107 Arc set enriched for 'ion transport', 'cation transport' and 'Synaptic transmission' (P=8.2x10-14, P=5.2x10-13 and P=5.7x10-13 respectively). These terms remained enriched in the down-regulated Arc subset from Table S7 (P=1.x10-3, P=9.2x10-3 and P=9.7x10-14 respectively). The upregulated subset from Table S7 was not found enriched for these terms. Instead this subset was found enriched for 'cellular potassium homeostasis', 'ATP metabolic process' and 'potassium ion homeostasis' (P=3.0x10-5, P=6.4x10-5 and 6.9x10-5 respectively). All the tables of the manuscript are compiled in http://wwwdev.genes2cognition.org/publications/tap-arc/.

16. Creation of human Arc interactor genesets

From the original 107 Arc interactor mouse proteins, we identified homologous human genes based on the Mouse Genome Informatics database (http://www.informatics.jax.org/faq/ORTH_dload.shtml). After mapping and manual curation of gene identifiers, we found 124 human Arc interactors RefSeq genes, of which 13 were associated with Arc with increased values in the PSD95$^{-/-}$ mouse, 37 of which were associated with Arc with decreased values, and 18 of which were known direct interactors of PSD95 (Table S7).

17. Mammalian Phenotype Ontology of Arc interactors

We performed enrichment analysis of the core 107 Arc genes from Table S1, for Mammalian Phenotype (MP) terms using the Ontology package topOnto (https://github.com/statbio/topOnto). MGI's 'All Genotypes and Mammalian Phenotype' annotation file (ftp://ftp.informatics.jax.org/pub/reports/MGI_PhenoGenoMP.rpt) was used to obtain a background annotation set, leading to a background set of 8770 Entrez gene identifiers mapped onto 8813 MP terms.

P-values were calculated using the Exact Fisher test, and corrected for multiple hypothesis testing using the Bonferroni correction (at the 0.01 '***' and 0.05 '**' significance levels), and the Benjamini Hochberg (BH) correction. In Table S10 we found the 107 Arc set enriched for 'abnormal synaptic transmission', 'abnormal CNS synaptic transmission', 'abnormal nervous system physiology' (P=4.4x10-16, P=7.8x10-15 and 9.5x10-15 respectively). These terms remained enriched using the down-regulated Arc subset from Table S7 (P=7.7x10-12, P=1.4x10-12 and P=1.3x10-10 respectively). The upregulated subset from Table S7 was not found enriched for these terms.

18. Network building
Interactions were mined from publicly available databases: BioGrid (Chatr-Aryamontri et al., 2013), DIP (Salwinski et al., 2004), , (Orchard et al., 2014), MINT (Orchard et al., 2014)STRING (Jensen et al., 2009), UniProt (2014), BIND (Bader et al., 2003), mentha (Calderone et al., 2013) using the Psicquic software package (Aranda et al., 2011). The network is visualised using Visone (http://visone.info/html/).

19. Human genetic analysis
***De novo* CNV enrichment analysis**
For each gene set, the number of genes hit by 34 *de novo* CNVs from schizophrenia case were compared those hit by 59 *de novo* CNVs from unaffected Icelandic controls. A gene was counted as being hit by a CNV if the CNV overlapped any part of its length (human genome Build 36.3). To overcome biases related to gene and CNV size, and to control for differences between studies and genotyping chips, the following logistic regression models were fitted to the combined set of CNVs:
(a) logit (pr(case)) = CNV size + total number of genes hit
(b) logit (pr(case)) = CNV size + total number of genes hit + number of genes hit in gene set

Comparing the change in deviance between models (a) and (b), a one-sided test for an excess of genes in the gene set being hit by case CNVs was performed. The genetic data used for this analysis is described in (Kirov et al., 2012). Note that while a small number of the protein groups from Table S1 correspond to multiple proteins/genes, their presence does not influence the analysis as none were hit by either case or control CNVs.

***De novo* mutation exome sequencing datasets**

**Disease**	***N* trios**	***N* LoF mutations**	***N* nonsynonymous mutations**	**Sources (PMIDs)**
Autism	3,985	579	3446	25363760 23849776 25363768
Epilepsy	356	58	341	25262651
ID	192	67	259	23033978 25356899 23020937
Schizophrenia	1024	114	756	24463507 21743468 23911319 24776741 23042115

The Swedish schizophrenia case/control association data were based on summary statistics from a previously described dataset (Purcell et al., 2014). We used PLINK/Seq (http://atgu.mgh.harvard.edu/plinkseq/) to uniformly re-annotate all mutations with respect to RefSeq genes. Consistent with the previously reported work, for all *de novo* studies we focused on two classes of mutation: 1) gene disruptive (or loss-of-function, LoF) mutations (i.e. nonsense, essential splice site or frameshift indels); 2) a broad class of all nonsynonymous mutations (including disruptive mutations). For the case/control schizophrenia study, we focused on 1) rare gene disruptive mutations with a sample frequency of less than 0.1%; 2) rare (<0.1%) nonsynonymous mutations that were predicted *in silico* to have a likely deleterious effect on protein function, following (Purcell et al., 2014). Analysis of silent mutations was included as control (Table S9). For the analysis of *de novo* mutations, we used DNENRICH, a freely-available software previously described (Fromer et al., 2014) (https://psychgen.u.hpc.mssm.edu/dnenrich/). Briefly, DNENRICH estimates the observed number of *de novo* mutations per gene or geneset by a genomic permutation strategy, which controls for gene size and structure, sequence coverage and local trinucleotide mutation rate. Significance of the enrichment in the rate and recurrence of mutations was assessed empirically by permutation. For the case/control data, we used the SMP algorithm (Purcell et al., 2014) which is part of the PLINK/Seq package (http://atgu.mgh.harvard.edu/plinkseq/). Briefly, the test for enrichment of a set of genes is based on the sum of gene-level case/control burden statistics relative to the exome-wide excess of burden in cases. Significance is assessed by permutation, comparing the observed distribution against 10,000 null replicates (created by shuffling case/control labels within groups matched for ancestry and technical variables).

Neuropsychiatric genetic analyses: multiple testing correction.
We first sought to verify the initial finding of Arc complex enrichment in de novo CNVs that has formed the foundation for all subsequent studies; this we correct for the 4 Arc complex gene-sets tested. We then turned to the analysis of rare coding variants. While there is consistent evidence for disease-variant enrichment across multiple independent studies (based on preliminary Arc data), these associations are modest. We therefore combine enrichment p-values from each rare-variant dataset to provide an overall summary of the evidence. These summary p-values are corrected for 12 tests: 4 Arc complex gene-sets tested x 2 classes of mutation (LoF and NS), plus the 4 de novo CNV tests already performed. This correction is likely to be conservative, as LoF and NS mutations are not independent sets. Finally, we present enrichment p-values for individual datasets, correcting for 52 tests (4 Arc complex gene-sets x LoF and NS mutations x 5 individual datasets, plus the 12 tests already performed).

20. Human cognitive ability phenotype

In the case of the CAGES consortium a general factor of cognitive ability (*g* or intelligence) was derived separately in each cohort. For the Lothian Birth Cohort of 1921 (LBC1921) consists of 550 individuals (females = 316) with a mean age of 79.1 years (SD = 0.6) (Deary et al., 2009; Deary et al., 2004; Scottish Council for Research in Education, 1933). The tests used to derive a measure of intelligence were the Moray House Test (MHT) (Scottish Council for Research in Education, 1933), Raven's Standard Progressive Matrices (Raven et al., 1977), the Wechsler Logical Memory scores (Wechsler, 1987), phonemic verbal fluency (Lezak et al., 2004), and the National Adult Reading Test (NART) (Nelson and Willison, 1991). The Lothian Birth Cohort of 1936 (LBC1936) includes data on 1091 individuals (females = 543) with a mean age of 69.5 years (SD = 0.8) (Deary et al., 2007). The tests used to measure intelligence in the LBC1936 were the Digit Symbol Coding, Block Design, Matrix Reasoning, Digit Span Backwards, Symbol Search and the Letter Number Sequencing from the Wechsler Adult Intelligence Scale (Wechsler, 1998) and the NART (Nelson and Willison, 1991). The Aberdeen Birth Cohort of 1936 (ABC1936) has data on 498 (females=255) older individuals (mean age = 64.6, SD = 0.9 years of age). In order to provide a measure of intelligence the following tests were used. The Rey Auditory Verbal Learning Test (Lezak et al., 2004), the Uses of Common objects Test (Guildford et al., 1978), Raven's Standard Progressive Matrices (Raven et al., 1977) and Digit Symbol (Wechsler, 1981) along with the NART (Nelson and Willison, 1991). In each of these three cohorts intelligence was measured by subjecting the raw scores from the tests used to a principal components analysis within each cohort. The first unrotated component was extracted using regression and was used as the dependent variable in a linear regression with age and sex being used as predictors. The standardised variables from this model were then used in the subsequent analysis (Hill et al., 2014a). In the Manchester and Newcastle cohorts consist of 1563 individuals (female = 1108) with a median age of 65 ranging from 44 – 93 years of age. The tests used were the four parts of the Culture Fair Test (Cattell and Cattell, 1960) and the two parts of the Alice Heim Test 4 (Heim, 1970) were used. Age and sex were controlled for using residuals which were then standardised. These were then subjected to a maximum likelihood factor analysis. These was then summed with the age and sex controlled standardised residuals from the Mill Hill vocabulary test (Raven, 1965) with the mean being used as the measure of intelligence (Hill et al., 2014a). In the replication sample consisted of the BATS cohort which consists of 2062 individuals (females = 1093) with a mean age of 16.6 years (SD = 1.5). The test used as a measure of intelligence was the full scale IQ score from the Multidimensional Aptitude Battery (Jackson, 1984) corrected for age and sex with regression. The standardised residuals from this model were used in subsequent genetic analysis.

Genome wide association had been carried out in each cohort of CAGES using Mach2QTL (Li et al., 2010) before being meta-analysed in METAL (Willer et al., 2010) using an inverse variance weighted model. SNPs were then assigned to genes based on their position in the UCSC human genome browser hg 18 assembly with a 50kb boundary around each gene to capture any regulatory elements. A gene based statistic was derived using VEGAS (Liu et al., 2010) to control for the number of SNPs assigned to each gene as well as patterns of linkage disequilibrium. In order to test the principal hypothesis that the genes of the Arc complex (See Table S11) will show a greater association, as a set, than those drawn from across the genome, each gene based p value was –log10 transformed and rank ordered. Gene Set Enrichment Analysis (GSEA) (Subramanian et al., 2005; Wang et al., 2007), a competitive test of enrichment, was then used to determine if the gene identifiers for the Arc gene set fall higher in the genome-wide rankings than would be expected by chance alone (Hill et al., 2014a; Hill et al., 2014b). This was done for each set by deriving a Kolmogorov-Smirnov (K-S) statistic weighted by the p-values of the gene based statistic in order to take into account both the ranks and the distance between ranks. Following this the genome wide ranked set was permuted and the K-S statistic calculated again. Statistical significance was established by using 15,000 permutations of the genome wide ranked set with the p-value describing the proportion of permuted K-S tests smaller than the original un-permuted K-S statistic. Statistical significance was set at < 0.05 and FDR <0.25 (Subramanian et al., 2005; Wang et al., 2007).

21. Statistics

Hippocampal slices

In electrophysiological experiments, since several slices were routinely prepared from every mouse, AUC_{I-O}, PPF and LTP values were compared between wild-type and mutant mice using the two-way nested ANOVA with genotype (group) and mice (sub-group) as fixed and random factors correspondingly (STATISTICA v. 10, StatSoft, Inc., Tulsa, OK, USA). DF error was computed using the Satterthwaite method and main genotype effect was considered significant if $P < 0.05$. Graph plots and normalization were performed using OriginPro 8.5 (OriginLab, Northampton, MA, USA). Electrophysiological data are presented as the mean ± s.e.m. with n and N indicating number of slices and mice respectively.

Protein quantification

Comparisons between two groups were performed using two-tailed Mann-Whitney U test. Significance was accepted to $P <$ 0.05. Data are presented as mean ± s.e.m.

Genetics

P values were corrected by a Bonferroni multiple test. We initially performed 4 tests for the 4 Arc complexes for CNVs, 8 tests for Lof/NS combined analyses, and 40 tests for LoF/NS in individual studies. However, at each successive stage we also accounted for all of the tests previously performed, giving a total of 4 tests for de novo CNVs, 12 tests for combined NS/LoF and 52 tests for NS/LoF studies. Fisher's method was used to combine p-values from the 5 independent genetic datasets ('Combined' p-value for LoF and NS mutations).

22. Known Arc interactors.

Arc interactors were manually curated from literature. Arc interactors previously reported have been manually annotated. Protein IDs (UniProt accession numbers) as well as protein and gene names are shown. PubMed ID and the interaction detection method are also indicated.

Protein ID	Gene name	Protein name	PMID
P28652	*Camk2b*	Calcium/calmodulin-dependent protein kinase type II subunit beta	22579289
P35438;P35438-2	*Grin1*	Glutamate receptor ionotropic, NMDA 1	16635246 10862698
G3X9V4;Q01097	*Grin2b*	Glutamate receptor ionotropic, NMDA 2B	10862698 25864631
P49768	*Psen1*	Presenilin-1	22036569
Q62108;Q62108-3;Q62108-2;G3UZL5	*Dlg4*	Disks large homolog 4	19455133
Q80TN1;P11798; F8WIS9	*Camk2a*	Calcium/calmodulin-dependent protein kinase type II subunit alpha	22579289
P39054	*Dnm2*	Dynamin-2	17088211
Q62419	*Sh3gl1*	Endophilin-A2	17088211
Q62421	*Sh3gl3*	Endophilin-A3	17088211
O88602	*Cacng2*	TARPg2	25864631
Q9D415	*Dlgap1*	Disks large-associated protein 1	25864631
Q8R5H6	*Wasf1*	Wiskott-Aldrich syndrome protein family member 1	25864631
P35436	*Grin2a*	Glutamate receptor ionotropic, NMDA 2A	25864631
Q5DU25	*Iqsec2*	IQ motif and SEC7 domain-containing protein 2	25864631

23. Oligos

Oligo name	Sequence 5'3
ArcHA1XbaR	CTGC TCT AGA TTC AGG CTG GGT CCT GTC ACT G
ArcHA1XhoF	ATC TCG CTC GAG GGG AGG TCT TCT ACC GTC TGG AG
ArcHA2BglR	GACCT AGA TCT GC GGCC GC GGG GCC AGG AGG TGC CAG GAT GTC AGG TC
ArcHA2Acc65F	CTACGG GGT ACC AGG GGC CAG CCC AGG GTC CCC AG
ArcHA3NdeF	GGG TAA TCTC CA TAT G GGA GGC TTA GGC TAG GCA CAG AC
ArcHA3PmlR	CCG GCG TA CAC GTG CCC TGG TCC CAG CCA TGA TTC ATA AG
ArcHA4AscF	CATTT G GCG CGC C CCA ATA GGT CAT CAC AAC TGC CAT G
ArcHA4PmeR	AGC TTT G TTT AAA C GCC CTT GCA CCT CTT GCT GCA C
PneoF1	TAC AAA TAA GC AAT AGC ATC AC
Pneo3F	GCC TCT GAG CTA TTC CAG AAG TAG
Pneo3R	CCT GAC TAG GGG AGG AGT AGA AG
Arc10R	GGG CTT CTT ATG TTC AGT C
Arc1F	CGG GAC CTG TAC CAG ACA CTG
Arc1R	GCC ATG GCT GAG TCA CGG AG
Arc2F	GGA GCG AGA GCT GAA AGG GTT G
Arc2R	GGG GCC AGG AGG TGC CAG GAT GTC
Arc3R	CCC TCA GCA TCT CTG CTT TAG C
Arc4R	GCC AGG ACT AGG TTG GAC AGT CTG
Arc5F	CCC TCC TGG ATC TAG TGG TGA GC
Arc5R	GGGCATCCACTCACATAACTACTCG
Arc6F	GCC CCT GCC CAG CCT GAT CTT TC
Arc6R	CCC CGA GAC ACT ACT CTG AGC
Arc7F	GGG TGG ACA GTT GGT CAG AAG G
Arc7R	CCC TCT GAC CAC TGC ATT TTC C
Arc8F	CCC AGT GCT TTC TCA GCC TTC ATG
Arc8R	GGT CAG TGG GTG AGT AGA TGT CTG
Arc9F	CGG AGG CAC TCA CAC CTG
Arc9R	GGT TGT GTG GCA GTT GTG AGT C

Arc10F	GCT CTT ACC AGC GAG
Arc11F	GGG CAG ATG CTA AAG CAG
Arc11R	CGG CTG GGT GTG AGG ACT CAG
Venus F	CGG CGC GCT AGC GGT GGC GGT AGT ATG GTG AGC AAG GGC GAG GAG C
VenusR	AAC CGT CCT GCA GGT CAC TTG TAC AGC TCG TCC ATG CCG AG
AcExon F2	CAG AGG ATG AGA CGG AGG CAC TC
ArcUTR R2	AGG GGC TTC TTG ATG TTC AGT CC

References

(2014). Activities at the Universal Protein Resource (UniProt). Nucleic acids research *42*, D191-198.

Alexa, A., Rahnenfuhrer, J., and Lengauer, T. (2006). Improved scoring of functional groups from gene expression data by decorrelating GO graph structure. Bioinformatics *22*, 1600-1607.

Alonso-Lopez, D., Gutierrez, M.A., Lopes, K.P., Prieto, C., Santamaria, R., and De Las Rivas, J. (2016). APID interactomes: providing proteome-based interactomes with controlled quality for multiple species and derived networks. Nucleic acids research *44*, W529-535.

Aranda, B., Blankenburg, H., Kerrien, S., Brinkman, F.S., Ceol, A., Chautard, E., Dana, J.M., De Las Rivas, J., Dumousseau, M., Galeota, E., *et al.* (2011). PSICQUIC and PSISCORE: accessing and scoring molecular interactions. Nature methods *8*, 528-529.

Bader, G.D., Betel, D., and Hogue, C.W. (2003). BIND: the Biomolecular Interaction Network Database. Nucleic acids research *31*, 248-250.

Binns, D., Dimmer, E., Huntley, R., Barrell, D., O'Donovan, C., and Apweiler, R. (2009). QuickGO: a web-based tool for Gene Ontology searching. Bioinformatics *25*, 3045-3046.

Calderone, A., Castagnoli, L., and Cesareni, G. (2013). mentha: a resource for browsing integrated protein-interaction networks. Nature methods *10*, 690-691.

Cattell, R.B., and Cattell, A.K.S. (1960). The individual or group Culture Fair Intelligence Test (Champaign, Ill: IPAT).

Chatr-Aryamontri, A., Breitkreutz, B.J., Heinicke, S., Boucher, L., Winter, A., Stark, C., Nixon, J., Ramage, L., Kolas, N., O'Donnell, L., *et al.* (2013). The BioGRID interaction database: 2013 update. Nucleic acids research *41*, D816-823.

Chowdhury, S., Shepherd, J.D., Okuno, H., Lyford, G., Petralia, R.S., Plath, N., Kuhl, D., Huganir, R.L., and Worley, P.F. (2006). Arc/Arg3.1 interacts with the endocytic machinery to regulate AMPA receptor trafficking. Neuron *52*, 445-459.

Coba, M.P., Komiyama, N.H., Nithianantharajah, J., Kopanitsa, M.V., Indersmitten, T., Skene, N.G., Tuck, E.J., Fricker, D.G., Elsegood, K.A., Stanford, L.E., *et al.* (2012). TNiK is required for postsynaptic and nuclear signaling pathways and cognitive function. The Journal of neuroscience : the official journal of the Society for Neuroscience *32*, 13987-13999.

Cox, J., and Mann, M. (2008). MaxQuant enables high peptide identification rates, individualized p.p.b.-range mass accuracies and proteome-wide protein quantification. Nature biotechnology *26*, 1367-1372.

Deary, I.J., Gow, A.J., Taylor, M.D., Corley, J., Brett, C., Wilson, V., Campbell, H., Whalley, L.J., Visscher, P.M., Porteous, D.J., *et al.* (2007). The Lothian Birth Cohort 1936: a study to examine influences on cognitive ageing from age 11 to age 70 and beyond. BMC Geriatr *7*, 28.

Deary, I.J., Whalley, L.J., and Starr, J.M. (2009). A Lifetime of Intelligence, Follow-up Studies of the Scottish Mental Surveys of 1932 and 1947 (Washington, DC: American Psychological Association).

Deary, I.J., Whiteman, M.C., Starr, J.M., Whalley, L.J., and Fox, H.C. (2004). The impact of childhood intelligence on later life: Following up the Scottish Mental Surveys of 1932 and 1947. J Pers Soc Psychol *86*, 130-147.

Fernandez, E., Collins, M.O., Uren, R.T., Kopanitsa, M.V., Komiyama, N.H., Croning, M.D., Zografos, L., Armstrong, J.D., Choudhary, J.S., and Grant, S.G. (2009). Targeted tandem affinity purification of PSD-95 recovers core postsynaptic complexes and schizophrenia susceptibility proteins. Molecular systems biology *5*, 269.

Frank, R.A., Komiyama, N.H., Ryan, T.J., Zhu, F., O'Dell, T.J., and Grant, S.G. (2016). NMDA receptors are selectively partitioned into complexes and supercomplexes during synapse maturation. Nat Commun *7*, 11264.

Fromer, M., Pocklington, A.J., Kavanagh, D.H., Williams, H.J., Dwyer, S., Gormley, P., Georgieva, L., Rees, E., Palta, P., Ruderfer, D.M., *et al.* (2014). De novo mutations in schizophrenia implicate synaptic networks. Nature *506*, 179-184.

Guildford, J.P., Christensen, P.R., Merrifield, P.R., and Wilson, J., C (1978). Alternate uses: manual of instructions and interpretation (Orange, Calif: Sheridan Psychological Service).

Guzowski, J.F., Lyford, G.L., Stevenson, G.D., Houston, F.P., McGaugh, J.L., Worley, P.F., and Barnes, C.A. (2000). Inhibition of activity-dependent arc protein expression in the rat hippocampus impairs the maintenance of long-term potentiation and the consolidation of long-term memory. The Journal of neuroscience : the official journal of the Society for Neuroscience *20*, 3993-4001.

Heim, A. (1970). AH 4 Group Test of General Intelligence Manual, (Windsor, UK: NFER Publishing Company).

Hill, W.D., Davies, G., van de Lagemaat, L.N., Christoforou, A., Marioni, R.E., Fernandes, C.P.D., Liewald, D.C., Croning, M.D.R., Payton, A., Craig, L.C.A., *et al.* (2014a). Human cognitive ability is influenced by genetic variation in components of postsynaptic signalling complexes assembled by NMDA receptors and MAGUK proteins. Transl Psychiatry *4*, e341.

Hill, W.D., de Leeuw, C., Davies, G., Liewald, D.C.M., Payton, A., Craig, L.C., J.W., L., Horan, M., Ollier, W., Starr, J.M., *et al.* (2014b). Functional Gene Group Analysis Indicates No Role for Heterotrimeric G Proteins in Cognitive Ability. PloS one *9*.

Jackson, D.N. (1984). Multidimensional Aptitude Battery manual., Vol 1 (Port Huron, MI: Research Psychologists Press).

Jensen, L.J., Kuhn, M., Stark, M., Chaffron, S., Creevey, C., Muller, J., Doerks, T., Julien, P., Roth, A., Simonovic, M., *et al.* (2009). STRING 8--a global view on proteins and their functional interactions in 630 organisms. Nucleic acids research *37*, D412-416.

Kirov, G., Pocklington, A.J., Holmans, P., Ivanov, D., Ikeda, M., Ruderfer, D., Moran, J., Chambert, K., Toncheva, D., Georgieva, L., *et al.* (2012). De novo CNV analysis implicates specific abnormalities of postsynaptic signalling complexes in the pathogenesis of schizophrenia. Molecular psychiatry *17*, 142-153.

Kopanitsa, M.V., Afinowi, N.O., and Grant, S.G. (2006). Recording long-term potentiation of synaptic transmission by three-dimensional multi-electrode arrays. BMC Neurosci *7*, 61.

Lezak, M., Howieson, D.B., and Loring, D.W. (2004). Neuropsychological Assessment, 4 edn (New York: Oxford University Press, USA).

Li, Y., Willer, C.J., Ding, J., Scheet, P., and Abecasis, G.R. (2010). MaCH: using sequence and genotype data to estimate haplotypes and unobserved genotypes. Genetic epidemiology *34*, 816-834.

Liu, J.Z., McRae, A.F., Nyholt, D.R., Medland, S.E., Wray, N.R., Brown, K.M., Hayward, N.K., Montgomery, G.W., Visscher, P.M., Martin, N.G., *et al.* (2010). A versatile gene-based test for genome-wide association studies. American journal of human genetics *87*, 139-145.

Liu, P., Jenkins, N.A., and Copeland, N.G. (2003). A highly efficient recombineering-based method for generating conditional knockout mutations. Genome Res *13*, 476-484.

Messaoudi, E., Kanhema, T., Soule, J., Tiron, A., Dagyte, G., da Silva, B., and Bramham, C.R. (2007). Sustained Arc/Arg3.1 synthesis controls long-term potentiation consolidation through regulation of local actin polymerization in the dentate gyrus in vivo. The Journal of neuroscience : the official journal of the Society for Neuroscience *27*, 10445-10455.

Nelson, H.E., and Willison, J. (1991). National Adult Reading Test (NART) Test Manual, 2nd edn (Windsor: NFER-Nelson).

Orchard, S., Ammari, M., Aranda, B., Breuza, L., Briganti, L., Broackes-Carter, F., Campbell, N.H., Chavali, G., Chen, C., del-Toro, N., *et al.* (2014). The MIntAct project--IntAct as a common curation platform for 11 molecular interaction databases. Nucleic acids research *42*, D358-363.

Prasad, T.S., Kandasamy, K., and Pandey, A. (2009). Human Protein Reference Database and Human Proteinpedia as discovery tools for systems biology. Methods Mol Biol *577*, 67-79.

Purcell, S.M., Moran, J.L., Fromer, M., Ruderfer, D., Solovieff, N., Roussos, P., O'Dushlaine, C., Chambert, K., Bergen, S.E., Kahler, A., *et al.* (2014). A polygenic burden of rare disruptive mutations in schizophrenia. Nature *506*, 185-190.

Raven, J.C. (1965). The Mill Hill vocabulary scale (London: H.K. Lewis).

Raven, J.C., Court, J.H., and Raven, J. (1977). Manual for Raven's Progressive Matrices and Vocabulary Scales (London: H.K. Lewis).

Salwinski, L., Miller, C.S., Smith, A.J., Pettit, F.K., Bowie, J.U., and Eisenberg, D. (2004). The Database of Interacting Proteins: 2004 update. Nucleic acids research *32*, D449-451.

Scottish Council for Research in Education (1933). The intelligence of Scottish children: A national survey of an age-group (London: University of London Press).

Shepherd, J.D., Rumbaugh, G., Wu, J., Chowdhury, S., Plath, N., Kuhl, D., Huganir, R.L., and Worley, P.F. (2006). Arc/Arg3.1 mediates homeostatic synaptic scaling of AMPA receptors. Neuron *52*, 475-484.

Steward, O., Wallace, C.S., Lyford, G.L., and Worley, P.F. (1998). Synaptic activation causes the mRNA for the IEG Arc to localize selectively near activated postsynaptic sites on dendrites. Neuron *21*, 741-751.

Subramanian, A., Tamayo, P., Mootha, V.K., Mukherjee, S., Ebert, B.L., Gillette, M.A., Paulovich, A., Pomeroy, S.L., Golub, T.R., Lander, E.S., *et al.* (2005). Gene set enrichment analysis: a knowledge-based approach for interpreting genome-wide expression profiles. Proc Natl Acad Sci U S A *102*, 15545-15550.

UniProt, C. (2014). Activities at the Universal Protein Resource (UniProt). Nucleic acids research *42*, D191-198.

Valor, L.M., Charlesworth, P., Humphreys, L., Anderson, C.N., and Grant, S.G. (2007). Network activity-independent coordinated gene expression program for synapse assembly. Proceedings of the National Academy of Sciences of the United States of America *104*, 4658-4663.

Wang, K., Li, M., and Bucan, M. (2007). Pathway-based approaches for analysis of genomewide association studies. American journal of human genetics *81*, 1278-1283.

Wechsler, D. (1981). Manual for the Wechsler adult intelligence scale – Revised. (New York: The Psychological Corporation).

Wechsler, D. (1987). Wechsler Memory Scale—Revised (New York: Psychological Corporation).

Wechsler, D. (1998). Wechsler Adult Intelligence Scale - third edition (London: The Psychological Corporation).

Willer, C.J., Li, Y., and Abecasis, G.R. (2010). METAL: fast and efficient meta-analysis of genomewide association scans. Bioinformatics *26*, 2190-2191.
