# Supplementary material for: Arc Requires PSD95 for Assembly into Postsynaptic Complexes Involved with Neural Dysfunction and Intelligence
Source: Cell Rep. 2017 Oct 17;21(3):679–91. doi: 10.1016/j.celrep.2017.09.045 (PMC5656750; doi:10.1016/j.celrep.2017.09.045)
Supplement: Document S2. Article plus Supplemental Information [file mmc13.pdf]

Cell Reports

Article

Arc Requires PSD95 for Assembly into Postsynaptic Complexes Involved with Neural Dysfunction and Intelligence

Graphical Abstract

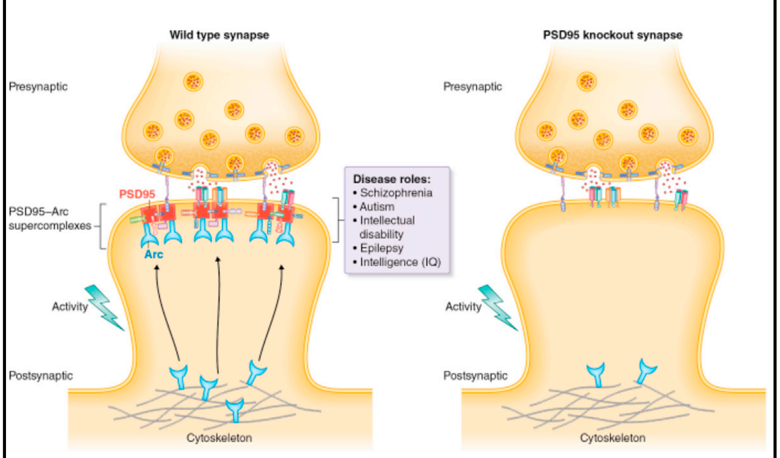

Authors

Esperanza Fernández, Mark O. Collins, René A.W. Frank, ..., Jyoti S. Choudhary, Noboru H. Komiyama, Seth G.N. Grant

Correspondence

seth.grant@ed.ac.uk

In Brief

Fernández et al. use genetics and proteomics to study the Arc protein in the mouse brain. PSD95 recruits Arc to the synapse and assembles it into signaling complexes with neurotransmitter receptors and other proteins. Arc-PSD95 supercomplexes contain genetic variants previously linked to epilepsy, schizophrenia, intellectual disability, and IQ.

Highlights

- TAP tag and purification of endogenous Arc protein complexes from the mouse brain
- PSD95 is the major Arc binding protein, and both assemble into 1.5-MDa supercomplexes
- PSD95 is essential for recruitment of Arc to synapses
- Mutations and genetic variants in Arc-PSD95 are linked to cognition

Data and Software Availability

PXD007283

Fernández et al., 2017, Cell Reports *21*, 679–691
October 17, 2017 © 2017 The Author(s).
https://doi.org/10.1016/j.celrep.2017.09.045

Cell Reports
Article

OPEN ACCESS
CellPress

Arc Requires PSD95 for Assembly into Postsynaptic Complexes Involved with Neural Dysfunction and Intelligence

Esperanza Fernández,[1,12,15] Mark O. Collins,[2,14] René A.W. Frank,[1,3] Fei Zhu,[1,4] Maksym V. Kopanitsa,[1,5,16] Jess Nithianantharajah,[1,4] Sarah A. Lemprière,[4] David Fricker,[1,5] Kathryn A. Elsegood,[1,4] Catherine L. McLaughlin,[4] Mike D.R. Croning,[4] Colin Mclean,[6] J. Douglas Armstrong,[6] W. David Hill,[7] Ian J. Deary,[7] Giulia Cencelli,[12,13] Claudia Bagni,[12,13,17] Menachem Fromer,[8,9,10] Shaun M. Purcell,[10] Andrew J. Pocklington,[11] Jyoti S. Choudhary,[2] Noboru H. Komiyama,[1,4] and Seth G.N. Grant[1,4,18,*]

[1]Genes to Cognition Programme, The Wellcome Trust Sanger Institute, Hinxton, Cambridgeshire, UK
[2]Proteomic Mass Spectrometry, The Wellcome Trust Sanger Institute, Hinxton, Cambridgeshire, UK
[3]Medical Research Council Laboratory of Molecular Biology, Cambridge, UK
[4]Genes to Cognition Programme, Centre for Clinical Brain Science, University of Edinburgh, Edinburgh, UK
[5]Synome Ltd., Moneta Building, Babraham Research Campus, Cambridge, UK
[6]School of Informatics, Institute for Adaptive and Neural Computation, University of Edinburgh, UK
[7]Centre for Cognitive Ageing and Cognitive Epidemiology, Department of Psychology, University of Edinburgh, UK
[8]Analytic and Translational Genetics Unit, Massachusetts General Hospital, Boston, MA 02114, USA
[9]Stanley Center for Psychiatric Research, Broad Institute of MIT and Harvard, Cambridge, MA 02142, USA
[10]Division of Psychiatric Genomics, Department of Psychiatry, Icahn School of Medicine at Mount Sinai, New York, NY 10029, USA
[11]Institute of Psychological Medicine & Clinical Neurosciences, University of Cardiff, Cardiff, Wales, UK
[12]KU Leuven, Center for Human Genetics and Leuven Institute for Neurodegenerative Diseases (LIND), and VIB Center for the Biology of Disease, Leuven, Belgium
[13]Department of Biomedicine and Prevention, University of Rome Tor Vergata, Rome, Italy
[14]Present address: Department of Biomedical Science, The Centre for Membrane Interactions and Dynamics, University of Sheffield, Sheffield, UK
[15]Present address: VIB-UGent Center for Medical Biotechnology, Gent, Belgium
[16]Present address: Charles River Discovery, 70210 Kuopio, Finland
[17]Present address: Department of Neuroscience, University of Lausanne, Lausanne, Switzerland
[18]Lead Contact
*Correspondence: seth.grant@ed.ac.uk
https://doi.org/10.1016/j.celrep.2017.09.045

SUMMARY

Arc is an activity-regulated neuronal protein, but little is known about its interactions, assembly into multiprotein complexes, and role in human disease and cognition. We applied an integrated proteomic and genetic strategy by targeting a tandem affinity purification (TAP) tag and Venus fluorescent protein into the endogenous *Arc* gene in mice. This allowed biochemical and proteomic characterization of native complexes in wild-type and knockout mice. We identified many Arc-interacting proteins, of which PSD95 was the most abundant. PSD95 was essential for Arc assembly into 1.5-MDa complexes and activity-dependent recruitment to excitatory synapses. Integrating human genetic data with proteomic data showed that Arc-PSD95 complexes are enriched in schizophrenia, intellectual disability, autism, and epilepsy mutations and normal variants in intelligence. We propose that Arc-PSD95 postsynaptic complexes potentially affect human cognitive function.

INTRODUCTION

Arc/Arg3.1 was originally identified as a cytoskeletal-associated protein encoded by an mRNA that was rapidly transcribed following synaptic activity and transported to dendrites (Link et al., 1995; Lyford et al., 1995; Moga et al., 2004; Steward et al., 1998). Many forms of neuronal activation induce Arc: synaptic stimulation, including long-term potentiation (Guzowski et al., 2000); metabotropic glutamate receptor-dependent long-term depression (Jakkamsetti et al., 2013; Park et al., 2008; Waung et al., 2008); homeostatic scaling of AMPA receptors (Gao et al., 2010; Korb et al., 2013; Okuno et al., 2012; Shepherd et al., 2006); generalized neuronal activity induced by seizures (Link et al., 1995); as well as various behavioral stimuli (memory- and experience-related behavioral patterns; Daberkow et al., 2007; Gao et al., 2010; Guzowski et al., 1999; Jakkamsetti et al., 2013; Kelly and Deadwyler, 2003; Miyashita et al., 2009; Vazdarjanova and Guzowski, 2004; Vazdarjanova et al., 2006; Wibrand et al., 2012) and visual stimuli (Wang et al., 2006). Knockout or knockdown of Arc results in impaired synaptic plasticity and hippocampus-dependent learning and behavior phenotypes reminiscent of schizophrenia (Guzowski et al., 2000; Managò et al., 2016; McCurry et al., 2010; Plath et al., 2006; Wang et al., 2006).

This is an open access article under the CC BY license (http://creativecommons.org/licenses/by/4.0/).

Arc is mainly localized at postsynaptic sites of excitatory synapses (Moga et al., 2004). The proteome of the postsynaptic terminal of excitatory synapses of vertebrate species contains a highly conserved set of ~1,000 protein types (Bayés et al., 2011, 2012, 2017; Distler et al., 2014) organized into more than 200 multiprotein complexes (Frank and Grant, 2017; Frank et al., 2016, 2017). The multiprotein complexes are organized into a hierarchy of complexes and supercomplexes (complexes of complexes), and the prototype supercomplex is formed by PSD95 (Fernández et al., 2009; Frank et al., 2016, 2017; Husi and Grant, 2001; Husi et al., 2000). Arc was found to be associated with PSD95 (Fernández et al., 2009; Frank et al., 2016, 2017; Husi et al., 2000), and genetic studies show that absence of either PSD95 or Arc leads to enhanced long-term potentiation (LTP) and impaired hippocampus-dependent learning (Migaud et al., 1998; Plath et al., 2006). Biochemical purification and mouse genetic experiments show that dimers of PSD95 assemble with multiple complexes, including NMDA receptors, potassium channels, and signaling and adhesion proteins. These are not all found within a single supercomplex but are within an extended family of PSD95 supercomplexes ranging in size from 1–3 MDa (Frank and Grant, 2017; Frank et al., 2016, 2017). A large-scale mouse genetic screen of more than 50 postsynaptic proteins found that PSD95 and its close interacting proteins had the strongest phenotypes in synaptic electrophysiology and behavior, indicating that PSD95 supercomplexes are crucial components of the postsynaptic terminal of excitatory synapses (N.H.K., L.N. van de Lagemaat, L.E. Stanford, C.M. Pettit, D.J. Strathdee, K.E. Strathdee, D.G.F., E.J. Tuck, K.A.E., T.J. Ryan, J.N., N.G. Skene, M.D.R.C., and S.G.N.G., unpublished data; M.V.K., L.N. van de Lagemaat, N. Afinowi, D.J. Strathdee, K.E. Strathdee, D.G.F., E.J. Tuck, K.A.E., N.G. Skene, M.D.R.C., N.H.K., and S.G.N.G., unpublished data). Arc has also been proposed to interact with the endocytic machinery (Dynamin and Endophilin-2 and -3) (Chowdhury et al., 2006; Rial Verde et al., 2006; Shepherd et al., 2006). However, Arc multiprotein complexes have not been purified and systematically studied using proteomic mass spectrometry, and thus the identity of its interacting partners and the composition of Arc complexes remain poorly understood.

Characterizing protein complexes in synapses is technically challenging. Gene-tagging of endogenous proteins in the mouse has greatly facilitated purification of intact native complexes and visualization of their subcellular localization and has many advantages over in vitro and recombinant methods (Broadhead et al., 2016; Fernández et al., 2009; Frank and Grant, 2017; Frank et al., 2016). The effect of mutations on complexes and neuronal activation can be combined in mice carrying knockin gene tags, and proteins that are predicted to be largely unstructured and form multivalent interactions, such as Arc (Xue et al., 2010), can be studied in their native context. These advantages have been illustrated by the purification of native NMDA receptor and PSD95 complexes, in which a tandem affinity purification (TAP) tag was inserted into the N terminus of the GluN1 subunit and C terminus of PSD95 by genome engineering (Fernández et al., 2009; Frank et al., 2016). Purification revealed that NMDA receptors and PSD95 were in ~1.5-MDa supercomplexes with channel subunits, PSD95, and PSD93 as major components. Genetic dissection in vivo using mutant mice showed an essential tripartite requirement for PSD95, PSD93, and the GluN2B cytoplasmic domain (Frank et al., 2016). This tripartite interaction was not previously detected using in vitro methods, which typically rely on binary protein interactions. Moreover, like Arc, the GluN2B cytoplasmic domain is predicted to be a structurally unfolded/disordered domain (Ryan et al., 2008), and these domains lack stable tertiary structure and undergo disorder-to-order transitions upon binding or changes in phosphorylation (Bah et al., 2015; Gibbs et al., 2017). We therefore considered that Arc was well suited to the strategy of gene tagging and genetic dissection.

Genetics has been a powerful approach for studying the function of multiprotein complexes in many prokaryotic, eukaryotic, and metazoan organisms, including humans, where disease-causing mutations have been mapped to protein complexes (Babu et al., 2014; Lu et al., 2013; Vidal et al., 2011). Moreover, in recent years, a large number of mutations that disrupt postsynaptic proteins in humans have been identified and found to cause many psychiatric, neurological, and developmental disorders (Bayés et al., 2011, 2014; Brose et al., 2010; Fromer et al., 2014; Grant, 2012, 2013; Grant et al., 2005; Kirov et al., 2012; Pocklington et al., 2006; Purcell et al., 2014). Although mutations in the human *ARC* gene have not been directly linked to any mental disorder, using preliminary proteomic data on Arc-interacting proteins, the proteins in Arc complexes were found to be enriched in disruptive mutations (Purcell et al., 2014), de novo copy-number variants (CNVs) (Kirov et al., 2012), non-synonymous de novo single-nucleotide variants (SNVs), and small insertions or deletions (indels) (Fromer et al., 2014) in schizophrenia cases. ARC protein has been described to accumulate at synapses in Angelman syndrome (Greer et al., 2010) and increased and/or decreased in several animal models of Alzheimer's disease and patient-derived cells (for a review, see Kerrigan and Randall, 2013). These data suggest that Arc is a component of protein complexes that are involved with human cognitive disorders.

In this paper, we have conducted an extensive proteomic and genetic dissection of Arc protein complexes, which is a generic strategy suitable for the characterization of potentially any synaptic protein. We have focused on the following four challenges: isolation of native multiprotein complexes from brain tissue; visualization of the endogenous protein using genetic tagging; genetic dissection of protein complex organization and localization using mouse genetic models; and genetic dissection of complexes using human genetic data, including human disease and cognitive phenotypes. Here we demonstrate that this integrated proteomic and genetic strategy reveals insights into the physiological functions of Arc and the synaptic basis of mental disorders and intelligence.

RESULTS

TAP Tagging and Proteomic Analysis of Endogenous Arc Complexes

To label and isolate endogenous Arc protein, we engineered knockin mice (Arc^{TAP}) harboring a TAP tag fused to the C terminus of Arc (Figures 1A–1D). Mice carrying the TAP tag showed no

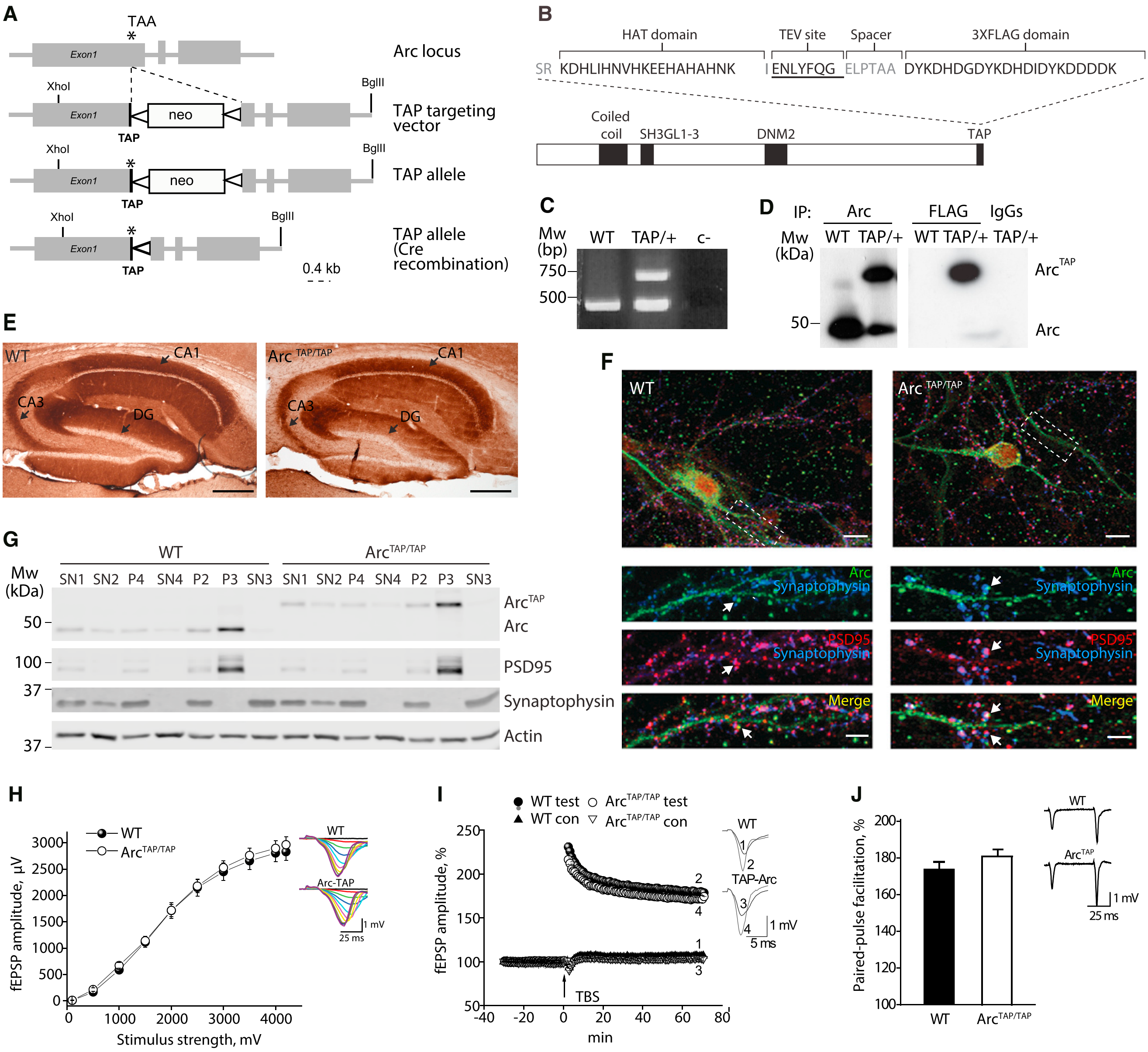

Figure 1. Generation of TAP-Tagged Arc Knockin Mice

(A) Scheme of the genomic Arc locus targeted with the TAP tag. The TAP sequence was inserted before the stop codon of the protein. The cross of Arc^{TAP} knockin mice with a transgenic Cre-expressing mouse line deleted the neomycin (neo) resistance cassette by recombination between loxP sites. Asterisk, stop codon (TAA) of the coding sequence; thick black line, TAP tag sequence; triangle, loxP site.

(B) Structure of the TAP-tagged Arc regions, including a potential coiled-coil domain, an SH3-endophilin-2 and -3 binding region, a dynamin-2 binding region, and the C-terminal TAP tag sequence domain before the stop codon of the protein.

(C) PCR amplification of WT (bottom band) and TAP Arc-targeted alleles (top band).

(D) TAP-tagged Arc was specifically purified from $Arc^{TAP/+}$ forebrain extracts with anti-Arc and anti-FLAG antibodies and blotted with an anti-Arc antibody.

(E) Hippocampal sections of WT and $Arc^{TAP/TAP}$ mice stained with an anti-Arc antibody. DG, dentate gyrus. Scale bar, 1 mm. Shown is a representative image of n = 2 mice for each genotype.

(F) Representative image of embryonic primary neurons derived from WT and $Arc^{TAP/TAP}$ mouse independent cultures immunostained at day in vitro (DIV) 15 with antibodies against Arc (green), PSD-95 (red), and Synaptophysin (blue). Merged, colocalization of the three signals. Arrows show the punctum labeling for each protein. Scale bar, 10 μm.

(G) Biochemical fractionation from $Arc^{TAP/TAP}$ and WT mouse forebrains. Similar protein amounts from each fraction were loaded onto a gel and immunoblotted with the antibodies displayed at the right. TAP-tagged Arc showed the same subcellular distribution as the WT non-tagged isoform. The fractions are described in the Supplemental Experimental Procedures. Antibodies against synaptophysin were used as a specific marker of the SN3 fraction. Actin was used as a loading control. MW, molecular weight in kilodaltons; TAP/+, heterozygous for TAP-tagged Arc; c-, PCR water; IgG, mouse total immunoglobulin G.

(legend continued on next page)

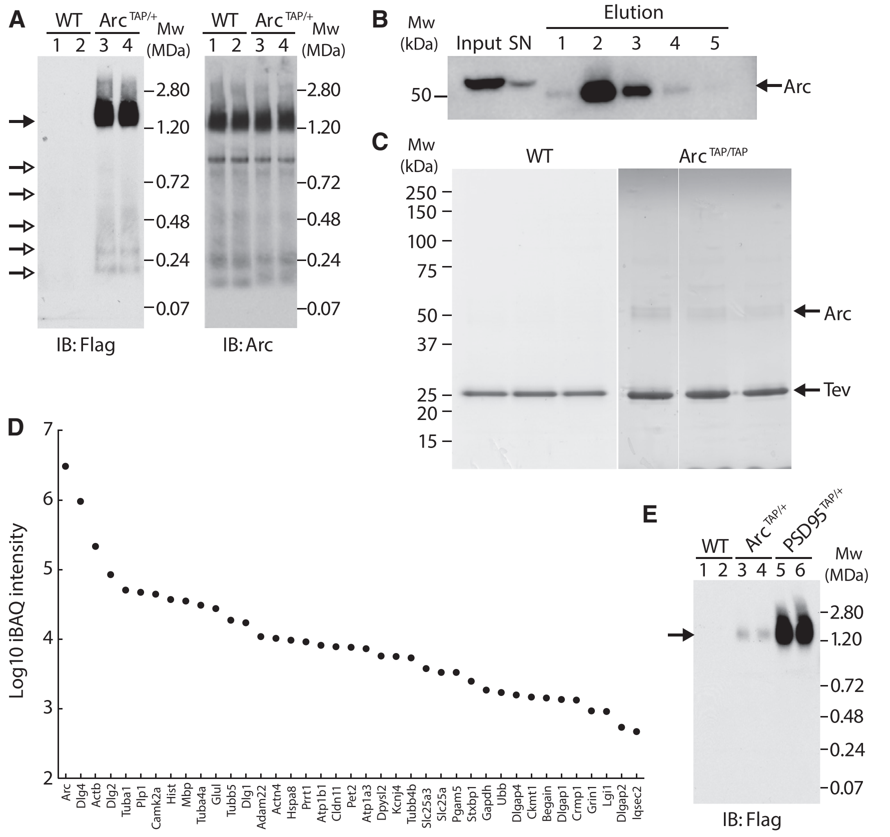

Figure 2. TAP of Arc Reveals Postsynaptic Complexes with a Native Size of 1.5 MDa

(A) BNP of WT and $Arc^{TAP/+}$ forebrain extracts blotted with FLAG and Arc antibodies, where Arc and TAP-tagged Arc can be mainly detected at 1.5 MDa. The closed arrow indicates the main Arc complex, whereas open arrows indicate lower-molecular-weight Arc complexes.

(B) Arc was tandem affinity-purified from $Arc^{TAP/TAP}$ forebrain extracts, eluted, and collected in 5 consecutive fractions following histidines affinity tag (HAT) purification.

(C) Colloidal Coomassie staining of three independent TAPs from WT (left) and $Arc^{TAP/TAP}$ (right) forebrains. The lanes were cut for LC-MS/MS, and the identified proteins are listed in Table S1. Arc and the Tev enzyme are indicated.

(D) The absolute expression value of each protein in the tandem purification was estimated by the iBAQ intensity values obtained in each purification.

(E) BNP of WT, $Arc^{TAP/+}$, and $PSD95^{TAP/+}$ forebrain extracts blotted with FLAG antibody. TAP-tagged Arc and TAP-tagged PSD95 levels are detected. TAP/+, heterozygous for TAP-tagged Arc or PSD95 as indicated; SN, supernatant.

detectable alterations in the levels or localization of Arc in the brain or in hippocampal synaptic physiology (Figures 1E–1J). Native Arc complexes were detected by immunoblotting of brain extracts separated on blue native PAGE (BNP), which showed a major band of a median mass of ~1.5 MDa, with several additional minor species ranging from ~200–700 kDa (Figure 2A).

The TAP tag was used to isolate Arc complexes directly from mouse forebrain tissue using a highly efficient purification protocol (recovering >70% Arc) (Figures 2B and 2C), and their composition was determined using liquid chromatography-tandem mass spectrometry (LC-MS/MS). The single-step purification yielded 107 high-confidence proteins, whereas the more stringent tandem purification protocol recovered a subset of 39 proteins (34 of 39 were uncovered by single-step purification) (Experimental Procedures; Table S1; http://www.genes2cognition.org/publications/tap-arc). Eight of 14 previously reported Arc interactors were found among the 107 high-confidence proteins, indicating that 99 were novel interactors (Supplemental Experimental Procedures). Among the 107 high-confidence proteins, 72 proteins contain the Arc-N lobe consensus motif P[STVILMKR][FYH] (Zhang et al., 2015), revealing a strong network of direct interactors (Table S1). Comparisons of mouse and human show that 87% (92 of 107) of Arc-interacting proteins were conserved between species (Table S2), 70% (1,012 in human and 1,447 in mice) of protein-protein interactions were conserved (Table S3), and the Arc interactome was enriched (72%) in proteins in the human postsynaptic complexes found by Bayés et al. (2012) (Table S3). Together, these results suggest that we have defined a robust Arc complex and interactome that is highly conserved between mouse and human.

PSD95 was the most abundant Arc-interacting protein. Using intensity-based absolute quantification (iBAQ) quantification (Schwanhäusser et al., 2011) of the single-step purification, it showed ~1:1 stoichiometry with Arc (Figure 2D; Table S4), and in the tandem-purification, it represented 57% of the Arc interactome (Table S4). Reciprocal immunoprecipitations show that PSD95 assembles into Arc complexes from early developmental stages (post-natal day 11 [P11]) in the hippocampus and cortex (Figures S1A and S1B). The Dlg family of adaptor/scaffold proteins, comprising four paralogs (SAP97/*Dlg1*, PSD93/*Dlg2*, SAP102/*Dlg3*, and PSD95/*Dlg4*), was the most abundant of

(H) Basal synaptic transmission was normal in $Arc^{TAP/TAP}$ mice. Areas under input-output curves were not statistically different in $Arc^{TAP/TAP}$ (n = 15, N = 5) and WT animals (n = 18, N = 5) ($F_{(1,7.06)} = 0.258$; p = 0.627).

(I) Normalized magnitude of the LTP 60–65 min after LTP induction did not differ in mutant mice (166% ± 5%; n = 15, N = 5; $F_{(1,7.75)} = 0.449$; p = 0.522) relative to their WT counterparts (171% ± 4%; n = 18, N = 5).

(J) Paired-pulse facilitation was not statistically different ($F_{(1,7.36)} = 2.405$; p = 0.163) in $Arc^{TAP/TAP}$ animals (n = 15, N = 5) compared with their WT littermates (n = 18, N = 5).

Data are presented as mean ± SEM, with n = slices and N = mice.

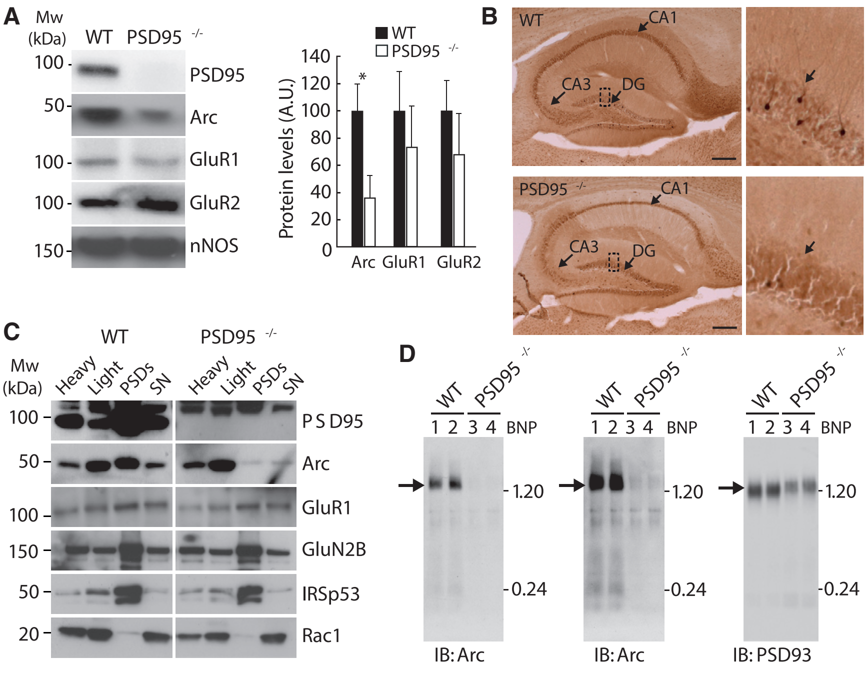

Figure 3. Arc Protein Levels Are Reduced in Fractions of PSD95 Knockout Mice

(A) Representative immunoblot showing the relative abundance of PSD95, Arc, GluR1, and GluR2 proteins in total hippocampal lysates from PSD95$^{-/-}$ and matched WT littermates. Arc is reduced to 35.0% ± 17.1% of the WT in the PSD95 mutant mice (N = 4 for each matched pair, *p < 0.05). Neuronal NOS (nNOS) was used as a loading control.

(B) Representative Arc staining of sagittal sections of the hippocampus (left) and magnification of the granular layer (right) for WT and PSD95 knockout mice. Scale bar, 1 mm.

(C) Hippocampal extracts of PSD95 mutant and WT mutant mice were biochemically fractionated into synaptosomes and into cytoskeletal and vesicular components, referred to as "light." The synaptosomal fraction was subsequently dissociated into PSDs and Triton X-100 soluble fraction. Arc levels were dramatically reduced in the PSD95 mutant, whereas no changes in GluR1, GluN2B, IRSp53, and Rac1 proteins were observed.

(D) Blue native PAGE (BNP) of WT and PSD95 knockout forebrain extracts blotted with Arc and PSD95 antibodies. Long exposure of the blots shows Arc complexes migrating at a lower molecular weight than 1.20 MDa (center). SN, Triton X-100 soluble fraction.

eleven protein classes recovered, suggesting that they play a principal role in regulating Arc function (Figure S1C; Tables S5 and S6). Specificity of interaction between Arc and Dlg paralogs was suggested by the finding that PSD93 and SAP97 were also highly abundant, whereas SAP102 was not detected in the Arc interactome (confirmed using reciprocal immunoprecipitation; Figure S1D). Forty-nine percent of Arc-interacting proteins were known PSD95 interactors and particularly enriched in membrane proteins, including NMDA and AMPA receptors (Table S1).

Consistent with their co-assembly with Arc, the NMDA receptor, PSD95, and PSD93 were also shown to reside in 1.5-MDa supercomplexes (Frank et al., 2016). To compare the relative abundance of Arc and PSD95 in 1.5-MDa supercomplexes, we immunoblotted Arc$^{TAP/+}$ and PSD95$^{TAP/+}$ brain extracts separated by BNP with FLAG antibodies (Figure 2E). PSD95 was ~20-fold more abundant than Arc, indicating that ~5% of PSD95 complexes contain Arc. Pull-down of PSD95 complexes using the TAP tag recovered 65% of PSD95 and depleted 37% of Arc, indicating that ~58% of Arc is in PSD95 supercomplexes (data not shown). Together, these data indicate that PSD95 is the major interacting protein of Arc and that a subset of the postsynaptic 1.5-MDa PSD95 supercomplexes contain Arc.

Arc Postsynaptic Localization Requires PSD95

How Arc is localized to the postsynaptic terminal is unknown. To address this question, we asked whether members of the Dlg scaffold protein family were required in vivo, using mice carrying knockout mutations in PSD95, PSD93, and SAP102 (SAP97 knockout mice are nonviable). In hippocampal extracts, we found that Arc protein levels were reduced (35.0% ± 17.1% of the wild-type [WT], $p < 0.01$) in PSD95 knockout mice but not in PSD93 or SAP102 knockout mice (Figure 3A; Figures S1E and S1F). A dramatic loss of dendritic staining of Arc was observed in hippocampal sections from PSD95 knockout mice (Figure 3B). Furthermore, synaptosomes from PSD95 knockout mice also showed a major reduction in Arc (Figure 3C). We also examined BNP immunoblots from PSD95 knockouts and found that 1.5-MDa Arc complexes were severely diminished, with a weak residual signal after long exposure of the gel (Figure 3D). Thus, PSD95 is specifically required to localize Arc to the postsynaptic terminal.

To visualize endogenous Arc protein, we created Arc^{Venus} knockin mice using a similar design strategy as for the Arc^{TAP} mice, where the Venus fluorescent protein was fused to the C terminus of Arc (Figures 4A–4C). Mice carrying the Venus tag showed no detectable alterations in hippocampal synaptic physiology (Figures 4D–4F). We bred Arc-Venus mice with PSD95 knockouts to generate compound transgenic mice (Arc^{Venus}xPSD95$^{-/-}$) and asked whether kainic acid-induced neuronal activity (Li et al., 2005) would drive Arc to the synapse and whether this required PSD95. In the absence of PSD95, Arc-Venus failed to localize to the synapse (Figure 4G). These results demonstrate that PSD95 is required for the postsynaptic localization of Arc into 1.5-MDa complexes in the steady state and following induction by neuronal activity.

Proteomic Analysis of Arc Complexes in Mice Lacking PSD95

We reasoned that, by genetic removal of PSD95, we could identify those Arc-interacting proteins that were most dependent

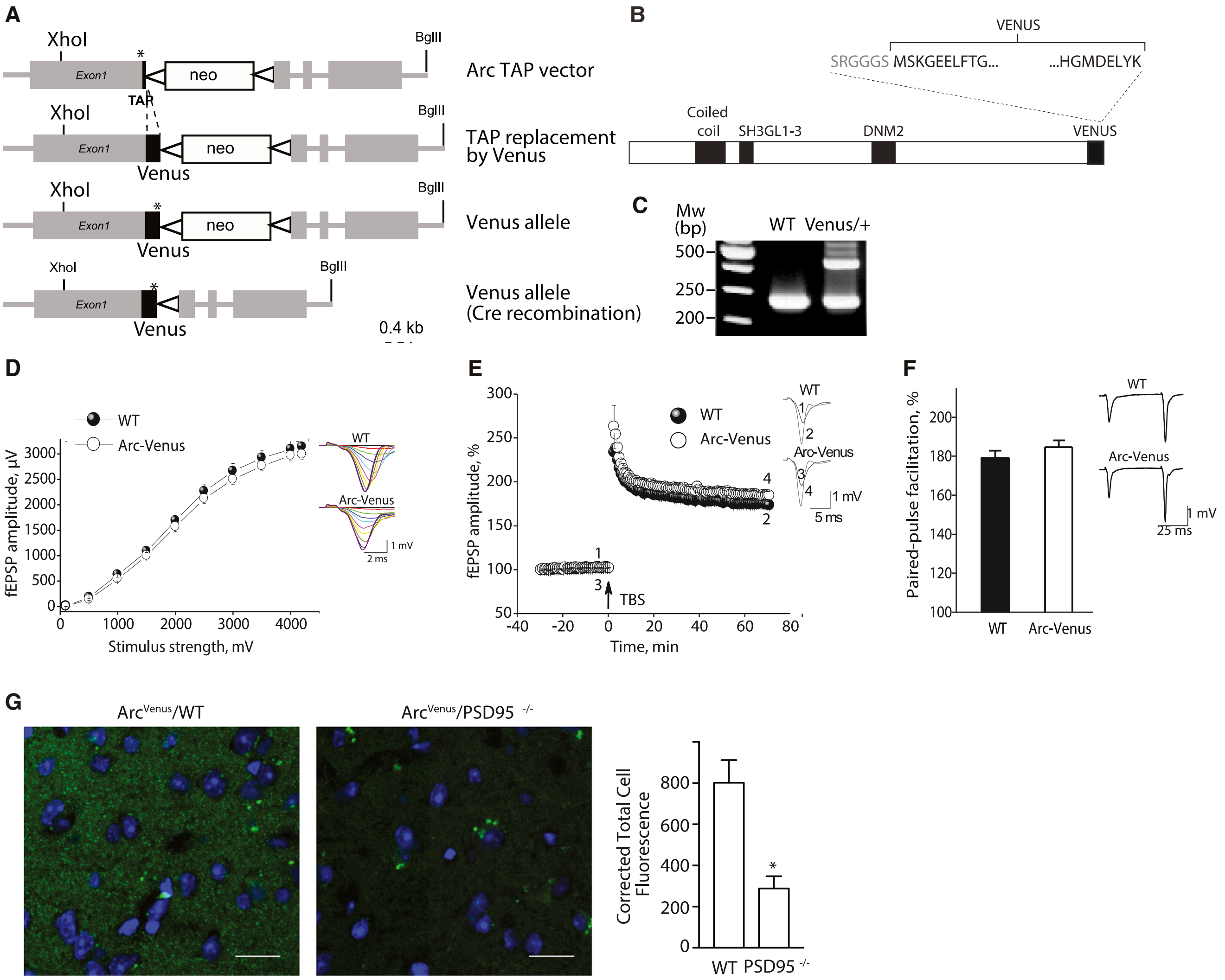

Figure 4. Generation of Venus-Tagged Arc Knockin Mice

(A) The Venus sequence was inserted before the stop codon of the protein using the TAP vector as template. The cross of both knockin mice with a transgenic Cre-expressing mouse line deleted the neo resistance cassette by recombination between loxP-sites. Asterisk, stop codon (TAA) of the coding sequence; thick black line, TAP and Venus tag sequence, as indicated; triangle, loxP site.

(B) Structure of the Venus-tagged Arc regions as in Figure 1B.

(C) PCR amplification of WT (bottom band) and Arc Venus-targeted alleles (top band). Venus/+, heterozygous for Venus-tagged Arc.

(D) Input-output relationships illustrate averaged field excitatory postsynaptic potential (fEPSP) amplitudes in slices from $Arc^{Venus/Venus}$ (n = 26, N = 8) and WT mice (n = 22, N = 8) in response to stimulation of Schaffer collaterals. Areas under input-output curves were not significantly different between genotypes ($F_{(1,13.06)} = 0.499$; p = 0.493).

(E) Normalized magnitude of LTP 60–65 min after LTP induction did not differ significantly in mutant mice (185% ± 4%; n = 25, N = 8; $F_{(1,11.64)} = 2.92$; p = 0.114) relatively to their WT counterparts (174% ± 4%; n = 22, N = 8).

(F) Paired-pulse facilitation was not statistically different ($F_{(1,11.08)} = 1.372$, p = 0.266) in $Arc^{Venus/Venus}$ animals (n = 26, N = 8) compared with their WT littermates (n = 22, N = 8). Data are presented as mean ± SEM, with n = slices and N = mice.

(G) Representative section of Arc^{Venus} mouse brain crossed with WT (left) and $PSD95^{-/-}$ (right) mice. Shown is a bar chart of the total cell fluorescence corrected by the area and the background signal. Scale bars, 15 µm.

on PSD95. We bred $Arc^{TAP/TAP}$ with PSD95 knockout mice ($Arc^{TAP/TAP}/PSD95^{-/-}$) and analyzed their Arc interactome using quantitative proteomic methods (Figures 5A–5C; Table S7). As shown in Figure 5C, Arc-interacting proteins separated into two broad subgroups: depleted and enriched proteins (see red and green proteins, respectively). Seventy percent of depleted proteins were PSD95-interacting proteins, including PSD93. As shown by immunoblots of BNPs, PSD93 remained in 1.5-MDa complexes in $PSD95^{-/-}$ mice (Figure 3D; Frank et al., 2016). Absence of PSD93 did not affect the interaction of Arc with PSD95 (Figure S1G). The most significant gene ontology (GO) biological process (BP) terms in the depleted proteome

OPEN
ACCESS
CellPress

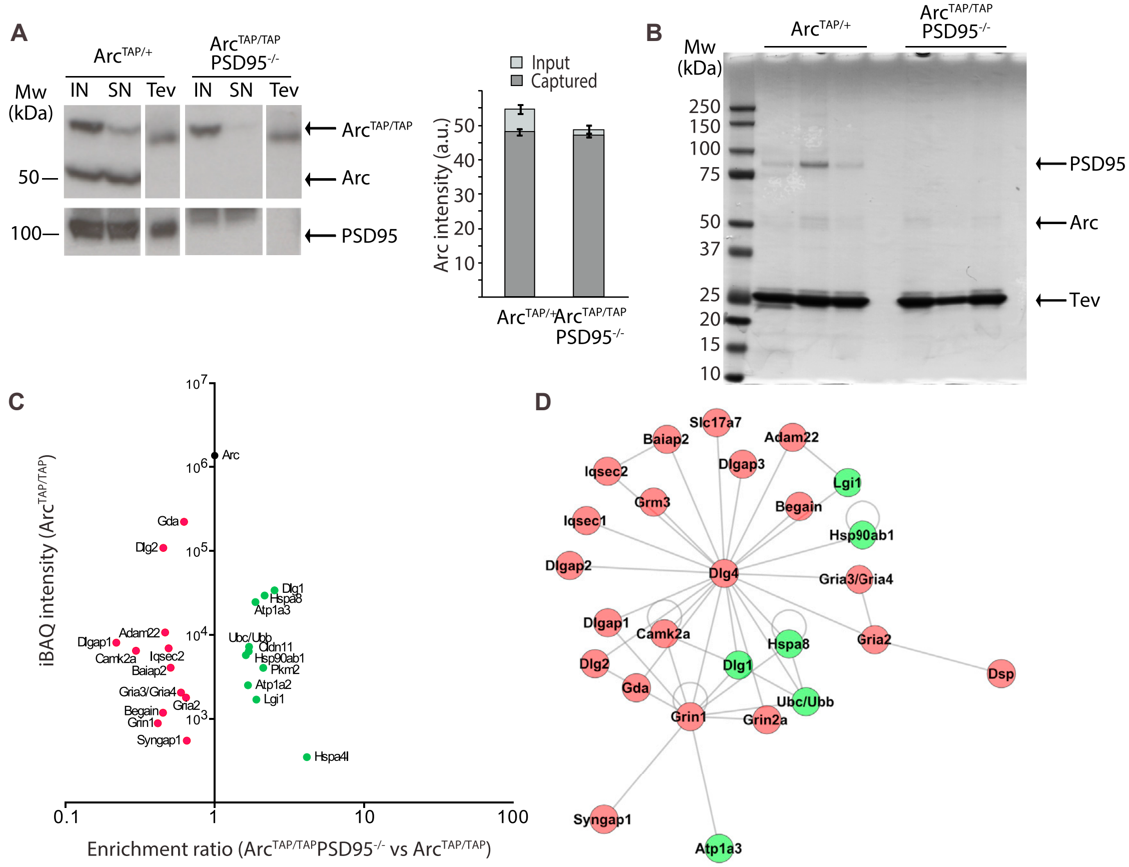

Figure 5. Quantitative Proteomics Analysis of Arc^{TAP} Reveals a Depletion of Postsynaptic Proteins in PSD95 Knockout Mice

(A) Arc complexes were isolated from Arc$^{TAP/+}$ and Arc$^{TAP/TAP}$ mice crossed with PSD95 knockout mice (Arc$^{TAP/TAP}$xPSD95$^{-/-}$) by FLAG capture and Tev protease release (single-step purification). Total lysate (IN, input) and the same volume of lysate upon purification (SN) and Tev elution from both genotypes were blotted against Arc and quantified. Eluted Arc levels following the FLAG capture from Arc$^{TAP/+}$ and Arc$^{TAP/TAP}$xPSD95$^{-/-}$ lysates were not statistically different (Mann-Whitney *U* test, p = 0.1). Data are presented as mean ± SEM.

(B) Isolated complexes from (A) were resolved by SDS-PAGE and stained with colloidal Coomassie. Three independent purifications are shown. The lanes were cut for LC-MS/MS analysis, and the identified proteins are listed in Table S7. TAP-tagged Arc, PSD95, and the Tev enzyme are indicated.

(C) Dimethyl labeling-based quantitative MS of TAP-purified proteins from Arc$^{TAP/+}$ and Arc$^{TAP/TAP}$ crossed with PSD95 knockout mouse forebrain (Arc$^{TAP/TAP}$×PSD95$^{-/-}$). The plot displays enrichment ratios of Arc$^{TAP/TAP}$×PSD95$^{-/-}$ versus Arc$^{TAP/+}$ (x axis) and iBAQ enrichment values of the step purification (y axis). Proteins meeting criteria for enrichment (>1.5 fold) are highlighted in green and for depletion (< 0.667 fold) are highlighted in red. The names of depleted and enriched PSD95 interactors are indicated. See the Supplemental Experimental Procedures for enrichment criteria.

(D) Mouse interactome network constructed from the publicly available databases BioGrid, Database of Interacting Proteins (DIP), IntAct, Molecular INTeraction Database (MINT), STRING database, UniProt, Biomolecular Interaction Network Database (BIND) and mentha using the Psicquic software package. The network is visualized using Visone. Proteins highlighted in green/red meet the enrichment/depletion criteria discussed in the Supplemental Experimental Procedures.

were synaptic transmission (p = 1.23 × 10^{-11}), cell-cell signaling (p = 3.94 × 10^{-9}), and modulation of synaptic transmission (p = 1.35 × 10^{-7}), highlighting the functional importance of the depleted proteins (Table S6).

Among the 12 most enriched proteins in the Arc complexes isolated from PSD95 mutant mice were SAP97 and structural proteins, including those with a potential role in cell growth and adhesion (Claudin11 and Lgi1). A network graph of the interactions of the enriched and depleted protein sets is shown in Figure 5D. The internal network consists of 26 proteins and 44 interactions (visualized using Visone; Brandes and Wagner, 2012). Taken together, these proteomic and in vivo genetic studies show that Arc is tethered to postsynaptic 1.5-MDa signaling complexes containing PSD95, and when these complexes are abolished in PSD95 mutants, Arc is found associated with cytoskeletal and structurally related proteins. Thus, Arc is partitioned into either the PSD95 supercomplexes in the postsynaptic terminal or into cytoskeletal complexes.

Arc Complexes in Disease

Proteins within the postsynaptic proteome are assembled into complexes and supercomplexes (Frank et al., 2016), and this supramolecular organization is of crucial importance in human genetic disorders because it is a mechanism by which the many different gene products functionally converge. We therefore combined our proteomic datasets with human genetic

datasets to understand the importance of Arc-interacting proteins in human disease.

Preliminary proteomic data from Arc^{TAP} mice was previously used to implicate the disruption of ARC complexes in human psychiatric disorders (Fromer et al., 2014; Kirov et al., 2012; Purcell et al., 2014). The first such study revealed that components of Arc complexes were enriched in de novo CNVs from individuals with schizophrenia (Kirov et al., 2012), with subsequent studies finding enrichment for rare point mutations in individuals with schizophrenia, autism, and intellectual disability (ID). Here we extend preliminary proteomic data from Arc^{TAP} mice using complete sets of Arc-interacting proteins and additional genetic datasets, including epilepsy and healthy control de novo datasets.

We first sought to replicate the initial finding of Kirov et al. (2012) with the comprehensive Arc interactome. Utilizing the same genetic dataset as Kirov et al. (2012), we found that de novo CNVs from schizophrenia probands were enriched for Arc complex genes compared with de novo CNVs from unaffected individuals ($p = 0.0047$). Arc interactors whose association with Arc is depleted in PSD95 knockout mice largely drove this enrichment ($p = 0.0165$), indicating the importance of the postsynaptic 1.5-MDa complexes. We next investigated enrichment of the Arc interactome for rare point mutations and indels contributing to brain disorders, using exome sequencing data from a case/control schizophrenia study (Purcell et al., 2014) and de novo studies performed in cohorts of schizophrenia, autism, ID, and epilepsy (Supplemental Experimental Procedures). Combining evidence from each of these independent datasets, we found strong support for the enrichment of both nonsynonymous (NS) and loss-of-function (LoF) disease-related mutations among Arc interactors ($p = 9.01 \times 10^{-12}$ and 2.051×10^{-7}, respectively; Table 1). All five datasets contributed to this enrichment (Table 1; Tables S8 and S9), indicating that disruption of Arc complexes may contribute to a wide range of brain disorders. Consistent with the analysis of de novo CNVs, much of the enrichment in LoF and NS mutations was attributable to Arc interactors whose expression is altered in the $PSD95^{-/-}$ mouse. This suggests that it is postsynaptic Arc-PSD95 complexes and not cytoplasmic Arc complexes that are relevant to these disorders.

Arc Complexes in Normal Variation in Human Intelligence

Although the role in cognition for Arc, PSD95, and their interacting proteins is well established from studies of mutations in mice (Fernández et al., 2009; Fitzgerald et al., 2014; Husi et al., 2000; Komiyama et al., 2002; McCurry et al., 2010; Migaud et al., 1998; Nithianantharajah et al., 2013; Plath et al., 2006; Ryan et al., 2013), mutations in humans cause cognitive disorders, and enrichment analysis of Arc-interacting proteins for mammalian phenotype (MP) terms shows 48 enriched terms ($p < 0.01$) associated with abnormal synaptic and cognitive functions (Table S10), much less is known about the relevance to normal variation in human cognition. We therefore asked whether common genetic variation in Arc complexes was associated with common variation in general cognitive ability (known as intelligence or *g*) using the genome-wide association study (GWAS) on intelligence from the five cohorts ($n = 3,511$) that make up the Cognitive Aging in England and Scotland (CAGES) consortium (Davies et al., 2011; Hill et al., 2014). The five cohorts are the Lothian Birth Cohort of 1921 and 1936 (Deary et al., 2012), the Aberdeen Birth Cohort of 1936 (Whalley et al., 2011), and the Manchester and Newcastle Longitudinal Studies of Cognitive Aging (Rabbitt et al., 2004), which together consist of a total of 3,511 healthy middle- to old-aged individuals who all live independently in the community. The measure of general cognitive ability was taken from the GWAS previously conducted by Hill et al. (2014) (Supplemental Experimental Procedures). To determine whether there was a greater weight of evidence for association between the Arc gene set and general cognitive ability, a two-stage enrichment test was used. First, SNPs were assigned to autosomal genes, and a gene based statistic was derived (Liu et al., 2010). Second, the p values of the gene-based statistics were –log(10)-transformed before gene set enrichment analysis (GSEA) (Subramanian et al., 2005) and a competitive test of enrichment, was used. The results of the gene-based analysis are shown in Table S11, where eight genes were nominally significant in CAGES and nine in the Brisbane Adolescent Twin Study (BATS). The most significant gene in the BATS cohort (*PRRT1*, $p = 0.00732$) was also nominally significant in CAGES ($p = 0.03797$). The results of the enrichment analysis show that common genetic variation in Arc complex proteins shows nominally significant association ($p = 0.0473$) with intelligence compared with control gene sets. A replication study using the summary data of a GWAS conducted on intelligence (Hill et al., 2014), the BATS ($n = 2,062$; de Zubicaray et al., 2008; Wright et al., 2001; Wright and Martin, 2004), also showed a significant enrichment ($p = 0.0247$), confirming the results found in the CAGES consortium. This significant enrichment shows that common genetic variation in the genes encoding Arc complex proteins is associated with the normal variation in human intelligence differences.

DISCUSSION

We have developed and demonstrated an integrated proteomic and genetic strategy that reveals insights into Arc's role in biology, the synaptic basis of mental disorders, and intelligence. Multiple genetic and genome engineering methods were combined to isolate native Arc complexes, identify their constituents, determine the mechanism of assembly and localization to the postsynaptic terminal, and identify multiple diseases and mutations that converge on the complexes.

The Arc protein is principally housed within 1.5-MDa complexes, and proteomic MS identified many novel Arc-interacting proteins, of which PSD95 was the most abundant. PSD95 and Arc coassemble into 1.5-MDa supercomplexes, and knockout of PSD95 abolishes these complexes, severely depletes Arc from the postsynaptic terminal, and prevents its activity-dependent recruitment. The combined use of gene-tagged and mutant mice allowed us to dissect the interactions of Arc with specific subsets of postsynaptic complexes. PSD95 supercomplexes are a family of which ~3% contain NMDA receptors (Frank et al., 2017). The NMDA receptor requires PSD93 for coassembly with PSD95 (Frank et al., 2016), and in the present study, we found that PSD93 knockouts did not interfere with Arc-PSD95 interactions. Therefore, Arc can assemble with PSD95 supercomplexes that

Table 1. Arc Gene Set Analysis of Autism, Schizophrenia, Epilepsy, ID, and Schizophrenia Candidate Gene Sets

			All Arc Interactors		Arc Interactors with Increased Expression in PSD95/*Dlg4*$^{-/-}$ Mice		Arc Interactors with Decreased Expression in PSD95/*Dlg4*$^{-/-}$ Mice		Arc Interactors that Are Known Direct PSD95-Interacting Proteins	
			N = 107		N = 11		N = 24		N = 18	
Mutation Class	Disease	Study Design	P	O/E	P	O/E	P	O/E	P	O/E
CNVs	schizophrenia	de novo	0.00469	–	0.50211	–	0.01648	–	0.01648	–
LoF	combined	–	2.05E-07	–	1.00000	–	1.57E–06	–	3.03E–06	–
	autism	de novo	0.14352	11/4.0	1.00000	0/0.4	0.10920	6/1.2	0.00104	8/0.8
	epilepsy	de novo	0.39935	3/0.4	1.00000	0/0	1.00000	1/0.1	1.00000	0/0.1
	ID	de novo	0.00104	8/0.5	1.00000	0/0	0.00104	5/0.1	0.00936	3/0.1
	schizophrenia	de novo	1.00000	3/0.8	1.00000	1/0.1	1.00000	2/0.2	0.60527	2/0.2
	schizophrenia	case/control	1.00000	56/38	1.00000	12/9	1.00000	15/9	1.00000	6/6
NS	combined	–	9.01E–12	–	1.00000	–	1.59E–06	–	1.26E–08	–
	autism	de novo	0.01352	46/25.9	1.00000	2/2.5	1.00000	15/8.5	0.00416	18/5.8
	epilepsy	de novo	0.00104	16/2.5	1.00000	0/0.3	1.00000	3/0.8	1.00000	2/0.6
	ID	de novo	0.00104	15/2.0	1.00000	0/0.2	0.00104	8/0.6	0.00104	8/0.4
	schizophrenia	de novo	0.11856	14/5.7	1.00000	3/0.6	0.58759	6/1.8	0.47839	5/1.3
	schizophrenia	case/control	1.00000	187/184	1.00000	22/21	1.00000	61/48	0.72800	40/31

Shown are enrichment test empirical p values for autism (De Rubeis et al., 2014; Iossifov et al., 2012; Jiang et al., 2013), epilepsy (EuroEPINOMICS-RES Consortium et al., 2014), ID (de Ligt et al., 2012; Hamdan et al., 2014; Rauch et al., 2012), and schizophrenia (Fromer et al., 2014; Girard et al., 2011; Gulsuner et al., 2013; Kirov et al., 2012; McCarthy et al., 2014; Xu et al., 2012). For de novo studies, O/E indicates the observed versus expected (under a null model) number of mutations in that class. For the schizophrenia (SCZ) case/control study, O/E indicates the number of case versus control mutations. P indicates Bonferroni multiple-test correction: four tests for CNVs, 12 tests for Lof/NS combined analyses, and 52 tests for LoF/NS in individual studies. Fisher's method was used to combine p values from the five independent genetic datasets ("combined" p value for LoF and NS mutations). PSD95$^{-/-}$, PSD95/*Dlg4* knockout mice.

OPEN ACCESS CellPress

do not contain NMDA receptors. We also found that Arc did not interact with SAP102, which forms distinct complexes at ~350 kDa (Frank et al., 2016), nor did Arc require SAP102 for postsynaptic targeting. Together, these results demonstrate that Arc is targeted to the postsynaptic terminal, where it selectively interacts with signaling complexes organized by PSD95. Super-resolution microscopy has revealed that PSD95 and SAP102 are in separate nanodomains (Zheng et al., 2011) within the dendritic spine and that PSD95 nanodomains (Broadhead et al., 2016; Fukata et al., 2013; Nair et al., 2013) are positioned beneath the presynaptic release machinery (Tang et al., 2016). This suggests that Arc is selectively targeted by PSD95 to this critical region of the postsynaptic terminal, where its supercomplexes participate in controlling synaptic transmission and plasticity.

Disruption of many proteins in Arc-PSD95 complexes, and many other proteins in the supercomplexes leads to changes in synaptic plasticity and behavior, including knockout of Arc and PSD95, which both lead to enhanced LTP and impaired learning (Carlisle et al., 2008; Migaud et al., 1998; Nithianantharajah et al., 2013; Plath et al., 2006; N.H.K., L.N. van de Lagemaat, L.E. Stanford, C.M. Pettit, D.J. Strathdee, K.E. Strathdee, D.G.F., E.J. Tuck, K.A.E., T.J. Ryan, J.N., N.G. Skene, M.D.R.C., and S.G.N.G., unpublished data; M.V.K., L.N. van de Lagemaat, N. Afinowi, D.J. Strathdee, K.E. Strathdee, D.G.F., E.J. Tuck, K.A.E., N.G. Skene, M.D.R.C., N.H.K., and S.G.N.G., unpublished data). A recent large-scale genetic screen of postsynaptic proteins in mice showed that PSD95 supercomplexes were essential for the postsynaptic responses to simple and complex patterns of activity and the modulation of synaptic strength over a range of milliseconds to an hour (M.V.K., L.N. van de Lagemaat, N. Afinowi, D.J. Strathdee, K.E. Strathdee, D.G.F., E.J. Tuck, K.A.E., N.G. Skene, M.D.R.C., N.H.K., and S.G.N.G., unpublished data). The supercomplexes were also essential for tuning the magnitude of innate and learned behavioral responses, including simple and complex forms of behavior (N.H.K., L.N. van de Lagemaat, L.E. Stanford, C.M. Pettit, D.J. Strathdee, K.E. Strathdee, D.G.F., E.J. Tuck, K.A.E., T.J. Ryan, J.N., N.G. Skene, M.D.R.C., and S.G.N.G., unpublished data). Furthermore, these studies show that each innate and learned behavioral response required a specific subset or combination of postsynaptic proteins, which suggests that transient upregulation and targeting of Arc to PSD95 supercomplexes will transiently modify behavior and synaptic physiology. This mechanism is consistent with the known role of Arc in learning.

The proteomes of the post-synaptic density (PSD) and PSD95 supercomplexes are highly conserved between mice and humans (Bayés et al., 2011), and specific genes (e.g., PSD93) have conserved roles in cognition (visuo-spatial learning, cognitive flexibility, and attention) (Nithianantharajah et al., 2013). Our finding that human genetic disorders of cognition converge on Arc-PSD95 supercomplexes is in agreement with the mouse genetic findings. Here we have reaffirmed the role of the supercomplexes in schizophrenia and extended the study to autism and ID. Moreover, the finding that variation in normal human intelligence and disorders of cognition involves the same sets of proteins indicates that genetic variation in Arc-PSD95 supercomplexes underpins the phenotypic continuum between normal cognitive variation and pathology.

There are over 130 brain diseases linked to mutations in the postsynaptic proteome (Bayés et al., 2011) and a large number of uncharacterized multiprotein complexes (Frank et al., 2016), many of which contain at least one protein encoded by a disease gene. The integrated workflow shown here, which is centered on genetically tagged mice and proteomic approaches, offers a general and scalable approach toward understanding how the polygenic basis of brain disease is linked to the supramolecular organization of proteins in the postsynaptic terminal of central synapses. All datasets are freely available through the Genes to Cognition website (http://www.genes2cognition.org).

EXPERIMENTAL PROCEDURES

Animals

All animal experiments were conducted in a licensed animal facility in accordance to guidelines determined by the UK Animals (Scientific Procedures) Act, 1986 and approved through the U.K. Home Office Inspectorate. Animal care at KU Leuven was conducted according to national and international guidelines and as described in the Supplemental Experimental Procedures. All mice were 2- to 5-month-old males unless indicated otherwise.

TAP

TAP was performed as by Fernández et al. (2009). Briefly, mouse forebrain was homogenized on ice in 1% deoxycholate (DOC) buffer (50 mM Tris [pH 9.0], 1% sodium deoxycholate, 50 mM NaF, 20 μM $ZnCl_2$, and 1 mM Na_3VO_4), 2 mM Pefabloc SC (Roche), and 1 tablet/10 mL protease inhibitor cocktail tablets (Roche) at 0.38 g wet weight per 7 mL cold buffer with a glass Teflon Douncer homogenizer. The homogenate was incubated for 1 hr at 4°C and clarified at 50,000 × *g* for 30 min at 4°C. TAP-tagged complexes were isolated as described previously (Fernández et al., 2009). The SDS-PAGE gel was fixed and stained with colloidal Coomassie, and lanes were cut into slices, destained, and digested overnight with trypsin (Roche, trypsin modified, sequencing grade) as described previously (Fernández et al., 2009). Peptide digestion, LC-MS/MS, and proteomics data analysis are described in the Supplemental Experimental Procedures.

Enrichment Analysis of CNVs and Rare Coding Mutations in Arc Interactors in Human Neuropsychiatric Disease

The protein IDs from Table S1 were converted into both mouse genome informatics (MGI) and mouse NCBI/Entrez gene IDs using the online ID mapping tool provided by Uniprot and then converted to human Entrez IDs using the mapping file "HOM_MouseHumanSequence.rpt," available from MGI (http://www.informatics.jax.org/). Any genes with a non-unique (e.g., 1-many) mapping between species, or where MGI and mouse Entrez IDs mapped to different human genes, were excluded. De novo CNV enrichment analysis and de novo mutation exome sequencing datasets are detailed in the Supplemental Experimental Procedures.

Human Cognitive Ability Phenotype and Analysis

The phenotypes used in both the CAGES and the BATS samples were taken from the summary data of Hill et al. (2014) and are described in the Supplemental Experimental Procedures. Genome-wide association had been carried out in each cohort of CAGES using Mach2QTL (Li et al., 2010) before being meta-analyzed in METAL (Willer et al., 2010) and is detailed in the Supplemental Experimental Procedures.

All other methods are described in the Supplemental Experimental Procedures.

DATA AND SOFTWARE AVAILABILITY

The accession number for the mass spectrometry proteomics data reported in this paper is ProteomeXchange Consortium: PXD007283.

SUPPLEMENTAL INFORMATION

Supplemental Information includes Supplemental Experimental Procedures, one figure, and eleven tables and can be found with this article online at https://doi.org/10.1016/j.celrep.2017.09.045.

AUTHOR CONTRIBUTIONS

Conceptualization, E.F. and S.G.N.G.; Methodology, E.F., F.Z., and N.H.K.; Investigation, E.F., M.O.C., R.A.W.F., F.Z., J.N., S.A.L., and M.V.K.; Validation, E.F.; Disease Genetics, A.J.P., M.F., and S.M.P.; Cognition Genetics, W.D.H. and I.J.D.; Resources, J.S.C. and C.B.; Data Curation, M.D.R.C., C.M., and J.D.A.; Technical Assistance, D.F., K.A.E., C.L.M., and G.C.; Writing – Original Draft, E.F. and S.G.N.G.; Writing – Review & Editing, E.F., M.O.C., R.A.W.F., M.V.K., J.N., A.J.P., N.H.K., J.S.C., C.B., and S.G.N.G.; Project Management, Coordination, and Funding Acquisition, S.G.N.G.

ACKNOWLEDGMENTS

Funding was provided by the European Union Seventh Framework Programme under grant agreement HEALTH-F2-2009-242167 (“SynSys-project”) and HEALTH-F2-2009-241498 (“EUROSPIN” project), the Wellcome Trust, the Biotechnology and Biological Sciences Research Council (BBSRC), and the Medical Research Council (MRC). E.F. was supported by a Federation of European Biochemistry Societies postdoctoral fellowship, Marie Curie FP7-PEOPLE-2010-IEF, and the Department of Defense W81XWH-15-1-0361. C.B. was supported by VIB, the Department of Defense W81XWH-15-1-0361, and the Associazione Italiana Sindrome X Fragile. A.J.P. was funded by the Medical Research Council (MRC) Centre (MR/L010305/1) and Program Grants (G0801418). W.D.H. was supported by Age UK (Disconnected Mind) and BBSRC/MRC (MR/K026992/1). We thank D. Maizels for artwork. S.G.N.G. was a founder and shareholder of Synome Ltd. M.V.K. and D.F. were employees of Synome Ltd.

Received: August 26, 2015
Revised: August 3, 2017
Accepted: September 13, 2017
Published: October 17, 2017

REFERENCES

Babu, M., Arnold, R., Bundalovic-Torma, C., Gagarinova, A., Wong, K.S., Kumar, A., Stewart, G., Samanfar, B., Aoki, H., Wagih, O., et al. (2014). Quantitative genome-wide genetic interaction screens reveal global epistatic relationships of protein complexes in Escherichia coli. PLoS Genet. *10*, e1004120.

Bah, A., Vernon, R.M., Siddiqui, Z., Krzeminski, M., Muhandiram, R., Zhao, C., Sonenberg, N., Kay, L.E., and Forman-Kay, J.D. (2015). Folding of an intrinsically disordered protein by phosphorylation as a regulatory switch. Nature *519*, 106–109.

Bayés, A., van de Lagemaat, L.N., Collins, M.O., Croning, M.D., Whittle, I.R., Choudhary, J.S., and Grant, S.G. (2011). Characterization of the proteome, diseases and evolution of the human postsynaptic density. Nat. Neurosci. *14*, 19–21.

Bayés, A., Collins, M.O., Croning, M.D., van de Lagemaat, L.N., Choudhary, J.S., and Grant, S.G. (2012). Comparative study of human and mouse postsynaptic proteomes finds high compositional conservation and abundance differences for key synaptic proteins. PLoS ONE *7*, e46683.

Bayés, À., Collins, M.O., Galtrey, C.M., Simonnet, C., Roy, M., Croning, M.D., Gou, G., van de Lagemaat, L.N., Milward, D., Whittle, I.R., et al. (2014). Human post-mortem synapse proteome integrity screening for proteomic studies of postsynaptic complexes. Mol. Brain *7*, 88.

Bayés, À., Collins, M.O., Reig-Viader, R., Gou, G., Goulding, D., Izquierdo, A., Choudhary, J.S., Emes, R.D., and Grant, S.G. (2017). Evolution of complexity in the zebrafish synapse proteome. Nat. Commun. *8*, 14613.

Brandes, U., and Wagner, D., (2012). Visone, Analysis and visualization of social networks, version 2.6.5, University of Konstanz.

Broadhead, M.J., Horrocks, M.H., Zhu, F., Muresan, L., Benavides-Piccione, R., DeFelipe, J., Fricker, D., Kopanitsa, M.V., Duncan, R.R., Klenerman, D., et al. (2016). PSD95 nanoclusters are postsynaptic building blocks in hippocampus circuits. Sci. Rep. *6*, 24626.

Brose, N., O’Connor, V., and Skehel, P. (2010). Synaptopathy: dysfunction of synaptic function? Biochem. Soc. Trans. *38*, 443–444.

Carlisle, H.J., Fink, A.E., Grant, S.G., and O’Dell, T.J. (2008). Opposing effects of PSD-93 and PSD-95 on long-term potentiation and spike timing-dependent plasticity. J. Physiol. *586*, 5885–5900.

Chowdhury, S., Shepherd, J.D., Okuno, H., Lyford, G., Petralia, R.S., Plath, N., Kuhl, D., Huganir, R.L., and Worley, P.F. (2006). Arc/Arg3.1 interacts with the endocytic machinery to regulate AMPA receptor trafficking. Neuron *52*, 445–459.

Daberkow, D.P., Riedy, M.D., Kesner, R.P., and Keefe, K.A. (2007). Arc mRNA induction in striatal efferent neurons associated with response learning. Eur. J. Neurosci. *26*, 228–241.

Davies, G., Tenesa, A., Payton, A., Yang, J., Harris, S.E., Liewald, D., Ke, X., Le Hellard, S., Christoforou, A., Luciano, M., et al. (2011). Genome-wide association studies establish that human intelligence is highly heritable and polygenic. Mol. Psychiatry *16*, 996–1005.

de Ligt, J., Willemsen, M.H., van Bon, B.W., Kleefstra, T., Yntema, H.G., Kroes, T., Vulto-van Silfhout, A.T., Koolen, D.A., de Vries, P., Gilissen, C., et al. (2012). Diagnostic exome sequencing in persons with severe intellectual disability. N. Engl. J. Med. *367*, 1921–1929.

De Rubeis, S., He, X., Goldberg, A.P., Poultney, C.S., Samocha, K., Cicek, A.E., Kou, Y., Liu, L., Fromer, M., Walker, S., et al.; DDD Study; Homozygosity Mapping Collaborative for Autism; UK10K Consortium (2014). Synaptic, transcriptional and chromatin genes disrupted in autism. Nature *515*, 209–215.

de Zubicaray, G.I., Chiang, M.C., McMahon, K.L., Shattuck, D.W., Toga, A.W., Martin, N.G., Wright, M.J., and Thompson, P.M. (2008). Meeting the Challenges of Neuroimaging Genetics. Brain Imaging Behav. *2*, 258–263.

Deary, I.J., Gow, A.J., Pattie, A., and Starr, J.M. (2012). Cohort profile: the lothian birth cohorts of 1921 and 1936. Int. J. Epidemiol. *41*, 1576–1584.

Distler, U., Schmeisser, M.J., Pelosi, A., Reim, D., Kuharev, J., Weiczner, R., Baumgart, J., Boeckers, T.M., Nitsch, R., Vogt, J., and Tenzer, S. (2014). In-depth protein profiling of the postsynaptic density from mouse hippocampus using data-independent acquisition proteomics. Proteomics *14*, 2607–2613.

EuroEPINOMICS-RES Consortium; Epilepsy Phenome/Genome Project; Epi4K Consortium (2014). De novo mutations in synaptic transmission genes including DNM1 cause epileptic encephalopathies. Am. J. Hum. Genet. *95*, 360–370.

Fernández, E., Collins, M.O., Uren, R.T., Kopanitsa, M.V., Komiyama, N.H., Croning, M.D., Zografos, L., Armstrong, J.D., Choudhary, J.S., and Grant, S.G. (2009). Targeted tandem affinity purification of PSD-95 recovers core postsynaptic complexes and schizophrenia susceptibility proteins. Mol. Syst. Biol. *5*, 269.

Fitzgerald, P.J., Pinard, C.R., Camp, M.C., Feyder, M., Sah, A., Bergstrom, H.C., Graybeal, C., Liu, Y., Schlüter, O.M., Grant, S.G., et al. (2014). Durable fear memories require PSD-95. Mol. Psychiatry *20*, 901–912.

Frank, R.A., and Grant, S.G. (2017). Supramolecular organization of NMDA receptors and the postsynaptic density. Curr. Opin. Neurobiol. *45*, 139–147.

Frank, R.A., Komiyama, N.H., Ryan, T.J., Zhu, F., O’Dell, T.J., and Grant, S.G. (2016). NMDA receptors are selectively partitioned into complexes and supercomplexes during synapse maturation. Nat. Commun. *7*, 11264.

Frank, R.A.W., Zhu, F., Komiyama, N.H., and Grant, S.G.N. (2017). Hierarchical organization and genetically separable subfamilies of PSD95 postsynaptic supercomplexes. J. Neurochem. *142*, 504–511.

Fromer, M., Pocklington, A.J., Kavanagh, D.H., Williams, H.J., Dwyer, S., Gormley, P., Georgieva, L., Rees, E., Palta, P., Ruderfer, D.M., et al. (2014).

De novo mutations in schizophrenia implicate synaptic networks. Nature *506*, 179–184.

Fukata, Y., Dimitrov, A., Boncompain, G., Vielemeyer, O., Perez, F., and Fukata, M. (2013). Local palmitoylation cycles define activity-regulated post-synaptic subdomains. J. Cell Biol. *202*, 145–161.

Gao, M., Sossa, K., Song, L., Errington, L., Cummings, L., Hwang, H., Kuhl, D., Worley, P., and Lee, H.K. (2010). A specific requirement of Arc/Arg3.1 for visual experience-induced homeostatic synaptic plasticity in mouse primary visual cortex. J. Neurosci. *30*, 7168–7178.

Gibbs, E.B., Lu, F., Portz, B., Fisher, M.J., Medellin, B.P., Laremore, T.N., Zhang, Y.J., Gilmour, D.S., and Showalter, S.A. (2017). Phosphorylation induces sequence-specific conformational switches in the RNA polymerase II C-terminal domain. Nat. Commun. *8*, 15233.

Girard, S.L., Gauthier, J., Noreau, A., Xiong, L., Zhou, S., Jouan, L., Dionne-Laporte, A., Spiegelman, D., Henrion, E., Diallo, O., et al. (2011). Increased exonic de novo mutation rate in individuals with schizophrenia. Nat. Genet. *43*, 860–863.

Grant, S.G. (2012). Synaptopathies: diseases of the synaptome. Curr. Opin. Neurobiol. *22*, 522–529.

Grant, S.G.N. (2013). Synaptic Disease in Psychiatry. In Neurobiology of Mental Illness, J.D.B. Dennis S. Charney, Pamela Sklar, E.J. Nestler, ed. (Oxford: Oxford University Press).

Grant, S.G., Marshall, M.C., Page, K.L., Cumiskey, M.A., and Armstrong, J.D. (2005). Synapse proteomics of multiprotein complexes: en route from genes to nervous system diseases. Hum. Mol. Genet. *14 Spec. No. 2*, R225–R234.

Greer, P.L., Hanayama, R., Bloodgood, B.L., Mardinly, A.R., Lipton, D.M., Flavell, S.W., Kim, T.K., Griffith, E.C., Waldon, Z., Maehr, R., et al. (2010). The Angelman Syndrome protein Ube3A regulates synapse development by ubiquitinating arc. Cell *140*, 704–716.

Gulsuner, S., Walsh, T., Watts, A.C., Lee, M.K., Thornton, A.M., Casadei, S., Rippey, C., Shahin, H., Nimgaonkar, V.L., Go, R.C., et al.; Consortium on the Genetics of Schizophrenia (COGS); PAARTNERS Study Group (2013). Spatial and temporal mapping of de novo mutations in schizophrenia to a fetal prefrontal cortical network. Cell *154*, 518–529.

Guzowski, J.F., McNaughton, B.L., Barnes, C.A., and Worley, P.F. (1999). Environment-specific expression of the immediate-early gene Arc in hippocampal neuronal ensembles. Nat. Neurosci. *2*, 1120–1124.

Guzowski, J.F., Lyford, G.L., Stevenson, G.D., Houston, F.P., McGaugh, J.L., Worley, P.F., and Barnes, C.A. (2000). Inhibition of activity-dependent arc protein expression in the rat hippocampus impairs the maintenance of long-term potentiation and the consolidation of long-term memory. J. Neurosci. *20*, 3993–4001.

Hamdan, F.F., Srour, M., Capo-Chichi, J.M., Daoud, H., Nassif, C., Patry, L., Massicotte, C., Ambalavanan, A., Spiegelman, D., Diallo, O., et al. (2014). De novo mutations in moderate or severe intellectual disability. PLoS Genet. *10*, e1004772.

Hill, W.D., Davies, G., van de Lagemaat, L.N., Christoforou, A., Marioni, R.E., Fernandes, C.P.D., Liewald, D.C., Croning, M.D.R., Payton, A., Craig, L.C.A., et al. (2014). Human cognitive ability is influenced by genetic variation in components of postsynaptic signalling complexes assembled by NMDA receptors and MAGUK proteins. Transl. Psychiatry *4*, e341.

Husi, H., and Grant, S.G. (2001). Isolation of 2000-kDa complexes of N-methyl-D-aspartate receptor and postsynaptic density 95 from mouse brain. J. Neurochem. *77*, 281–291.

Husi, H., Ward, M.A., Choudhary, J.S., Blackstock, W.P., and Grant, S.G. (2000). Proteomic analysis of NMDA receptor-adhesion protein signaling complexes. Nat. Neurosci. *3*, 661–669.

Iossifov, I., Ronemus, M., Levy, D., Wang, Z., Hakker, I., Rosenbaum, J., Yamrom, B., Lee, Y.H., Narzisi, G., Leotta, A., et al. (2012). De novo gene disruptions in children on the autistic spectrum. Neuron *74*, 285–299.

Jakkamsetti, V., Tsai, N.P., Gross, C., Molinaro, G., Collins, K.A., Nicoletti, F., Wang, K.H., Osten, P., Bassell, G.J., Gibson, J.R., and Huber, K.M. (2013). Experience-induced Arc/Arg3.1 primes CA1 pyramidal neurons for metabotropic glutamate receptor-dependent long-term synaptic depression. Neuron *80*, 72–79.

Jiang, Y.H., Yuen, R.K., Jin, X., Wang, M., Chen, N., Wu, X., Ju, J., Mei, J., Shi, Y., He, M., et al. (2013). Detection of clinically relevant genetic variants in autism spectrum disorder by whole-genome sequencing. Am. J. Hum. Genet. *93*, 249–263.

Kelly, M.P., and Deadwyler, S.A. (2003). Experience-dependent regulation of the immediate-early gene arc differs across brain regions. J. Neurosci. *23*, 6443–6451.

Kerrigan, T.L., and Randall, A.D. (2013). A new player in the "synaptopathy" of Alzheimer's disease - arc/arg 3.1. Front. Neurol. *4*, 9.

Kirov, G., Pocklington, A.J., Holmans, P., Ivanov, D., Ikeda, M., Ruderfer, D., Moran, J., Chambert, K., Toncheva, D., Georgieva, L., et al. (2012). De novo CNV analysis implicates specific abnormalities of postsynaptic signalling complexes in the pathogenesis of schizophrenia. Mol. Psychiatry *17*, 142–153.

Komiyama, N.H., Watabe, A.M., Carlisle, H.J., Porter, K., Charlesworth, P., Monti, J., Strathdee, D.J., O'Carroll, C.M., Martin, S.J., Morris, R.G., et al. (2002). SynGAP regulates ERK/MAPK signaling, synaptic plasticity, and learning in the complex with postsynaptic density 95 and NMDA receptor. J. Neurosci. *22*, 9721–9732.

Korb, E., Wilkinson, C.L., Delgado, R.N., Lovero, K.L., and Finkbeiner, S. (2013). Arc in the nucleus regulates PML-dependent GluA1 transcription and homeostatic plasticity. Nat. Neurosci. *16*, 874–883.

Li, L., Carter, J., Gao, X., Whitehead, J., and Tourtellotte, W.G. (2005). The neuroplasticity-associated arc gene is a direct transcriptional target of early growth response (Egr) transcription factors. Mol. Cell. Biol. *25*, 10286–10300.

Li, Y., Willer, C.J., Ding, J., Scheet, P., and Abecasis, G.R. (2010). MaCH: using sequence and genotype data to estimate haplotypes and unobserved genotypes. Genet. Epidemiol. *34*, 816–834.

Link, W., Konietzko, U., Kauselmann, G., Krug, M., Schwanke, B., Frey, U., and Kuhl, D. (1995). Somatodendritic expression of an immediate early gene is regulated by synaptic activity. Proc. Natl. Acad. Sci. USA *92*, 5734–5738.

Liu, J.Z., McRae, A.F., Nyholt, D.R., Medland, S.E., Wray, N.R., Brown, K.M., Hayward, N.K., Montgomery, G.W., Visscher, P.M., Martin, N.G., and Macgregor, S.; AMFS Investigators (2010). A versatile gene-based test for genome-wide association studies. Am. J. Hum. Genet. *87*, 139–145.

Lu, X., Kensche, P.R., Huynen, M.A., and Notebaart, R.A. (2013). Genome evolution predicts genetic interactions in protein complexes and reveals cancer drug targets. Nat. Commun. *4*, 2124.

Lyford, G.L., Yamagata, K., Kaufmann, W.E., Barnes, C.A., Sanders, L.K., Copeland, N.G., Gilbert, D.J., Jenkins, N.A., Lanahan, A.A., and Worley, P.F. (1995). Arc, a growth factor and activity-regulated gene, encodes a novel cytoskeleton-associated protein that is enriched in neuronal dendrites. Neuron *14*, 433–445.

Managò, F., Mereu, M., Mastwal, S., Mastrogiacomo, R., Scheggia, D., Emanuele, M., De Luca, M.A., Weinberger, D.R., Wang, K.H., and Papaleo, F. (2016). Genetic Disruption of Arc/Arg3.1 in Mice Causes Alterations in Dopamine and Neurobehavioral Phenotypes Related to Schizophrenia. Cell Rep. *16*, 2116–2128.

McCarthy, S.E., Gillis, J., Kramer, M., Lihm, J., Yoon, S., Berstein, Y., Mistry, M., Pavlidis, P., Solomon, R., Ghiban, E., et al. (2014). De novo mutations in schizophrenia implicate chromatin remodeling and support a genetic overlap with autism and intellectual disability. Mol. Psychiatry *19*, 652–658.

McCurry, C.L., Shepherd, J.D., Tropea, D., Wang, K.H., Bear, M.F., and Sur, M. (2010). Loss of Arc renders the visual cortex impervious to the effects of sensory experience or deprivation. Nat. Neurosci. *13*, 450–457.

Migaud, M., Charlesworth, P., Dempster, M., Webster, L.C., Watabe, A.M., Makhinson, M., He, Y., Ramsay, M.F., Morris, R.G., Morrison, J.H., et al. (1998). Enhanced long-term potentiation and impaired learning in mice with mutant postsynaptic density-95 protein. Nature *396*, 433–439.

Miyashita, T., Kubik, S., Haghighi, N., Steward, O., and Guzowski, J.F. (2009). Rapid activation of plasticity-associated gene transcription in hippocampal

neurons provides a mechanism for encoding of one-trial experience. J. Neurosci. *29*, 898–906.

Moga, D.E., Calhoun, M.E., Chowdhury, A., Worley, P., Morrison, J.H., and Shapiro, M.L. (2004). Activity-regulated cytoskeletal-associated protein is localized to recently activated excitatory synapses. Neuroscience *125*, 7–11.

Nair, D., Hosy, E., Petersen, J.D., Constals, A., Giannone, G., Choquet, D., and Sibarita, J.B. (2013). Super-resolution imaging reveals that AMPA receptors inside synapses are dynamically organized in nanodomains regulated by PSD95. J. Neurosci. *33*, 13204–13224.

Nithianantharajah, J., Komiyama, N.H., McKechanie, A., Johnstone, M., Blackwood, D.H., St Clair, D., Emes, R.D., van de Lagemaat, L.N., Saksida, L.M., Bussey, T.J., and Grant, S.G. (2013). Synaptic scaffold evolution generated components of vertebrate cognitive complexity. Nat. Neurosci. *16*, 16–24.

Okuno, H., Akashi, K., Ishii, Y., Yagishita-Kyo, N., Suzuki, K., Nonaka, M., Kawashima, T., Fujii, H., Takemoto-Kimura, S., Abe, M., et al. (2012). Inverse synaptic tagging of inactive synapses via dynamic interaction of Arc/Arg3.1 with CaMKIIβ. Cell *149*, 886–898.

Park, S., Park, J.M., Kim, S., Kim, J.A., Shepherd, J.D., Smith-Hicks, C.L., Chowdhury, S., Kaufmann, W., Kuhl, D., Ryazanov, A.G., et al. (2008). Elongation factor 2 and fragile X mental retardation protein control the dynamic translation of Arc/Arg3.1 essential for mGluR-LTD. Neuron *59*, 70–83.

Plath, N., Ohana, O., Dammermann, B., Errington, M.L., Schmitz, D., Gross, C., Mao, X., Engelsberg, A., Mahlke, C., Welzl, H., et al. (2006). Arc/Arg3.1 is essential for the consolidation of synaptic plasticity and memories. Neuron *52*, 437–444.

Pocklington, A.J., Cumiskey, M., Armstrong, J.D., and Grant, S.G. (2006). The proteomes of neurotransmitter receptor complexes form modular networks with distributed functionality underlying plasticity and behaviour. Mol. Syst. Biol. *2*, 2006.0023.

Purcell, S.M., Moran, J.L., Fromer, M., Ruderfer, D., Solovieff, N., Roussos, P., O'Dushlaine, C., Chambert, K., Bergen, S.E., Kähler, A., et al. (2014). A polygenic burden of rare disruptive mutations in schizophrenia. Nature *506*, 185–190.

Rabbitt, P.M.A., McInnes, L., Diggle, P., Holland, F., Bent, N., Abson, V., Pendleton, N., and Horan, M. (2004). The University of Manchester Longitudinal Study of Cognition in Normal Healthy Old Age, 1983 through 2003. Neuropsychol. Dev. Cogn. B. Aging Neuropsychol. Cogn. *11*, 245–279.

Rauch, A., Wieczorek, D., Graf, E., Wieland, T., Endele, S., Schwarzmayr, T., Albrecht, B., Bartholdi, D., Beygo, J., Di Donato, N., et al. (2012). Range of genetic mutations associated with severe non-syndromic sporadic intellectual disability: an exome sequencing study. Lancet *380*, 1674–1682.

Rial Verde, E.M., Lee-Osbourne, J., Worley, P.F., Malinow, R., and Cline, H.T. (2006). Increased expression of the immediate-early gene arc/arg3.1 reduces AMPA receptor-mediated synaptic transmission. Neuron *52*, 461–474.

Ryan, T.J., Emes, R.D., Grant, S.G., and Komiyama, N.H. (2008). Evolution of NMDA receptor cytoplasmic interaction domains: implications for organisation of synaptic signalling complexes. BMC Neurosci. *9*, 6.

Ryan, T.J., Kopanitsa, M.V., Indersmitten, T., Nithianantharajah, J., Afinowi, N.O., Pettit, C., Stanford, L.E., Sprengel, R., Saksida, L.M., Bussey, T.J., et al. (2013). Evolution of GluN2A/B cytoplasmic domains diversified vertebrate synaptic plasticity and behavior. Nat. Neurosci. *16*, 25–32.

Schwanhäusser, B., Busse, D., Li, N., Dittmar, G., Schuchhardt, J., Wolf, J., Chen, W., and Selbach, M. (2011). Global quantification of mammalian gene expression control. Nature *473*, 337–342.

Shepherd, J.D., Rumbaugh, G., Wu, J., Chowdhury, S., Plath, N., Kuhl, D., Huganir, R.L., and Worley, P.F. (2006). Arc/Arg3.1 mediates homeostatic synaptic scaling of AMPA receptors. Neuron *52*, 475–484.

Steward, O., Wallace, C.S., Lyford, G.L., and Worley, P.F. (1998). Synaptic activation causes the mRNA for the IEG Arc to localize selectively near activated postsynaptic sites on dendrites. Neuron *21*, 741–751.

Subramanian, A., Tamayo, P., Mootha, V.K., Mukherjee, S., Ebert, B.L., Gillette, M.A., Paulovich, A., Pomeroy, S.L., Golub, T.R., Lander, E.S., and Mesirov, J.P. (2005). Gene set enrichment analysis: a knowledge-based approach for interpreting genome-wide expression profiles. Proc. Natl. Acad. Sci. USA *102*, 15545–15550.

Tang, A.H., Chen, H., Li, T.P., Metzbower, S.R., MacGillavry, H.D., and Blanpied, T.A. (2016). A trans-synaptic nanocolumn aligns neurotransmitter release to receptors. Nature *536*, 210–214.

Vazdarjanova, A., and Guzowski, J.F. (2004). Differences in hippocampal neuronal population responses to modifications of an environmental context: evidence for distinct, yet complementary, functions of CA3 and CA1 ensembles. J. Neurosci. *24*, 6489–6496.

Vazdarjanova, A., Ramirez-Amaya, V., Insel, N., Plummer, T.K., Rosi, S., Chowdhury, S., Mikhael, D., Worley, P.F., Guzowski, J.F., and Barnes, C.A. (2006). Spatial exploration induces ARC, a plasticity-related immediate-early gene, only in calcium/calmodulin-dependent protein kinase II-positive principal excitatory and inhibitory neurons of the rat forebrain. J. Comp. Neurol. *498*, 317–329.

Vidal, M., Cusick, M.E., and Barabási, A.L. (2011). Interactome networks and human disease. Cell *144*, 986–998.

Wang, K.H., Majewska, A., Schummers, J., Farley, B., Hu, C., Sur, M., and Tonegawa, S. (2006). In vivo two-photon imaging reveals a role of arc in enhancing orientation specificity in visual cortex. Cell *126*, 389–402.

Waung, M.W., Pfeiffer, B.E., Nosyreva, E.D., Ronesi, J.A., and Huber, K.M. (2008). Rapid translation of Arc/Arg3.1 selectively mediates mGluR-dependent LTD through persistent increases in AMPAR endocytosis rate. Neuron *59*, 84–97.

Whalley, L.J., Murray, A.D., Staff, R.T., Starr, J.M., Deary, I.J., Fox, H.C., Lemmon, H., Duthie, S.J., Collins, A.R., and Crawford, J.R. (2011). How the 1932 and 1947 mental surveys of Aberdeen schoolchildren provide a framework to explore the childhood origins of late onset disease and disability. Maturitas *69*, 365–372.

Wibrand, K., Pai, B., Siripornmongcolchai, T., Bittins, M., Berentsen, B., Ofte, M.L., Weigel, A., Skaftnesmo, K.O., and Bramham, C.R. (2012). MicroRNA regulation of the synaptic plasticity-related gene Arc. PLoS ONE *7*, e41688.

Willer, C.J., Li, Y., and Abecasis, G.R. (2010). METAL: fast and efficient meta-analysis of genomewide association scans. Bioinformatics *26*, 2190–2191.

Wright, M.J., and Martin, N.G. (2004). The Brisbane Adolescent Twin Study: outline of study methods and research projects. Aust. J. Psychol. *56*, 65–78.

Wright, M., De Geus, E., Ando, J., Luciano, M., Posthuma, D., Ono, Y., Hansell, N., Van Baal, C., Hiraishi, K., Hasegawa, T., et al. (2001). Genetics of cognition: outline of a collaborative twin study. Twin Res. *4*, 48–56.

Xu, B., Ionita-Laza, I., Roos, J.L., Boone, B., Woodrick, S., Sun, Y., Levy, S., Gogos, J.A., and Karayiorgou, M. (2012). De novo gene mutations highlight patterns of genetic and neural complexity in schizophrenia. Nat. Genet. *44*, 1365–1369.

Xue, B., Dunbrack, R.L., Williams, R.W., Dunker, A.K., and Uversky, V.N. (2010). PONDR-FIT: a meta-predictor of intrinsically disordered amino acids. Biochim. Biophys. Acta *1804*, 996–1010.

Zhang, W., Wu, J., Ward, M.D., Yang, S., Chuang, Y.A., Xiao, M., Li, R., Leahy, D.J., and Worley, P.F. (2015). Structural basis of arc binding to synaptic proteins: implications for cognitive disease. Neuron *86*, 490–500.

Zheng, C.Y., Wang, Y.X., Kachar, B., and Petralia, R.S. (2011). Differential localization of SAP102 and PSD-95 is revealed in hippocampal spines using super-resolution light microscopy. Commun. Integr. Biol. *4*, 104–105.

Cell Reports, Volume *21*

Supplemental Information

Arc Requires PSD95 for Assembly into Postsynaptic Complexes Involved with Neural Dysfunction and Intelligence

Esperanza Fernández, Mark O. Collins, René A.W. Frank, Fei Zhu, Maksym V. Kopanitsa, Jess Nithianantharajah, Sarah A. Lemprière, David Fricker, Kathryn A. Elsegood, Catherine L. McLaughlin, Mike D.R. Croning, Colin Mclean, J. Douglas Armstrong, W. David Hill, Ian J. Deary, Giulia Cencelli, Claudia Bagni, Menachem Fromer, Shaun M. Purcell, Andrew J. Pocklington, Jyoti S. Choudhary, Noboru H. Komiyama, and Seth G.N. Grant

Supplemental Figure 1

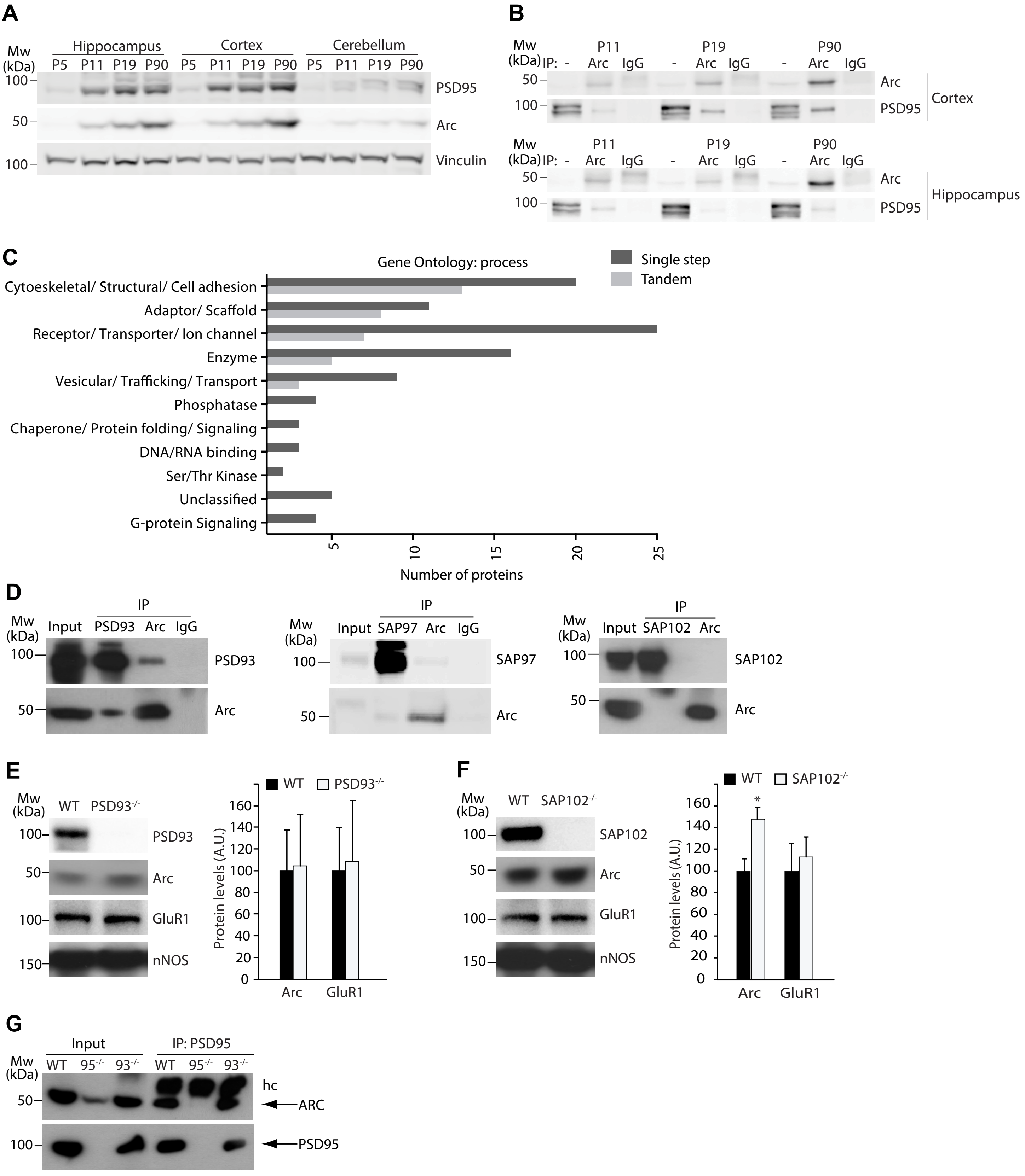

Figure S1. Selective interaction of MAGUK proteins with Arc complexes, Related to Figure 2. (A) PSD95 and Arc expression in hippocampus, cortex and cerebellum from wild type mice at postnatal days 5, 11, 19 and 90 (P5, P11, P19 and P90, respectively). Vinculin was used as loading control. **(B)** Arc was specifically purified with an antibody anti-Arc from hippocampal and cortical extracts at the developmental stages indicated. Immunoprecipitated complexes were blotted against Arc and PSD95. (**C**) Bar diagram with the number of proteins purified in the single step and the tandem purifications according to their gene ontology identifier. (**D**) Arc and the MAGUKs PSD93, PSD97 and SAP102 were purified with specific antibodies from forebrain WT extracts and blotted against Arc and PSD93 (lef panel), PSD97 (middle panel) and SAP102 (right panel) antibodies. IgG, mouse total IgGs; Mw: molecular weight in kDa. **(E)** and **(F)** Representative immunoblots of N = 4 independent animals, showing relative abundance of different proteins in lysates of hippocampus from PSD93 (**E**) and SAP102 (**F**) knockout mice and matched wild type littermates. Western blots were probed with antibodies against SAP102, PSD93, Arc, GluR1, and nNOS as indicated. Quantification of protein expression was calculated to the amount of nNOS in each sample and expressed as a percentage of the WT expression. N= 4 for each genotype. * $P < 0.05$, Mann-Whitney *U* test. (**G**) Arc interaction to PSD95 is independent of PSD93. PSD95 was immunoprecipitated from PSD95 and PSD93 mutant and WT mice forebrain extracts and immunoblotted against Arc and PSD95. Representative blot of n = 3 independent experiments. IP: antibodies used for immunoprecipitation, hc: Antibody heavy chain. -/-: knockout mice.

Supplemental Experimental Procedures

1. Materials

Sodium deoxycholate, 3-[(3-cholamidopropyl)dimethylammonio]-1- propanesulfonate, n-Dodecyl-N,N-Dimethyl-3-Ammonio-1-Propanesulfonat, β-D-maltopyranoside, N,N'-bis-(3-D-gluconamidopropyl) deoxycholamide (Sigma); anti-Arc (Santa Cruz Biotechnologies; C7); anti-Arc (Santa Cruz Biotechnologies; H300), anti-PSD95 (Thermo Scientific; 6G6-1C9), anti-Synaptophysin (EMD Millipore; 573822), anti-GluR1 (EMD Millipore; AB1504), anti-Dynamin-3, (BD-Biosciences; 610246), anti-actin (Sigma; A5228), anti-nNOS (EMD Millipore; AB1632), anti-SAP102 (NeuroMab; 75058), anti-PSD93 (NeuroMab; 75-057), anti-SAP97 (BD Biosciences; 610874/5), secondary antibodies conjugated with Alexa Fluor dyes (Invitrogen), biotinylated anti-mouse secondary antibody (1:200; DAKO; E0413) and peroxidase-linked secondary IgGs (EMD Millipore; 12-348 and -349).

2. Animals

Animal care at KU Leuven was conducted according to national and international guidelines (Belgian law of August 14th, 1986, and the following K.B. of November 14th, 1993 and K.B of September 13th, 2004; European Community Council Directive 86/609, OJa L 358, 1, December 12, 1987; National Institutes of Health Guide for the Care and Use of Laboratory Animals, National Research Council, 1996). Animals were exposed to conventional 12:12h light/dark cycles and ad libitum food supply. Mice were sacrificed by cervical dislocation and the forebrain was dissected on ice and snap-frozen in liquid nitrogen. Brain samples were stored at -80 ºC for few weeks prior to use.

3. Generation of TAP and Venus tagged Arc vectors and gene targeting.

The TAP tag fragment containing the Histidine Affinity Tag (HAT), TEV protease and Flag sequences flanked by XbaI and BclI restriction sites was cloned into pneoflox vector (TAPtag pneoflox) as described previously (Fernandez et al., 2009). Two homology arms of the genomic Arc/Arg3.1 sequence were amplified with forward ArcHAXhoIF and reverse ArcHAXbaIR primers and forward ArcHAAcc65IF and reverse ArcHABglIIR primers using the BAC bMQ101B9 as a template. Both homology arms were cloned into the TAPtag pneoflox vector leaving in between the TAP tag sequence, 2 loxP sites, PGK and EM7 promoters, the G418^r gene and an SV40 polyadenylation site. To generate the Venus cassette, the coding sequence of the yellow fluorescent protein (YFP) variant Venus was amplified by PCR with an artificial linker encoding for Gly-Gly-Gly-Ser at its 5' end with the primers VenusF and VenusR. Both *pneoflox-ArcTap* vector and Venus PCR products were digested with XbaI and BclI and Venus directly ligated to the vector, resulting on *pneoflox-ArcHA12-Venus* vector.
The cassettes of both targeting vectors flanked by two homology arms were removed and transformed into *EL350 E. coli* cells containing a pTargeter vector with the genomic Arc/Arg3.1 sequence cr1574496136 to cr15 74506490 (Ensembl release 49). The cassettes were inserted into the pTargeter vector by recombination (Liu et al., 2003). The final vectors contained a 5' -end homolog Arc sequence of 6664bp and a 3' -end homology arm of 3688bp (ENSMUSG00000022602). The vectors were linearized with PvuI and electroporated into E14 embryonic stem cells. The neomycin resistant colonies were picked up, expanded and frozen. Genomic DNA was extracted from all of them and PCR with and pneoF3 and Arc4R to identify TAP Arc homologous recombinants and with pneoF3 and Arc4R to identify Venus Arc recombinants.

4. Arc^{TAP} and Arc^{Venus} transgenic mice generation

One of the ES cells positive clones for each recombinant was microinjected into C57BL/6 blastocysts and 4 germline chimaeras were generated containing 30-90% of targeted cells. These chimaeras were crossed onto the C57BL/6 genetic background. Tail DNA from the litters was extracted and analyzed by PCR with a 5' Arc1F forward primer and two 3' pneo3R and Arc10R reverse primers to distinguish the heterozygous (+/-) and wild type (+/+) TAP Arc alleles, respectively, and with the primers NeoF1 and ArcExonF2 forward primers and ArcUTRR2 reverse primer to distinguish the heterozygous (+/-) and wild type (+/+) Venus alleles, respectively. Both mouse strains were bred with Cre-recombinase expressing transgenic mice to remove the Neo^r cassette. The knockin strains are referred as Arc^{TAP} and Arc-Venus. Both strains did not show any distortion of transmission frequency in the offspring of the intercrosses. TAP tagged Arc protein levels were similar to wild type Arc levels in forebrain extracts of Arc$^{TAP/+}$ mice and TAP tagged Arc was specifically immunoprecipitated by specific anti-Arc and anti-FLAG antibodies (Figure 1D).

5. Proteomics

Peptides were extracted from gel bands twice with 50% acetonitrile/0.5% formic acid and dried in a SpeedVac (Thermo). Peptides were resuspended using 0.5% formic acid analyzed using an Ultimate 3000 Nano/Capillary LC System (Dionex) coupled to either an LTQ FT Ultra (for characterization of TAP tagged Arc complexes) or an LTQ Orbitrap Velos (differential analysis of TAP tagged Arc complexes in WT or PSD95 mutants) hybrid mass spectrometers (Thermo Electron) equipped with a nanospray ion source. Peptides were desalted on-line using a micro-Precolumn cartridge (C18 Pepmap 100, LC Packings) and then separated using either a 35 min RP gradient (4-32% acetonitrile/0.1% formic acid) on a BEH C18 analytical column (1.7 μm, 75 μm id x 10 cm,) (Waters) (for characterization of TAP tagged Arc complexes) or a 125 min RP gradient (4-32% acetonitrile/0.1% formic acid) on an Acclaim PepMap100 C18 analytical column (3 μm, 75 μm id x 50 cm,) (Dionex) (differential analysis of TAP tagged Arc complexes in WT or PSD95 mutants). The mass spectrometer was operated in standard data dependent acquisition mode controlled by Xcalibur 2.0/2.1. The LTQ-FT Ultra was operated with a cycle of one MS (in

the FTICR cell) acquired at a resolution of 100,000 at m/z 400, with the top five most abundant multiply-charged (2+ and higher) ions in a given chromatographic window subjected to MS/MS fragmentation in the linear ion trap. The LTQ-Orbitrap Velos was operated with a cycle of one MS (in the Orbitrap) acquired at a resolution of 60,000 at m/z 400, with the top 20 most abundant multiply-charged (2+ and higher) ions in a given chromatographic window subjected to MS/MS fragmentation in the linear ion trap. FTMS target values of 5e5 (LTQ-FT Ultra) and 1e6 (LTQ-Orbitrap Velos) and an ion trap MSn target values of 1e4 were used. Maximum FTMS scan accumulation times were set at 1000ms (LTQ-FT Ultra) and 500ms (LTQ-Orbitrap Velos) and maximum ion trap MSn scan accumulation tiles were set to 200ms (LTQ-FT Ultra) and 100ms (LTQ-Orbitrap Velos). Dynamic exclusion was enabled with a repeat duration of 45s with an exclusion list of 500 and exclusion duration of 30s.

6. Proteomic data analysis

Data was analyzed data using MaxQuant version 1.3.0.5 (Cox and Mann, 2008). MaxQuant processed data was searched against a UniProt mouse proteome sequence database (Mar, 2012) using the following search parameters: trypsin with a maximum of 2 missed cleavages, 7 ppm for MS mass tolerance, 0.5 Da for MS/MS mass tolerance, with Acetyl (Protein N-term) and Oxidation (M) set as variable modifications and carbamidomethyl (C) as a fixed modification. A protein FDR of 0.01 and a peptide FDR of 0.01 were used for identification level cut offs. Protein quantification was performed using razor and unique peptides and using only unmodified and carbamidomethylated peptides.

Summary of one-step purifications

Two sets of one-step purifications:
3 replicates of $ARC^{TAP/TAP}$ and WT (as control) purifications dimethyl labelled for quantification by MaxQuant).
2 replicates of $ARC^{TAP/TAP}$ and 1 control (MaxQuant intensity-based label free quantification)
Proteins were considered enriched if they had a ratio of enrichment of 2.5 ($ARC^{TAP/TAP}$/Control purification) in two out of 5 one-step purifications.

Summary of tandem purifications

Three replicates of $ARC^{TAP/TAP}$ and control purifications (MaxQuant intensity-based label free quantification). Proteins were considered enriched if they had a ratio of enrichment of 2.5 ($ARC^{TAP/TAP}$/Control purification) in two out of three two-step purifications. The total set of 107 ARC complex components was assembled from the combined set of enriched proteins from one step and tandem purifications.

Summary of purification from $ARC^{TAP/TAP}$/$PSD95^{-/-}$ mice

Arc binding partners were captured from lysed forebrain from $Arc^{TAP/TAP}$x$PSD95^{-/-}$. In order to isolate similar amounts of Arc-associated protein complexes from WT and $PSD95^{-/-}$, TAP tagged Arc was captured from heterozygous $Arc^{TAP/+}$ mice. The TAP procedure, concentration of the eluted samples and gel staining were performed as described in Experimental procedures. The amount of protein lysed, captured and eluted was monitored by semiquantitative immunoblotting before LC-MS/MS (Figure 5A).
Three replicate one-step purifications of ARC complexes from $ARC^{TAP/+}$, $ARC^{TAP/TAP}$/$PSD95^{-/-}$ and control mice were dimethyl labelled for quantification by MaxQuant. Given that ARC expression is down-regulated in PSD-95 mutant mice, all protein ratios were normalized to that determined for ARC by MaxQuant. 77 out of the total set of 107 ARC complex proteins were quantified in dimethyl labelled PSD95 mutant versus wild type experiments and 59 of these proteins could be quantified in at least 2 out of 3 replicates. Of these, 24 were decreased by 1.5-fold in the PSD-95 mutant (Ratio < 0.67) and 11 showed a 1.5 fold increase in abundance in the PSD95 mutant (Ratio >1.5).

7. Blue Native PAGE

Blue-native PAGE has been performed as described in (Frank et al., 2016). Briefly, forebrains (hippocampus and cortex) were dissected from adult (P56-70) mice and were homogenized (12 strokes with a Teflon-glass pestle and mortar) in 5 ml ice-cold buffer H (1 mM Na HEPES pH7.4, 320 mM sucrose with protease inhibitors). The homogenate pellet was collected by centrifugation with 1,000 x*g*. at 2°C for 10 min was re-homogenized (6 strokes) in 2 ml buffer H and centrifuged as before. The first and second 1000 x*g*. supernatants were pooled and centrifuged at 18500 x*g*. to pellet the crude membranes. The 18500 x*g*. supernatant was discarded. Extraction conditions were screened using the crude membrane from 50-60 mg mouse forebrain re-suspended in 0.5 ml buffer H, to which different detergents sodium deoxycholate, 3-[(3-cholamidopropyl)dimethylammonio]-1-propanesulfonate, n-Dodecyl-N,N-Dimethyl-3-Ammonio-1-Propanesulfonat, β-D-maltopyranoside, N,N'-bis-(3-D-gluconamidopropyl) deoxycholamide in 0.5 ml buffer X (100-300 mM NaCl, 50 mM tris.Cl pH8) were mixed for 1 h at 6-10°C. Insoluble proteins were removed from the total extract by centrifugation at 120,000 x*g*. for 40 min at 8°C. BNPs were immediately run and immunoblotted to detect the native complexes of Arc, PSD95 and PSD93.

8. Cellular fractionation

Brain cellular fractionation was done according to the protocol described earlier (Chowdhury et al., 2006). Mouse forebrains were homogenized in 10 volumes of sucrose buffer (0.32 M sucrose, 4 mM HEPES, pH 7.4, 1 mM EDTA, 1 mM EGTA and protease inhibitors cocktail tablets (Roche)) with a glass Teflon Dounce homogenizer, and centrifuged at 800 g for 15 min. The

supernatant (SN1) was centrifuged 15 min at 9000 g and the pellet collected as crude membrane fraction (P2). The supernatant (SN2) containing the cytosolic fraction was again centrifuged for 60 min at 100,000g. The pellet was collected as microsomal fraction (P4) and the supernatant (SN4) was considered as the cytoskeletal fraction. P2 crude membrane fraction containing synaptosomes was solubilized in 0.2% DOC buffer in the presence of protease inhibitors and centrifuged for 20 min at 100,000g. Pellet (P3) was considered as PSD-enriched fraction and supernatant (SN3) was considered as presynaptic fraction. Fractions were stained with antibodies against PSD95 (Thermo Scientific; 6G6-1C9), Arc (Santa Cruz Biotechnologies; H300), Synaptophysin (EMD Millipore; 573822), and actin (Sigma; A5228).

9. Immunostaining of brain sections and primary neurons
TAP tagged Arc showed a similar distribution pattern to wild type Arc (Steward et al., 1998) in dendrites of hippocampal CA1, CA3 areas and dentate gyrus (Figure 1E) Mice were anesthetized with a mix of ketamine/xylazine and intracardially perfused with 0.1 M sodium phosphate buffer (pH 7.4) followed by 4 % paraformaldehyde in 0.1M sodium phosphate buffer (pH 7.4). The brain was removed and placed in a fixative for a further 1 h and then equilibrated overnight in 30 % sucrose in PBS. Free-floating sections were incubated with anti-Arc (1:250; Santa Cruz Biotechnologies; C7) and biotinylated anti-mouse secondary antibody (1:200; DAKO; E0413) for peroxidase staining. The signal was amplified using an avidin-biotin system (Vector Laboratories) followed by a staining in 3,3-diaminobenzidine (Sigma).
TAP tagged Arc showed a similar subcellular distribution as wild type Arc being partly co-localized with PSD95 and synaptophysin in synaptic boutons. Hippocampi were dissected from E15.5 mouse embryos of wild type (WT) and $Arc^{TAP/TAP}$ C57Bl/6 littermates and digested in papain to yield suspension of primary neurons (Figure 1F). The culture conditions and the immnocytochemistry protocol were performed as described earlier (Valor et al., 2007). Primary neurons were fixed and stained at 14 days *in vitro* (DIV) and labeled with the following antibodies: rabbit Arc 1/250 (Santa Cruz Biotechnologies; H300), IgG2A mouse PSD95 1/350 (Thermo Scientific; 6G6-1C9) and IgG1 mouse Synaptophysin 1/1000 (EMD Millipore; 573822). Secondary antibodies conjugated with Alexa Fluor dyes (Invitrogen) were applied against the specific IgGs. All images were taken on a Zeiss 510 META confocal microscope using a 63x Plan-Apochromat objective. Z-stacks were taken at 0.8 [illegible]m intervals and maximal projections were made to give the images showed.

10. Kainic stimulation of Arc-Venus and WT mice.
Briefly, animals were injected intraperitoneal with a saline solution of kainic acid 20mg/kg at a volume of 10ml/kg and allowed to rest in a quiet dimly-lit room during 2 hours. Mice were then anesthetised by intraperitoneal injection of 0.1 mL of 20% pentobarbital sodium (Euthatal, Merial animal health Ltd.) and placed back in their cage until complete anaesthesia. When no responses were observed after pinching toes and tail, two tail samples were collected for PCR genotyping. Then the mouse was pinned down on a cork surface and the thorax was open. Once the heart was fully exposed, a small incision was performed in the right atrium and a needle was immediately inserted in the left ventricle for transcardiac injection of 10 mL of phosphate buffered saline (PBS, Fisher Scientific) followed by 10 mL of 4% paraformaldehyde (PFA, Alfa Aesar 16%, diluted 1:4 in PBS). Next, the brain was dissected out and post-fixed in 4% PFA at 4°C for 3-4h. Brains were always kept in the dark from the moment of dissection to preserve the fluorescence of the fusion proteins. The brain was then cryo-protected by incubation in 30% sucrose (VWR Chemicals, w/v in PBS) at 4°C for 72h. Brains were then embedded in optimal cutting temperature (OCT) medium (VWR international) inside a mould (Sigma-Aldrich). They were immediately frozen by placing the mould in a plastic beaker containing isopentane (Sigma-Aldrich), placed itself in liquid nitrogen. Once frozen, brains were stored at -80°C. Coronal sections (18 µm thickness) were cut with a cryostat (NX70 Thermo Fisher) and directly mounted on Superfrost Plus glass slides (Thermo scientific). Mounted sections were then left to dry at room temperature overnight and stored at -80°C the next day. Sections were stained with DAPI (Sigma, 1:10000. Mowiol was used to mount brain samples in high-refractive index mounting medium.

11. Electrophysiology in hippocampal slices
Previous studies show that disruption of Arc/Arg3.1 led to an impairment of synaptic long-term potentiation (LTP) and long-term depression (LTD) in the hippocampal CA1 area and dentate gyrus (Guzowski et al., 2000; Messaoudi et al., 2007; Shepherd et al., 2006). In view of these findings, we sought to explore if incorporation of the TAP and Venus tags into Arc led to disturbances of synaptic transmission and plasticity. Field excitatory postsynaptic potentials (fEPSPs) in the CA1 area of acute hippocampal slices were recorded using multi-electrode arrays as previously described (Coba et al., 2012; Kopanitsa et al., 2006). Baseline stimulation strength was adjusted to evoke a response that corresponded to ~40% of the maximal attainable fEPSP at the recording electrode located in proximal stratum radiatum. Amplitude of the negative part of fEPSPs was used as a measure of the synaptic strength. The effect of the mutation on the basal synaptic transmission was estimated by comparing areas under input-output curves (AUC_{I-O}) measured in individual slices. Paired stimulation with an interpulse interval of 50 ms was used to observe paired-pulse facilitation (PPF) in baseline conditions in the test pathway before LTP induction. PPF was calculated by dividing the amplitude of the fEPSP obtained in response to the second pulse by the amplitude of fEPSP evoked by the preceding pulse. To induce LTP, 10 bursts of baseline strength stimuli were administered at 5 Hz to test pathway with 4 pulses given at 100 Hz per burst (total 40 stimuli). LTP plots were scaled to the average of the first five baseline points. Normalization of LTP values was performed by dividing the fEPSP amplitude in the tetanized pathway by the amplitude of the control fEPSP at corresponding time points. Normalized LTP values averaged across the period of 61–65 min after theta-burst stimulation were used for statistical comparison.

12. Forebrain extracts and western blot

Forebrain extracts were solubilized in 1% DOC buffer at 0.38 g wet weight per 7 ml cold buffer with a glass Teflon Douncer homogenizer. The homogenate was incubated for 1 h at 4ºC and clarified at 50,000 *g* for 30 min at 4ºC. Protein concentration was quantified with the Bradford protein assay (BioRad). Samples containing equal amount of protein were subjected to reducing SDS electrophoresis (NUPAGE, Invitrogen) and transferred to polyvinyldifluoride membrane (Hybond™-P, GE Healthcare). The membranes were blocked in 5% non-fat milk, 0.01% Triton X-100 in PBS with the following antibodies: mouse Arc (Santa Cruz Biotechnology; C7), mouse SAP102 (NeuroMab; 75058), mouse PSD93 (NeuroMab; 75-057), mouse SAP97 (BD Biosciences; 610874/5), rabbit GluR1 (EMD Millipore; AB1504), and nNos (EMD Millipore; AB1632). Detection of signals was carried out using peroxidase-linked secondary IgGs (EMD Millipore; 12-348 and -349) and enhanced chemiluminescence (GE Healthcare). Quantification of the signals was performed using ImageJ open source program.

13. MAGUKs immunoprecipitation

Based on the LC-MS/MS data on the tandem purification of Arc complexes (Table S1), we verified the interaction of Arc with the MAGUKs PSD95 and checked whether the interaction of PSD95 and Arc was affected by the absence of PSD93. Mouse forebrains of the genotype indicated were homogenized in 1% DOC buffer following the same protocol as in the aforementioned TAP procedure. Equal amount of protein extracts was incubated for 2 h at 4 ºC with a mouse anti-Arc, -PSD95, -PSD93, -SAP97 or -SAP102. Captured complexes were then washed three times with DOC buffer and eluted with 1x NuPAGE LDS sample buffer (Invitrogen) supplemented with a reducing agent. Absence of PSD93 did not affect the interaction of Arc with PSD95 suggesting that PSD-95-Arc interaction is independent of PSD93.

14. Human and mouse Arc protein-protein interactions (PPIs).

We obtained mouse and human PPI interactions for the core 107 Arc genes from Table S1, using the publicly available databases: BioGrid (Chatr-Aryamontri et al., 2013),DIP (Salwinski et al., 2004), IntAct (Orchard et al., 2014),, MINT (Orchard et al., 2014), STRING (Jensen et al., 2009), UniProt (UniProt, 2014), BIND (Bader et al., 2003), mentha (Calderone et al., 2013) , APID (Alonso-Lopez et al., 2016) and HPRD (Prasad et al., 2009) using the Psicquic package (Aranda et al., 2011). In total we found 1447 reported mouse interactions, 1012 of these (~70%) were found in human.

15. Classification of Arc interactors according to their gene ontology process.

We performed enrichment analysis of the core 107 Arc genes from Table S1, for GO Biological Process (BP) terms using the topGO package (Alexa et al., 2006). The genome wide annotation package "org.Mm.eg.db" (Marc Carlson, org.Mm.eg.db: Genome wide annotation for Mouse. R package version 3.2.3) was used to obtain a background annotation set, leading to a background set of 23313 Entrez gene identifiers mapped onto 10623 BP terms.

P-values were calculated using the Exact Fisher test, and corrected for multiple hypothesis testing using the Benjamini Hochberg (BH) correction. In Table S6 we found the 107 Arc set enriched for 'ion transport', 'cation transport' and 'Synaptic transmission' (P=8.2x10-14, P=5.2x10-13 and P=5.7x10-13 respectively). These terms remained enriched in the down-regulated Arc subset from Table S7 (P=1.x10-3, P=9.2x10-3 and P=9.7x10-14 respectively). The upregulated subset from Table S7 was not found enriched for these terms. Instead this subset was found enriched for 'cellular potassium homeostasis', 'ATP metabolic process' and 'potassium ion homeostasis' (P=3.0x10-5, P=6.4x10-5 and 6.9x10-5 respectively). All the tables of the manuscript are compiled in http://wwwdev.genes2cognition.org/publications/tap-arc/.

16. Creation of human Arc interactor genesets

From the original 107 Arc interactor mouse proteins, we identified homologous human genes based on the Mouse Genome Informatics database (http://www.informatics.jax.org/faq/ORTH_dload.shtml). After mapping and manual curation of gene identifiers, we found 124 human Arc interactors RefSeq genes, of which 13 were associated with Arc with increased values in the PSD95$^{-/-}$ mouse, 37 of which were associated with Arc with decreased values, and 18 of which were known direct interactors of PSD95 (Table S7).

17. Mammalian Phenotype Ontology of Arc interactors

We performed enrichment analysis of the core 107 Arc genes from Table S1, for Mammalian Phenotype (MP) terms using the Ontology package topOnto (https://github.com/statbio/topOnto). MGI's 'All Genotypes and Mammalian Phenotype' annotation file (ftp://ftp.informatics.jax.org/pub/reports/MGI_PhenoGenoMP.rpt) was used to obtain a background annotation set, leading to a background set of 8770 Entrez gene identifiers mapped onto 8813 MP terms.

P-values were calculated using the Exact Fisher test, and corrected for multiple hypothesis testing using the Bonferroni correction (at the 0.01 '***' and 0.05 '**' significance levels), and the Benjamini Hochberg (BH) correction. In Table S1 0 we found the 107 Arc set enriched for 'abnormal synaptic transmission', 'abnormal CNS synaptic transmission', 'abnormal nervous system physiology' (P=4.4x10-16, P=7.8x10-15 and 9.5x10-15 respectively). These terms remained enriched using the down-regulated Arc subset from Table S7 (P=7.7x10-12, P=1.4x10-12 and P=1.3x10-10 respectively). The upregulated subset from Table S7 was not found enriched for these terms.

18. Network building
Interactions were mined from publicly available databases: BioGrid (Chatr-Aryamontri et al., 2013), DIP (Salwinski et al., 2004), , (Orchard et al., 2014), MINT (Orchard et al., 2014)STRING (Jensen et al., 2009), UniProt (2014), BIND (Bader et al., 2003), mentha (Calderone et al., 2013) using the Psicquic software package (Aranda et al., 2011). The network is visualised using Visone (http://visone.info/html/).

19. Human genetic analysis
***De novo* CNV enrichment analysis**
For each gene set, the number of genes hit by 34 *de novo* CNVs from schizophrenia case were compared those hit by 59 *de novo* CNVs from unaffected Icelandic controls. A gene was counted as being hit by a CNV if the CNV overlapped any part of its length (human genome Build 36.3). To overcome biases related to gene and CNV size, and to control for differences between studies and genotyping chips, the following logistic regression models were fitted to the combined set of CNVs:
(a) logit (pr(case)) = CNV size + total number of genes hit
(b) logit (pr(case)) = CNV size + total number of genes hit + number of genes hit in gene set

Comparing the change in deviance between models (a) and (b), a one-sided test for an excess of genes in the gene set being hit by case CNVs was performed. The genetic data used for this analysis is described in (Kirov et al., 2012). Note that while a small number of the protein groups from Table S1 correspond to multiple proteins/genes, their presence does not influence the analysis as none were hit by either case or control CNVs.

***De novo* mutation exome sequencing datasets**

Disease	*N* trios	*N* LoF mutations	*N* nonsynonymous mutations	Sources (PMIDs)
Autism	3,985	579	3446	25363760 23849776 25363768
Epilepsy	356	58	341	25262651
ID	192	67	259	23033978 25356899 23020937
Schizophrenia	1024	114	756	24463507 21743468 23911319 24776741 23042115

The Swedish schizophrenia case/control association data were based on summary statistics from a previously described dataset (Purcell et al., 2014). We used PLINK/Seq (http://atgu.mgh.harvard.edu/plinkseq/) to uniformly re-annotate all mutations with respect to RefSeq genes. Consistent with the previously reported work, for all *de novo* studies we focused on two classes of mutation: 1) gene disruptive (or loss-of-function, LoF) mutations (i.e. nonsense, essential splice site or frameshift indels); 2) a broad class of all nonsynonymous mutations (including disruptive mutations). For the case/control schizophrenia study, we focused on 1) rare gene disruptive mutations with a sample frequency of less than 0.1%; 2) rare (<0.1%) nonsynonymous mutations that were predicted *in silico* to have a likely deleterious effect on protein function, following (Purcell et al., 2014). Analysis of silent mutations was included as control (Table S9). For the analysis of *de novo* mutations, we used DNENRICH, a freely-available software previously described (Fromer et al., 2014) (https://psychgen.u.hpc.mssm.edu/dnenrich/). Briefly, DNENRICH estimates the observed number of *de novo* mutations per gene or geneset by a genomic permutation strategy, which controls for gene size and structure, sequence coverage and local trinucleotide mutation rate. Significance of the enrichment in the rate and recurrence of mutations was assessed empirically by permutation. For the case/control data, we used the SMP algorithm (Purcell et al., 2014) which is part of the PLINK/Seq package (http://atgu.mgh.harvard.edu/plinkseq/). Briefly, the test for enrichment of a set of genes is based on the sum of gene-level case/control burden statistics relative to the exome-wide excess of burden in cases. Significance is assessed by permutation, comparing the observed distribution against 10,000 null replicates (created by shuffling case/control labels within groups matched for ancestry and technical variables).

Neuropsychiatric genetic analyses: multiple testing correction.
We first sought to verify the initial finding of Arc complex enrichment in de novo CNVs that has formed the foundation for all subsequent studies; this we correct for the 4 Arc complex gene-sets tested. We then turned to the analysis of rare coding variants. While there is consistent evidence for disease-variant enrichment across multiple independent studies (based on preliminary Arc data), these associations are modest. We therefore combine enrichment p-values from each rare-variant dataset to provide an overall summary of the evidence. These summary p-values are corrected for 12 tests: 4 Arc complex gene-sets tested x 2 classes of mutation (LoF and NS), plus the 4 de novo CNV tests already performed. This correction is likely to be conservative, as LoF and NS mutations are not independent sets. Finally, we present enrichment p-values for individual datasets, correcting for 52 tests (4 Arc complex gene-sets x LoF and NS mutations x 5 individual datasets, plus the 12 tests already performed).

20. Human cognitive ability phenotype

In the case of the CAGES consortium a general factor of cognitive ability (*g* or intelligence) was derived separately in each cohort. For the Lothian Birth Cohort of 1921 (LBC1921) consists of 550 individuals (females = 316) with a mean age of 79.1 years (SD = 0.6) (Deary et al., 2009; Deary et al., 2004; Scottish Council for Research in Education, 1933). The tests used to derive a measure of intelligence were the Moray House Test (MHT) (Scottish Council for Research in Education, 1933), Raven's Standard Progressive Matrices (Raven et al., 1977), the Wechsler Logical Memory scores (Wechsler, 1987), phonemic verbal fluency (Lezak et al., 2004), and the National Adult Reading Test (NART) (Nelson and Willison, 1991). The Lothian Birth Cohort of 1936 (LBC1936) includes data on 1091 individuals (females = 543) with a mean age of 69.5 years (SD = 0.8) (Deary et al., 2007). The tests used to measure intelligence in the LBC1936 were the Digit Symbol Coding, Block Design, Matrix Reasoning, Digit Span Backwards, Symbol Search and the Letter Number Sequencing from the Wechsler Adult Intelligence Scale (Wechsler, 1998) and the NART (Nelson and Willison, 1991). The Aberdeen Birth Cohort of 1936 (ABC1936) has data on 498 (females=255) older individuals (mean age = 64.6, SD = 0.9 years of age). In order to provide a measure of intelligence the following tests were used. The Rey Auditory Verbal Learning Test (Lezak et al., 2004), the Uses of Common objects Test (Guildford et al., 1978), Raven's Standard Progressive Matrices (Raven et al., 1977) and Digit Symbol (Wechsler, 1981) along with the NART (Nelson and Willison, 1991). In each of these three cohorts intelligence was measured by subjecting the raw scores from the tests used to a principal components analysis within each cohort. The first unrotated component was extracted using regression and was used as the dependent variable in a linear regression with age and sex being used as predictors. The standardised variables from this model were then used in the subsequent analysis (Hill et al., 2014a). In the Manchester and Newcastle cohorts consist of 1563 individuals (female = 1108) with a median age of 65 ranging from 44 – 93 years of age. The tests used were the four parts of the Culture Fair Test (Cattell and Cattell, 1960) and the two parts of the Alice Heim Test 4 (Heim, 1970) were used. Age and sex were controlled for using residuals which were then standardised. These were then subjected to a maximum likelihood factor analysis. These was then summed with the age and sex controlled standardised residuals from the Mill Hill vocabulary test (Raven, 1965) with the mean being used as the measure of intelligence (Hill et al., 2014a). In the replication sample consisted of the BATS cohort which consists of 2062 individuals (females = 1093) with a mean age of 16.6 years (SD = 1.5). The test used as a measure of intelligence was the full scale IQ score from the Multidimensional Aptitude Battery (Jackson, 1984) corrected for age and sex with regression. The standardised residuals from this model were used in subsequent genetic analysis.

Genome wide association had been carried out in each cohort of CAGES using Mach2QTL (Li et al., 2010) before being meta-analysed in METAL (Willer et al., 2010) using an inverse variance weighted model. SNPs were then assigned to genes based on their position in the UCSC human genome browser hg 18 assembly with a 50kb boundary around each gene to capture any regulatory elements. A gene based statistic was derived using VEGAS (Liu et al., 2010) to control for the number of SNPs assigned to each gene as well as patterns of linkage disequilibrium. In order to test the principal hypothesis that the genes of the Arc complex (See Table S11) will show a greater association, as a set, than those drawn from across the genome, each gene based p value was –log10 transformed and rank ordered. Gene Set Enrichment Analysis (GSEA) (Subramanian et al., 2005; Wang et al., 2007), a competitive test of enrichment, was then used to determine if the gene identifiers for the Arc gene set fall higher in the genome-wide rankings than would be expected by chance alone (Hill et al., 2014a; Hill et al., 2014b). This was done for each set by deriving a Kolmogorov-Smirnov (K-S) statistic weighted by the p-values of the gene based statistic in order to take into account both the ranks and the distance between ranks. Following this the genome wide ranked set was permuted and the K-S statistic calculated again. Statistical significance was established by using 15,000 permutations of the genome wide ranked set with the p-value describing the proportion of permuted K-S tests smaller than the original un-permuted K-S statistic. Statistical significance was set at < 0.05 and FDR <0.25 (Subramanian et al., 2005; Wang et al., 2007).

21. Statistics

Hippocampal slices

In electrophysiological experiments, since several slices were routinely prepared from every mouse, AUC_{I-O}, PPF and LTP values were compared between wild-type and mutant mice using the two-way nested ANOVA with genotype (group) and mice (sub-group) as fixed and random factors correspondingly (STATISTICA v. 10, StatSoft, Inc., Tulsa, OK, USA). DF error was computed using the Satterthwaite method and main genotype effect was considered significant if $P < 0.05$. Graph plots and normalization were performed using OriginPro 8.5 (OriginLab, Northampton, MA, USA). Electrophysiological data are presented as the mean ± s.e.m. with n and N indicating number of slices and mice respectively.

Protein quantification

Comparisons between two groups were performed using two-tailed Mann-Whitney U test. Significance was accepted to $P <$ 0.05. Data are presented as mean ± s.e.m.

Genetics

P values were corrected by a Bonferroni multiple test. We initially performed 4 tests for the 4 Arc complexes for CNVs, 8 tests for Lof/NS combined analyses, and 40 tests for LoF/NS in individual studies. However, at each successive stage we also accounted for all of the tests previously performed, giving a total of 4 tests for de novo CNVs, 12 tests for combined NS/LoF and 52 tests for NS/LoF studies. Fisher's method was used to combine p-values from the 5 independent genetic datasets ('Combined' p-value for LoF and NS mutations).

22. Known Arc interactors.
Arc interactors were manually curated from literature. Arc interactors previously reported have been manually annotated. Protein IDs (UniProt accession numbers) as well as protein and gene names are shown. PubMed ID and the interaction detection method are also indicated.

Protein ID	Gene name	Protein name	PMID
P28652	*Camk2b*	Calcium/calmodulin-dependent protein kinase type II subunit beta	22579289
P35438;P35438-2	*Grin1*	Glutamate receptor ionotropic, NMDA 1	16635246 10862698
G3X9V4;Q01097	*Grin2b*	Glutamate receptor ionotropic, NMDA 2B	10862698 25864631
P49768	*Psen1*	Presenilin-1	22036569
Q62108;Q62108-3;Q62108-2;G3UZL5	*Dlg4*	Disks large homolog 4	19455133
Q80TN1;P11798; F8WIS9	*Camk2a*	Calcium/calmodulin-dependent protein kinase type II subunit alpha	22579289
P39054	*Dnm2*	Dynamin-2	17088211
Q62419	*Sh3gl1*	Endophilin-A2	17088211
Q62421	*Sh3gl3*	Endophilin-A3	17088211
O88602	*Cacng2*	TARPg2	25864631
Q9D415	*Dlgap1*	Disks large-associated protein 1	25864631
Q8R5H6	*Wasf1*	Wiskott-Aldrich syndrome protein family member 1	25864631
P35436	*Grin2a*	Glutamate receptor ionotropic, NMDA 2A	25864631
Q5DU25	*Iqsec2*	IQ motif and SEC7 domain-containing protein 2	25864631

23. Oligos

Oligo name	Sequence 5'3
ArcHA1XbaR	CTGC TCT AGA TTC AGG CTG GGT CCT GTC ACT G
ArcHA1XhoF	ATC TCG CTC GAG GGG AGG TCT TCT ACC GTC TGG AG
ArcHA2BglR	GACCT AGA TCT GC GGCC GC GGG GCC AGG AGG TGC CAG GAT GTC AGG TC
ArcHA2Acc65F	CTACGG GGT ACC AGG GGC CAG CCC AGG GTC CCC AG
ArcHA3NdeF	GGG TAA TCTC CA TAT G GGA GGC TTA GGC TAG GCA CAG AC
ArcHA3PmlR	CCG GCG TA CAC GTG CCC TGG TCC CAG CCA TGA TTC ATA AG
ArcHA4AscF	CATTT G GCG CGC C CCA ATA GGT CAT CAC AAC TGC CAT G
ArcHA4PmeR	AGC TTT G TTT AAA C GCC CTT GCA CCT CTT GCT GCA C
PneoF1	TAC AAA TAA GC AAT AGC ATC AC
Pneo3F	GCC TCT GAG CTA TTC CAG AAG TAG
Pneo3R	CCT GAC TAG GGG AGG AGT AGA AG
Arc10R	GGG CTT CTT ATG TTC AGT C
Arc1F	CGG GAC CTG TAC CAG ACA CTG
Arc1R	GCC ATG GCT GAG TCA CGG AG
Arc2F	GGA GCG AGA GCT GAA AGG GTT G
Arc2R	GGG GCC AGG AGG TGC CAG GAT GTC
Arc3R	CCC TCA GCA TCT CTG CTT TAG C
Arc4R	GCC AGG ACT AGG TTG GAC AGT CTG
Arc5F	CCC TCC TGG ATC TAG TGG TGA GC
Arc5R	GGGCATCCACTCACATAACTACTCG
Arc6F	GCC CCT GCC CAG CCT GAT CTT TC
Arc6R	CCC CGA GAC ACT ACT CTG AGC
Arc7F	GGG TGG ACA GTT GGT CAG AAG G
Arc7R	CCC TCT GAC CAC TGC ATT TTC C
Arc8F	CCC AGT GCT TTC TCA GCC TTC ATG
Arc8R	GGT CAG TGG GTG AGT AGA TGT CTG
Arc9F	CGG AGG CAC TCA CAC CTG
Arc9R	GGT TGT GTG GCA GTT GTG AGT C

Arc10F	GCT CTT ACC AGC GAG
Arc11F	GGG CAG ATG CTA AAG CAG
Arc11R	CGG CTG GGT GTG AGG ACT CAG
Venus F	CGG CGC GCT AGC GGT GGC GGT AGT ATG GTG AGC AAG GGC GAG GAG C
VenusR	AAC CGT CCT GCA GGT CAC TTG TAC AGC TCG TCC ATG CCG AG
AcExon F2	CAG AGG ATG AGA CGG AGG CAC TC
ArcUTR R2	AGG GGC TTC TTG ATG TTC AGT CC

References

(2014). Activities at the Universal Protein Resource (UniProt). Nucleic acids research *42*, D191-198.

Alexa, A., Rahnenfuhrer, J., and Lengauer, T. (2006). Improved scoring of functional groups from gene expression data by decorrelating GO graph structure. Bioinformatics *22*, 1600-1607.

Alonso-Lopez, D., Gutierrez, M.A., Lopes, K.P., Prieto, C., Santamaria, R., and De Las Rivas, J. (2016). APID interactomes: providing proteome-based interactomes with controlled quality for multiple species and derived networks. Nucleic acids research *44*, W529-535.

Aranda, B., Blankenburg, H., Kerrien, S., Brinkman, F.S., Ceol, A., Chautard, E., Dana, J.M., De Las Rivas, J., Dumousseau, M., Galeota, E., *et al.* (2011). PSICQUIC and PSISCORE: accessing and scoring molecular interactions. Nature methods *8*, 528-529.

Bader, G.D., Betel, D., and Hogue, C.W. (2003). BIND: the Biomolecular Interaction Network Database. Nucleic acids research *31*, 248-250.

Binns, D., Dimmer, E., Huntley, R., Barrell, D., O'Donovan, C., and Apweiler, R. (2009). QuickGO: a web-based tool for Gene Ontology searching. Bioinformatics *25*, 3045-3046.

Calderone, A., Castagnoli, L., and Cesareni, G. (2013). mentha: a resource for browsing integrated protein-interaction networks. Nature methods *10*, 690-691.

Cattell, R.B., and Cattell, A.K.S. (1960). The individual or group Culture Fair Intelligence Test (Champaign, Ill: IPAT).

Chatr-Aryamontri, A., Breitkreutz, B.J., Heinicke, S., Boucher, L., Winter, A., Stark, C., Nixon, J., Ramage, L., Kolas, N., O'Donnell, L., *et al.* (2013). The BioGRID interaction database: 2013 update. Nucleic acids research *41*, D816-823.

Chowdhury, S., Shepherd, J.D., Okuno, H., Lyford, G., Petralia, R.S., Plath, N., Kuhl, D., Huganir, R.L., and Worley, P.F. (2006). Arc/Arg3.1 interacts with the endocytic machinery to regulate AMPA receptor trafficking. Neuron *52*, 445-459.

Coba, M.P., Komiyama, N.H., Nithianantharajah, J., Kopanitsa, M.V., Indersmitten, T., Skene, N.G., Tuck, E.J., Fricker, D.G., Elsegood, K.A., Stanford, L.E., *et al.* (2012). TNiK is required for postsynaptic and nuclear signaling pathways and cognitive function. The Journal of neuroscience : the official journal of the Society for Neuroscience *32*, 13987-13999.

Cox, J., and Mann, M. (2008). MaxQuant enables high peptide identification rates, individualized p.p.b.-range mass accuracies and proteome-wide protein quantification. Nature biotechnology *26*, 1367-1372.

Deary, I.J., Gow, A.J., Taylor, M.D., Corley, J., Brett, C., Wilson, V., Campbell, H., Whalley, L.J., Visscher, P.M., Porteous, D.J., *et al.* (2007). The Lothian Birth Cohort 1936: a study to examine influences on cognitive ageing from age 11 to age 70 and beyond. BMC Geriatr *7*, 28.

Deary, I.J., Whalley, L.J., and Starr, J.M. (2009). A Lifetime of Intelligence, Follow-up Studies of the Scottish Mental Surveys of 1932 and 1947 (Washington, DC: American Psychological Association).

Deary, I.J., Whiteman, M.C., Starr, J.M., Whalley, L.J., and Fox, H.C. (2004). The impact of childhood intelligence on later life: Following up the Scottish Mental Surveys of 1932 and 1947. J Pers Soc Psychol *86*, 130-147.

Fernandez, E., Collins, M.O., Uren, R.T., Kopanitsa, M.V., Komiyama, N.H., Croning, M.D., Zografos, L., Armstrong, J.D., Choudhary, J.S., and Grant, S.G. (2009). Targeted tandem affinity purification of PSD-95 recovers core postsynaptic complexes and schizophrenia susceptibility proteins. Molecular systems biology *5*, 269.

Frank, R.A., Komiyama, N.H., Ryan, T.J., Zhu, F., O'Dell, T.J., and Grant, S.G. (2016). NMDA receptors are selectively partitioned into complexes and supercomplexes during synapse maturation. Nat Commun *7*, 11264.

Fromer, M., Pocklington, A.J., Kavanagh, D.H., Williams, H.J., Dwyer, S., Gormley, P., Georgieva, L., Rees, E., Palta, P., Ruderfer, D.M., *et al.* (2014). De novo mutations in schizophrenia implicate synaptic networks. Nature *506*, 179-184.

Guildford, J.P., Christensen, P.R., Merrifield, P.R., and Wilson, J., C (1978). Alternate uses: manual of instructions and interpretation (Orange, Calif: Sheridan Psychological Service).

Guzowski, J.F., Lyford, G.L., Stevenson, G.D., Houston, F.P., McGaugh, J.L., Worley, P.F., and Barnes, C.A. (2000). Inhibition of activity-dependent arc protein expression in the rat hippocampus impairs the maintenance of long-term potentiation and the consolidation of long-term memory. The Journal of neuroscience : the official journal of the Society for Neuroscience *20*, 3993-4001.

Heim, A. (1970). AH 4 Group Test of General Intelligence Manual, (Windsor, UK: NFER Publishing Company).

Hill, W.D., Davies, G., van de Lagemaat, L.N., Christoforou, A., Marioni, R.E., Fernandes, C.P.D., Liewald, D.C., Croning, M.D.R., Payton, A., Craig, L.C.A., *et al.* (2014a). Human cognitive ability is influenced by genetic variation in components of postsynaptic signalling complexes assembled by NMDA receptors and MAGUK proteins. Transl Psychiatry *4*, e341.

Hill, W.D., de Leeuw, C., Davies, G., Liewald, D.C.M., Payton, A., Craig, L.C., J.W., L., Horan, M., Ollier, W., Starr, J.M., *et al.* (2014b). Functional Gene Group Analysis Indicates No Role for Heterotrimeric G Proteins in Cognitive Ability. PloS one *9*.

Jackson, D.N. (1984). Multidimensional Aptitude Battery manual., Vol 1 (Port Huron, MI: Research Psychologists Press).

Jensen, L.J., Kuhn, M., Stark, M., Chaffron, S., Creevey, C., Muller, J., Doerks, T., Julien, P., Roth, A., Simonovic, M., *et al.* (2009). STRING 8--a global view on proteins and their functional interactions in 630 organisms. Nucleic acids research *37*, D412-416.

Kirov, G., Pocklington, A.J., Holmans, P., Ivanov, D., Ikeda, M., Ruderfer, D., Moran, J., Chambert, K., Toncheva, D., Georgieva, L., *et al.* (2012). De novo CNV analysis implicates specific abnormalities of postsynaptic signalling complexes in the pathogenesis of schizophrenia. Molecular psychiatry *17*, 142-153.

Kopanitsa, M.V., Afinowi, N.O., and Grant, S.G. (2006). Recording long-term potentiation of synaptic transmission by three-dimensional multi-electrode arrays. BMC Neurosci *7*, 61.

Lezak, M., Howieson, D.B., and Loring, D.W. (2004). Neuropsychological Assessment, 4 edn (New York: Oxford University Press, USA).

Li, Y., Willer, C.J., Ding, J., Scheet, P., and Abecasis, G.R. (2010). MaCH: using sequence and genotype data to estimate haplotypes and unobserved genotypes. Genetic epidemiology *34*, 816-834.

Liu, J.Z., McRae, A.F., Nyholt, D.R., Medland, S.E., Wray, N.R., Brown, K.M., Hayward, N.K., Montgomery, G.W., Visscher, P.M., Martin, N.G., *et al.* (2010). A versatile gene-based test for genome-wide association studies. American journal of human genetics *87*, 139-145.

Liu, P., Jenkins, N.A., and Copeland, N.G. (2003). A highly efficient recombineering-based method for generating conditional knockout mutations. Genome Res *13*, 476-484.

Messaoudi, E., Kanhema, T., Soule, J., Tiron, A., Dagyte, G., da Silva, B., and Bramham, C.R. (2007). Sustained Arc/Arg3.1 synthesis controls long-term potentiation consolidation through regulation of local actin polymerization in the dentate gyrus in vivo. The Journal of neuroscience : the official journal of the Society for Neuroscience *27*, 10445-10455.

Nelson, H.E., and Willison, J. (1991). National Adult Reading Test (NART) Test Manual, 2nd edn (Windsor: NFER-Nelson).

Orchard, S., Ammari, M., Aranda, B., Breuza, L., Briganti, L., Broackes-Carter, F., Campbell, N.H., Chavali, G., Chen, C., del-Toro, N., *et al.* (2014). The MIntAct project--IntAct as a common curation platform for 11 molecular interaction databases. Nucleic acids research *42*, D358-363.

Prasad, T.S., Kandasamy, K., and Pandey, A. (2009). Human Protein Reference Database and Human Proteinpedia as discovery tools for systems biology. Methods Mol Biol *577*, 67-79.

Purcell, S.M., Moran, J.L., Fromer, M., Ruderfer, D., Solovieff, N., Roussos, P., O'Dushlaine, C., Chambert, K., Bergen, S.E., Kahler, A., *et al.* (2014). A polygenic burden of rare disruptive mutations in schizophrenia. Nature *506*, 185-190.

Raven, J.C. (1965). The Mill Hill vocabulary scale (London: H.K. Lewis).

Raven, J.C., Court, J.H., and Raven, J. (1977). Manual for Raven's Progressive Matrices and Vocabulary Scales (London: H.K. Lewis).

Salwinski, L., Miller, C.S., Smith, A.J., Pettit, F.K., Bowie, J.U., and Eisenberg, D. (2004). The Database of Interacting Proteins: 2004 update. Nucleic acids research *32*, D449-451.

Scottish Council for Research in Education (1933). The intelligence of Scottish children: A national survey of an age-group (London: University of London Press).

Shepherd, J.D., Rumbaugh, G., Wu, J., Chowdhury, S., Plath, N., Kuhl, D., Huganir, R.L., and Worley, P.F. (2006). Arc/Arg3.1 mediates homeostatic synaptic scaling of AMPA receptors. Neuron *52*, 475-484.

Steward, O., Wallace, C.S., Lyford, G.L., and Worley, P.F. (1998). Synaptic activation causes the mRNA for the IEG Arc to localize selectively near activated postsynaptic sites on dendrites. Neuron *21*, 741-751.

Subramanian, A., Tamayo, P., Mootha, V.K., Mukherjee, S., Ebert, B.L., Gillette, M.A., Paulovich, A., Pomeroy, S.L., Golub, T.R., Lander, E.S., *et al.* (2005). Gene set enrichment analysis: a knowledge-based approach for interpreting genome-wide expression profiles. Proc Natl Acad Sci U S A *102*, 15545-15550.

UniProt, C. (2014). Activities at the Universal Protein Resource (UniProt). Nucleic acids research *42*, D191-198.

Valor, L.M., Charlesworth, P., Humphreys, L., Anderson, C.N., and Grant, S.G. (2007). Network activity-independent coordinated gene expression program for synapse assembly. Proceedings of the National Academy of Sciences of the United States of America *104*, 4658-4663.

Wang, K., Li, M., and Bucan, M. (2007). Pathway-based approaches for analysis of genomewide association studies. American journal of human genetics *81*, 1278-1283.

Wechsler, D. (1981). Manual for the Wechsler adult intelligence scale – Revised. (New York: The Psychological Corporation).

Wechsler, D. (1987). Wechsler Memory Scale—Revised (New York: Psychological Corporation).

Wechsler, D. (1998). Wechsler Adult Intelligence Scale - third edition (London: The Psychological Corporation).

Willer, C.J., Li, Y., and Abecasis, G.R. (2010). METAL: fast and efficient meta-analysis of genomewide association scans. Bioinformatics *26*, 2190-2191.
